# Cloning and Phylogenetic Analysis of *Brassica napus* L. *Caffeic Acid O-Methyltransferase 1* Gene Family and Its Expression Pattern under Drought Stress

**DOI:** 10.1371/journal.pone.0165975

**Published:** 2016-11-10

**Authors:** Wei Li, Junxing Lu, Kun Lu, Jianglian Yuan, Jieheng Huang, Hai Du, Jiana Li

**Affiliations:** 1 Chongqing Engineering Research Centre for Rapeseed, College of Agronomy and Biotechnology, Southwest University, Chongqing, 400715, PR China; 2 Chongqing Key Laboratory of Molecular Biology of Plants Environment Adaption, College of Life Sciences, Chongqing Normal University, Chongqing, 401331, PR China; Huazhong University of Science and Technology, CHINA

## Abstract

For many plants, regulating lignin content and composition to improve lodging resistance is a crucial issue. Caffeic acid O-methyltransferase (COMT) is a lignin monomer-specific enzyme that controls S subunit synthesis in plant vascular cell walls. Here, we identified 12 *BnCOMT1* gene homologues, namely *BnCOMT1-1* to *BnCOMT1-12*. Ten of 12 genes were composed of four highly conserved exons and three weakly conserved introns. The length of intron I, in particular, showed enormous diversification. Intron I of homologous *BnCOMT1* genes showed high identity with counterpart genes in *Brassica rapa* and *Brassica oleracea*, and intron I from positional close genes in the same chromosome were relatively highly conserved. A phylogenetic analysis suggested that *COMT* genes experience considerable diversification and conservation in *Brassicaceae* species, and some *COMT1* genes are unique in the *Brassica* genus. Our expression studies indicated that *BnCOMT1* genes were differentially expressed in different tissues, with *BnCOMT1-4*, *BnCOMT1-5*, *BnCOMT1-8*, and *BnCOMT1-10* exhibiting stem specificity. These four *BnCOMT1* genes were expressed at all developmental periods (the bud, early flowering, late flowering and mature stages) and their expression level peaked in the early flowering stage in the stem. Drought stress augmented and accelerated lignin accumulation in high-lignin plants but delayed it in low-lignin plants. The expression levels of *BnCOMT1s* were generally reduced in water deficit condition. The desynchrony of the accumulation processes of total lignin and *BnCOMT1*s transcripts in most growth stages indicated that *BnCOMT1s* could be responsible for the synthesis of a specific subunit of lignin or that they participate in other pathways such as the melatonin biosynthesis pathway.

## Introduction

Rapeseed (*Brassica napus* L.) is a crucial economic crop worldwide, largely stemming from steady demands for rapeseed oil consumption and biodiesel production. Concerns over rapeseed production are growing since the agricultural yields of rapeseed are severely restricted by plant lodging and disease, which have even resulted in production declines. The lignification levels of the stem and root in rapeseed closely correlate with plant lodging and wreckage, as lignification influences the rigidity and arrangement of vascular tissues. Lignin is a structural heteropolymer abundant in vascular plants. Lignin provides intercellular hydrophobicity [1] and mechanical support for plant tissues [2] and effectively defends against biotic and abiotic stresses [3]. The skeleton frame of lignin in angiosperm mainly comprises guaiacyl (G) and syringyl (S) monolignol [4] with different degrees of methoxylation. O-methylation of C3 and C5 is an essential step in the lignin biosynthesis pathway and involves G and S subunit synthesis. A proper S/G ratio is beneficial for improving plant resistance to stress.

In dicots, caffeic acid O-methyltransferase (COMT) is considered a multifunctional enzyme with high substrate promiscuity, as it methylates caffeoyl and 5-hydroxy coniferyl alcohols, aldehydes and free acids [5], and catalyses C5 methoxylation, which is involved in the preferential derivation of S subunits. The *COMT* gene was first cloned and identified as a gene family in aspen (*Populus tremula*) [6]. Subsequent research efforts provided insight into the functions of *COMT* genes. In the model-plant *Arabidopsis thaliana*, the *COMT1* (*At5g54160*) knockout mutant line *Atomt1* showed no readily visible phenotypical characteristics compared with those of wild-type plants. Sinapyl (S) alcohol-derived substructures, however, were substituted by 5-hydroxyconiferyl alcohol (5OHG)-derived moieties [7]. *COMT* genes in other plants have also attracted considerable attention. The *brown midrib* (*bmr*) mutants *(bmr12*, *bmr18* and *bmr26*) in sorghum (*Sorghum bicolor*) with brown vascular tissue in the leaves and stem were identified as mutant alleles of the *COMT* gene, containing reduced levels of COMT transcripts [8] which resembles the changes observed in *bmr3* of maize (*Zea mays*) [9]. Similar to the *COMT* mutants, down-regulation of *COMT* generally results in a reduction of S units but not of total lignin content in a variety of species, including poplar (*Populus trichocarpa* x *P*. *deltoides*) [10], alfalfa (*Medicago sativa*) [11], maize [12], *A*. *thaliana* [13] and switchgrass (*Panicum virgatum*) [14]. A concomitant increase in the precursor 5-hydroxyguaiacyl units and a decrease in the S/G ratio are also frequently observed. Interestingly, a recent study implied that AtOMT methylates N-acetylserotonin into melatonin possessing N-acetylserotonin O-methyltransferase (ASMT) activity [15], results that were also evaluated and identified in rice [16]. Melatonin is implicated in pleiotropic functions during plants growth and development [17]. In addition, the importance of melatonin in plant protection against stresses including water [18], cold [19], high temperature [20], salt [21], oxidative stress [22], senescence [23], herbicides [22], and pathogens [24] has been reported. COMT is also thought to be responsible for the last step of melatonin biosynthesis by catalysing serotonin into melatonin with an intermediate 5-methoxytryptamine (5-MT) in plants [15].

*B*. *napus* (*2n* = 38, AACC) is an allotetraploid with two fused diploid genomes, an A-genome progenitor *Brassica rapa* (*2n* = 20, AA) and a C-genome progenitor *Brassica oleracea* (*2n* = 18, CC) [25]. However, the inherent level of duplication within *B*. *napus*, with an amphidiploids genome, presents extra barriers in terms of identifying homologous regions across relative species [26]. Defining the extent of genome duplication in amphidiploids genomes before collinearity analysis is essential [26]. Moreover, researches focused on the relationship across species would contribute to deciphering genome evolution and duplication, as well as divergence of gene function.

The structural and functional characteristics of *COMTs* have been annotated in several species, such as *Arabidopsis* [7], alfalfa [11], poplar [10], and maize [12]. However, the family members and phylogenetic relationships of COMTs in *Brassicaceae* remain unknown. Here, we identified potential members of the *COMT* family in three highly homogenous *Brassica* species (*B*. *napus*, *B*. *rapa*, and *B*. *oleracea*) as well as *Arabidopsis*. We investigated their evolutionary divergence and conservation. Additionally, we studied *BnCOMT1s* expression patterns in various tissues and life stages.

## Materials and Methods

### *BnCOMT1* genes identification

To identify the coding sequences (CDS) of *COMT* homologues in rapeseed, we followed previously described methods [27] with minor modifications. In brief, the full-length CDS of 14 *Arabidopsis OMT* genes were downloaded from TAIR (Version 10) (www.arabidopsis.org) and used to simultaneously search against the *Brassica* database (BRAD, http://brassicadb.org/brad/index.php), Phytozome v10.0 (http://phytozome.jgi.doe.gov/pz/portal.html), and the *Brassica napus* Genome Resource (http://www.genoscope.cns.fr/brassicanapus/). We only retrieved CDSs with e-value hits lower than 10^-4^ or with a gene sequence identity higher than 85%. These CDSs were further filtered, clustered, and assembled. The resulting contigs and singletons were reciprocally searched against the *Arabidopsis* database to identify the best hit among all 14 *AtOMT* genes for each contig and singleton, which identified putative orthologues. Syntenic genes were also found in BRAD.

### Plant growth and gene cloning

Wild type rapeseed (ZS11) plants were grown at Chongqing (Chongqing Rapeseed Engineering Research Centre, Southwest University, China) in a field under natural conditions. Young stems in the early flowering stage were harvested for RNA isolation using TRIzol (Invitrogen, USA) and RNase-free DNase I (Fermentas, Canada). RNA integrity was evaluated by electrophoresis on an agarose gel and quantified using a NanoDrop 1000 (NanoDrop Technologies, Inc., USA). Total RNA (2.5 μg) was used to synthesize cDNAs with the PrimeScript RT Reagent Kit (TaKaRa, Dalian). PCR was conducted in a 50-μL final volume and included 0.5 μL of cDNA template, 10× Pfu buffer, 10 mM deoxynucleotide triphosphates mix (dNTPs), 400 nM of each primer, and 1.25 units of *Pfu* DNA polymerase using an *ExTaq* Hot Start Kit (TaKaRa, Dalian). The PCR conditions included an initial denaturation at 94°C for two min, followed by 35 cycles of 94°C for 30 s, 63°C for 30 s, 72°C for one min per kb, with a final extension at 72°C for 10 min. The primers used are listed in S1 Table. The PCR products were gel purified using the Transgene Gel Extraction Kit (Transgene, Beijing), cloned into T vector pMD19 (Simple) (TaKaRa, Dalian) and sequenced from both ends.

### Phylogenetic tree construction and bioinformatics

The *AtCOMT* sequences were downloaded from TAIR10. To identify *COMT* from other species, we first aligned the 14*AtOMTs* and generated a hidden Markov model (HMM); second, we performed an HMM-based search (http://hmmer.janelia.org/) for similar peptide sequences in the sequenced genomes stored in Phytozome v10.0, NCBI (http://www.ncbi.nlm.nih.gov/), and we performed Blast in the *Brassica* and *Brassica napus* Genome Resource database. Afterwards, we retrieved and inspected putative *COMT* sequences for the conserved SAM_MT_COMT (PS51588) motifs. The amino acid sequences of rapeseed *COMTs* were deduced from the putative open reading frames (ORFs) using DNAMAN software. The phylogenetic trees of COMT proteins from various species were constructed as previously described [27]. In brief, the predicted amino acid sequences of *COMTs* were aligned using the Vector NTI Advanced 11 program with the same multiple alignment parameters as previously utilized. The phylogenetic trees were constructed using the maximum likelihood (ML) algorithm implemented in the MEGA6.0 software with both deduced *COMT* full-length proteins and one of the *COMT* conserved domains, O-methyltransferase family 2 (IPR001077) sequences from *A*. *thaliana*, *B*. *napus*, *B*. *rapa* and *B*. *oleracea*. In addition, to study the interspecific relationship of intron I in three *Brassicaceae* plants and further explore the length diversification, intron I sequences of candidate *COMT1* genes were rooted using the ML algorithm.

The pairwise identity and similarity of proteins were calculated with MatGAT v2.02 (http://bitincka.com/ledion/matgat/). A domain analysis was performed using SMART (http://smart.embl-heidelberg.de/smart/set_mode.cgi?NORMAL=1) and other programs in UniProt (http://www.uniprot.org/) and ExPASy (http://www.expasy.org/). The numbers of introns in the *AtOMT1*, *BnCOMT1*, *BoCOMT1*, and *BrCOMT1* genes were determined by comparing genomic sequences to the cDNA sequences of their respective genes and displayed by an online resource, GSDS 2.0 (http://gsds.cbi.pku.edu.cn/).

### Klason lignin measurement

Middle stem parts of *B*. *napus* plants were destructively sampled for a Klason lignin analysis at mature stage in triplicate. Leaves were removed before air-drying. Dry stems were crushed in a grinder and sieved with a 60 mesh filter to achieve a uniform sample. Klason lignin was determined according to the protocol ‘Determination of Acid-Insoluble Lignin in Biomass’ released by the Department of Energy’s National Renewable Energy Laboratory in 1995 (accessible at infohouse.p2ric.org/ref/40/39182.pdf). Then, 500 mg of stem powder was digested by standard acid hydrolysis in 72% H_2_SO_4_ at 30°C for 2 h and in 4% H_2_SO_4_ at 121°C in an autoclave for 1 h. Acid-insoluble lignin was vacuum-filtered, dried and weighed. Total Klason lignin was determined by firing the solid for 3 h at 550°C and subtracting the resulting ash weight from the lignin weight. Grams of Klason lignin per gram of dry weight was calculated by dividing the total Klason lignin weight by the initial weight of dry matter used.

### Spatio-temporal expression of *BnCOMT1* genes in extreme lignin content *Brassica napus* lines

Four rapeseed plants were screened as lignin content extreme lines (two high-lignin content lines and two low-lignin content lines). Plants were randomly grown in greenhouse with artificial irrigation. For the drought treatment, we kept the soil moisture of treated plants at approximately 15% and at 25% for the control group. Young stems in four different growth stages (bud, early flowering, late flowering, and mature stages) were harvested after treatments, flash-frozen in liquid nitrogen and stored at –80°C. Three plants with the closest phenotype and growth status for each line were harvested, and harvesting was repeated three independent times.

qRT-PCR was performed as described above with slight modifications [27,28]. Total RNA samples were isolated from rapeseed tissues using the Plant RNAprep Pure Kit (Tiangen, Beijing). RNA was quantified on a NanoDrop 1000 (NanoDrop Technologies, Inc.), and RNA integrity was evaluated on a 1% agarose gel. RNA was transcribed into cDNA using a PrimeScript RT Reagent Kit (TaKaRa, Dalian). Primers used for qRT-PCR were designed using the Primer Premier 5.0 program to target the ORF of each gene with an amplicon sized between 80 and 250 bp (S1 Table). *Actin7* and *18S rRNA* served as reference genes. qRT-PCR was performed using 10-fold diluted cDNA and a Universal SYBR Green Supermix Kit (Bio-RAD, USA) on a CFX96 real-time PCR machine (Bio-Rad, USA). According to the MIQE Guidelines (Minimum Information for Publication of Quantitative Real-Time PCR Experiments) [29], the specificity of each primer pair was validated through regular PCR, 1.5% agarose gel electrophoresis and sequencing from both ends. We also performed primer tests with the CFX96 qPCR machine (Bio-Rad, USA) followed by a melting curve assessment, and the amplification efficiency (E) of each primer pair was calculated following a previously described protocol [27,30,31]. Three independent biological replicates were obtained, and the significance was determined with SPSS (p < 0.05).

### Tissue-specific expression characteristics of *BnCOMT1* genes

Wild type rapeseed (ZS11) plants were grown in a field with Chongqing soil mix (China) under natural conditions. Young root, stem, leaf, bud, flower and seeds of 15, 30 and 45 days after flowering (namely 15D, 30D, 45D) were harvested at different life stages. RNA isolation and qRT-PCR were performed as described above.

## Results

### *COMT1* gene identification in rapeseed

To understand the roles of the *COMT1* genes in rapeseed growth and development, we first identified the rapeseed *COMT1* genes. Because *Arabidopsi*s *thaliana* is a close relative to *B*. *napus*, we used *AtOMT1* (*At5g54160*) and 13 other *COMT-like* genes as queries in a BLAST search against the published *B*. *napus* genome resource and BRAD. As a result, we identified 42 *B*. *napus* CDSs representing *COMT* genes (S2 Table) with gene sequence identities higher than 85%. These CDSs were aligned to genomic DNA in the *B*. *napus* Genome Resource and were proofread to obtain high-confidence CDSs, which were then reciprocally BLAST searched against the *Arabidopsis* database (http://www.arabidopsis.org/Blast/index.jsp) to identify the putative orthologues in the model plant *Arabidopsis*. The *BnCOMT1* genes were annotated based on the *Arabidopsis* orthologues with *Bn* standing for *B*. *napus* genes. Afterwards, the amino acids of each CDS belonging to the *COMT* gene family 1 (*BnCOMT1*), which were homologues of *AtOMT1* with a function of lignin biosynthesis, were predicted using the DNAMAN or DNASTAR program. Thus, we successfully identified CDSs representing 12 *BnCOMT1* genes. We also identified 22 *B*. *rapa* CDSs and 20 *B*. *oleracea* CDSs using BRAD (S2 Table and S1 Fig)

**Fig 1:**
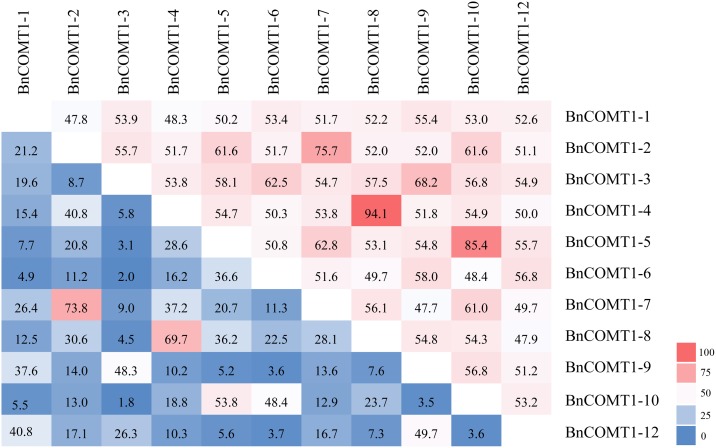
Sequence identity of intron I for *BnCOMT1s*. Data on the upper right present the sequence identity regardless of the unmatched regions possibly emerged in either end of two aligned intron sequences, and data on the bottom left present the sequence identity of full-length intron sequences.

Although the genome sequence of *B*. *napus* has been completed, the sequence assembly was not accurate. To facilitate subsequent phylogenetic and expression analyses, we designed primers based on the CDSs of the *BnCOMT1* genes to obtain the true ORFs and to verify the accuracy of genes available in the database. We succeeded in cloning the ORFs and genome sequences of five selected *BnCOMT1* genes. All five genes were highly consistent with their counterparts available on the database except that several regions showed differences in the degree of base-pairing. Other genomic DNA sequences of *BnCOMT1* genes were also downloaded from an online database (*Brassica napus* Genome Resource). All acquired *BnCOMT1* genes (except for *BnCOMT1-11*, which lacks exon I and part of intron I, and *BnCOMT1-7*, which merely retains the regions before intron II) were composed of four exons and three introns. Compared with the relatively conserved sequences for the exons and two other introns, dramatic diversity was observed for intron I with length ranging from 85 to 2742bp (S2 Fig). Fig 1 shows the details regarding intron I in eleven *BnCOMT1* genes (*BnCOMT1-11* lacks intron I) in the aspects of sequence identity. As a whole, *BnCOMT1-4* and *BnCOMT1-8* had the highest identity, followed by *BnCOMT1-5* and *BnCOMT1-10*, *BnCOMT1-2* and *BnCOMT1-7*.

**Fig 2:**
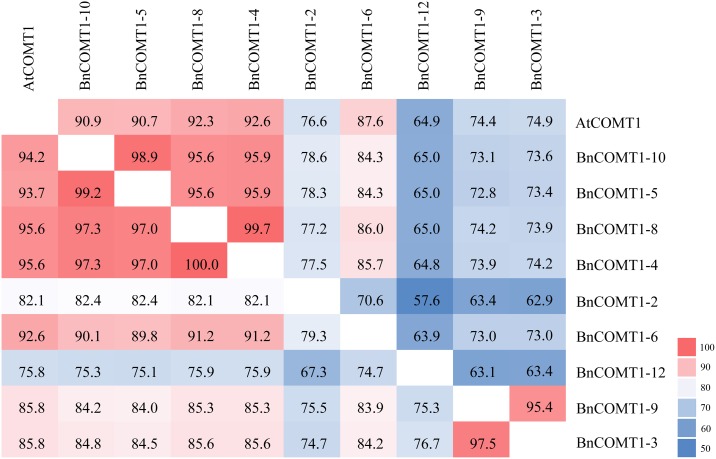
Protein sequence identity and similarity of BnCOMT1s. Data on the upper right present the sequence identity, and data on the bottom left present the sequence similarity.

**Fig 3:**
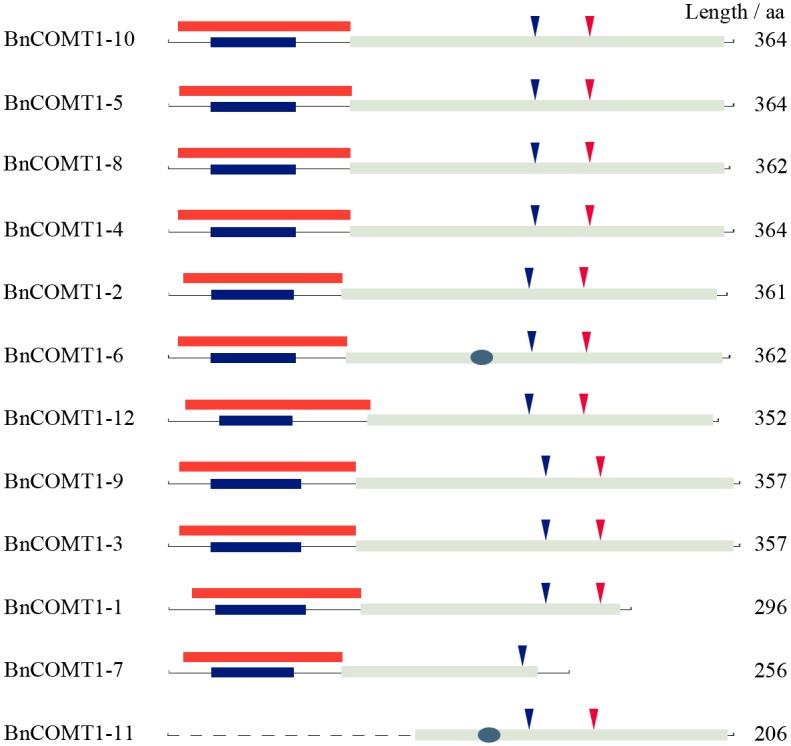
Domains and conserved sites prediction of BnCOMT1s. The red bars stand for winged helix-turn-helix DNA-binding domain (IPR011991), blue bars for plant methyltransferase dimerisation domain (IPR012967), grey bars for S-adenosyl-L-methionine-dependent methyltransferase domain (IPR029063). The blue circles present conserved site (IPR022657). The blue triangles stand for S-adenosyl-L-methionine binding sites, and red ones for proton acceptor active sites.

The deduced amino acid sequences of the 12 *BnCOMT1* genes were highly conserved with respect to size ranging from 352 to 367 aa regardless of three short genes (*BnCOMT1-1* in 296 aa, *BnCOMT1-7* in 256 aa, and *BnCOMT1-11* in 206 aa) (S3 Table). We also identified that the amino acid sequence identity of *BnCOMT1s* ranged from 57.6% to 99.7% (67.3–100% similarity, Fig 2), with highly conserved winged helix-turn-helix DNA-binding domain (IPR011991) (Fig 3), O-methyltransferase family 2 (IPR001077) belong to S-adenosyl-L-methionine-dependent methyltransferase domain (IPR029063), and Plant methyltransferase dimerisation domain (IPR012967) (Fig 3). We used the ScanProsite program (http://prosite.expasy.org/scanprosite/) and other programs to search for possible motifs that could be important for fulfilling the functions of the *BnCOMT1s*. The SAM_MT_COMT motif was identified in all 12 *BnCOMT1s*. As a methyltransferase, COMT proteins can bind S-adenosyl-L-methionine (SAM or AdoMet) as a substrate to generate S-adenosyl-L-homocysteine through the SAM_MT_COMT motif. As in AtOMT1, each of the 12 BnCOMT1 proteins contained a SAM binding site (Asp) and a proton accepter site (His) (Fig 3). One conserved site (IPR022657), namely Orn/DAP/Arg decarboxylase 2 was discovered in BnCOMT1-6 and BnCOMT1-11, respectively. The conserved site contains a stretch of three consecutive glycine residues and has been considered to be part of a substrate-binding region.

### Phylogenetic analysis of COMT proteins in *Brassicaceae*

**Fig 4:**
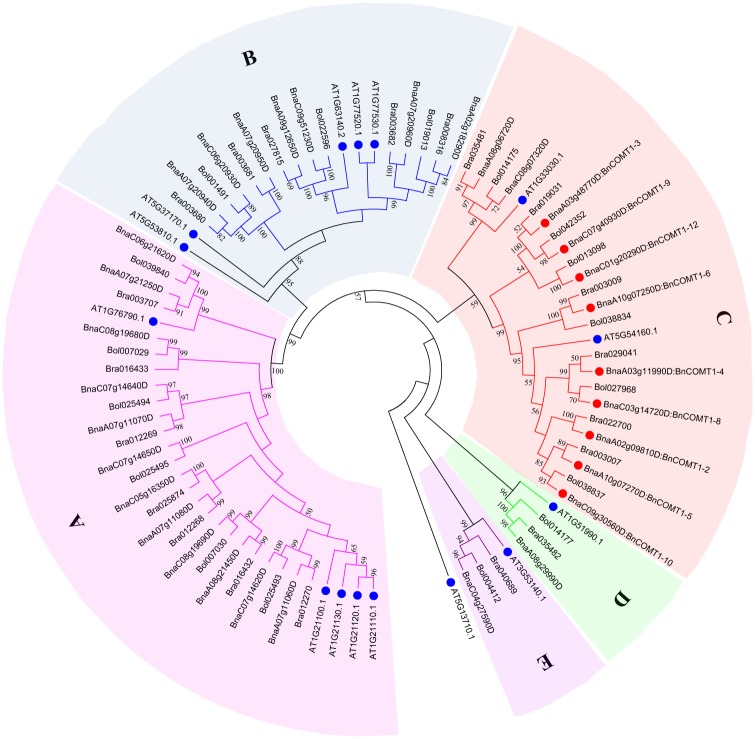
Phylogenetic trees for COMT proteins in *Brassica napus* and other plants. The tree was generated from an amino acid sequence alignment of 42BnCOMTs, 22BrCOMTs, 20BoCOMTs, 14AtCOMTs and another *Arabidopsis* protein with Methyltransferase_11 domain serving as outgroup species using MEGA 6.0 with the ClustalW program. The trees were constructed by ML methods (1000 bootstrap replicates). COMT proteins were clustered into five distinct groups (Groups A, B, C, D, and E). The tree is based on homologous groups showing evolutionary relationships with *COMTs*. Numbers next to the nodes are bootstrap values indicating frequencies of respective furcations found in 1000 replications of subset tree calculations. Only bootstrap values greater than 50% are denoted at the nodes.

**Fig 5:**
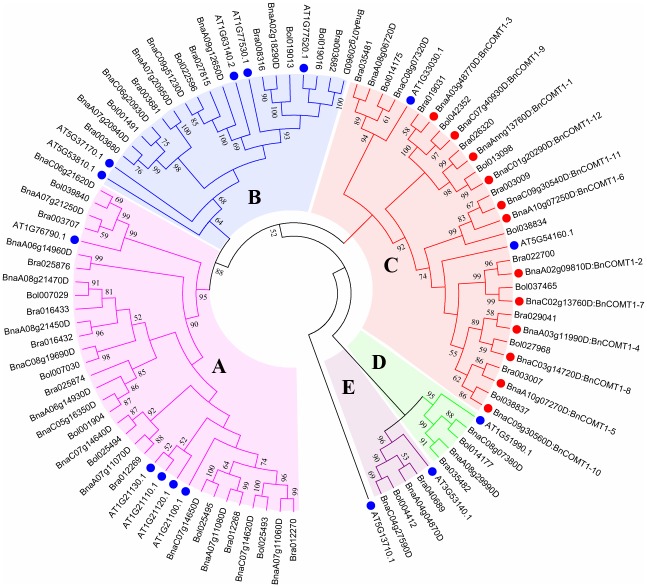
ML phylogenetic tree for Methyltransf_2 domains in COMT proteins. The phylogenetic tree derived by the ML method with bootstrap analysis (1000 replicates) from alignment of amino acid sequences of Methyltransferase domains predicted in COMT proteins from *Arabidopsis*, *B*. *rapa*, *B*. *oleracea*, *B*. *napus*, and outgroup using MEGA 6.0 program.

To better understand the evolutionary history of COMT families in *Brassicaceae*, we identified and performed a combined phylogenetic analysis of *Arabidopsis*, *B*. *rapa*, *B*. *oleracea*, *B*. *napus* COMT full-length proteins to obtain ML tree. Based on a TAIR classification, only one gene (*AT5G54160*) was identified as *OMT1*; others were considered *COMT*-like genes. Based on the similarity of the sequences and conserved motifs, the amino acids of 42 BnCOMT, 22 BrCOMT and 20 BoCOMT genes screened were identified from the *B*. *napus* genome resource and BRAD, and the selected sequences were used to construct the phylogenetic tree (Fig 4). Three short BnCOMT1s (BnCOMT1-1, BnCOMT1-7, BnCOMT1-11) were not included in this phylogenetic analysis. Furthermore, to compare the evolutional consistency of the specific regions and full-length COMT proteins, the conserved domain, Methyltransfer_2 ((IPR001077)) was also rooted by ML method (Fig 5). One *Arabidopsis* gene, *At5g13710* with a Methyltransferase type 11 (IPR013216) domain which is also classified into S-adenosyl-L-methionine-dependent methyltransferase domain (IPR029063), was added as an outgroup.

The data presented in Fig 4 suggested a divergent evolution for the *Brassicaceae OMTs*. Interestingly, the COMTs were clustered into five major groups. For functional annotation of the *AtOMTs*, Groups C, D and E represented the COMTs involved in the lignin biosynthetic process while Group A participated in glucosinolate metabolite pathways [32, 33]. Although we have no knowledge of whether the *At1g21110* and *At1g76790* genes also functioned in indole glucosinolate modification, it was confirmed that all five genes belong to family 2 of plant OMTs and are distantly related to other characterized plant OMTs which could also be seen in our phylogenetic analysis. Four *Arabidopsis OMTs* (*At1g21100*, *At1g21120*, *At1g21110*, *At1g21130*) were not clustered together with their *Brassica* counterparts, but instead, they aggregated and distinctly separated with other species. Similarly, this lack of corresponding *Arabidopsis* genes also appeared in Group B and Group C, that is, five of 12 *BnCOMT1s* were clustered together with *AtOMT1* (*At5g54160*), whereas another half showed no significant classification. Curiously, inconsistent with the trees rooted by full-length proteins, a difference emerged in the evolution order of methyltransferase_2 domains in those four *Arabidopsis OMT-like* genes (Fig 5). They seemed to evolve prior to several *Brassicaceae COMT*s in this domain-rooted tree, showing some evolutional asynchronicity between the domain regions and whole proteins. However, overall, the evolution of *COMT* genes was in step with their conserved domain since the clustering of most genes presented by the two trees showed relatively high identity.

Groups E and D predicted flavonoid O-methyltransferase and a putative O-diphenol-O-methyltransferase protein, which engage in monolignol biosynthesis and phenylpropanoid biosynthesis, respectively. Parallel to the phylogenetic relationship, BnCOMT1-4 and BnCOMT1-8, BnCOMT1-5 and BnCOMT1-10 showed higher amino acid similarities and identities (100 and 99.7%, 99.2 and 98.9%, respectively) than any other two BnCOMT1 proteins, and a closer relationship between *Arabidopsis OMT1* and these four *BnCOMT1*s could be further demonstrated by a higher similarity and identity in this study (Fig 2). Intriguingly, *BnCOMT1-2*, evolutionally closer to *Arabidopsis OMT1*, seemed non-conformed in terms of sequence identity and similarity, which presented much lower percentage compared with any of four genes above.

**Fig 6:**
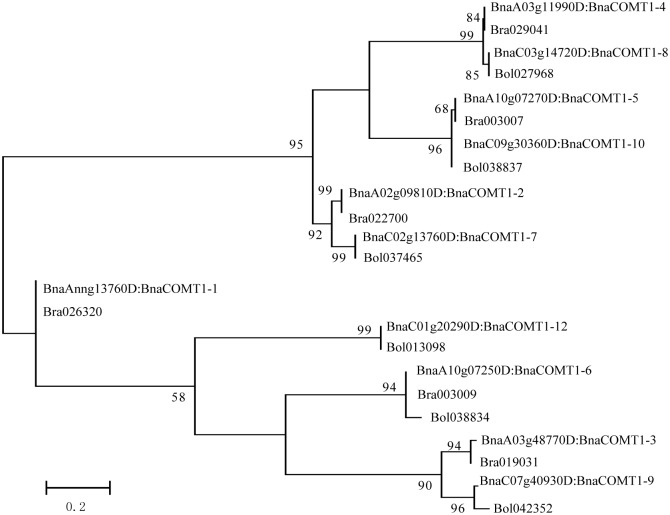
ML phylogenetic tree for intron I in Brassicaceae COMT1 genes. The phylogenetic tree derived by the ML method with bootstrap analysis (1000 replicates) from alignment of gene sequences of intron I from three *Brassicaceae* species using MEGA 6.0 program.

When we compared different phylogenetic tree rooted by full-length proteins, methyltransfer_2 domains and intron I sequences, we were surprised by the high consistency of *COMT1* genes in terms of evolutionary relationships. Moreover, intron I sequences of *BnCOMT1* genes were extremely conserved which were well congruent with their parent species (*B*. *rapa* and *B*. *oleracea*) with respect to not only their phylogenetic relationships but sequence identities even after subjecting to recombination and duplication (Fig 6 and S2 Fig). Most *BnCOMT1* intron I sequences were complete copies of that in their counterpart species, and some fragment insertions or deletions were only observed in *BnCOMT1-6*, *BnCOMT1-9* and *BnCOMT1-12*. For example, a tripled consecutive sequence ‘TCACCTTTCAATATTCTAACTTGTATATTTTATATATAATATGA’ in *Bol042352* deleted two repeats during recombination and triplication and left only one copy in *BnCOMT1-9*. Of course, the possibility that a duplication occurred after recombination to emerge *B*. *napus* should also be taken into account.

### Tissue-specific expression of *BnCOMT1* gene*s*

**Fig 7:**
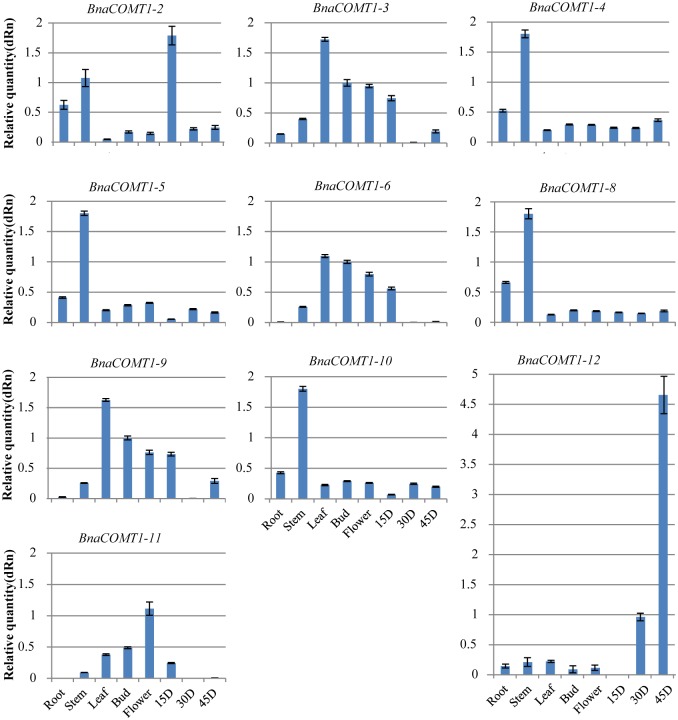
Tissue specific expression characteristics of *BnCOMT1s*. The tissue-specific expression levels of 10 of twelve *BnCOMT1* genes (except *BnCOMT1-1* and *BnCOMT1-7*) in eight different tissues (root, stem, leaf, bud, flower, 15D, 30D and 45D) were checked by real-time qRT-PCR.

To further explore the relationship between tissue specificity and phyletic evolution, *BnCOMT1* gene expression was detected using real-time fluorescent quantitative PCR (Fig 7 and S1 File). Although partial deletion was detected in *BnCOMT1-11*, it was included in our list of candidate *BnCOMT1* genes; thus, we wondered whether the sequence changes had any effects on transcription or gene function. We failed to design optimal primers for BnCOMT1-1 and BnCOMT1-7 because of large sequence similarity with other homologous genes, which resulted in a smaller region for primer design. In general, genes from the same lineage tended to present similar tissue specificity. The *BnCOMT1-4*, *BnCOMT1-5*, *BnCOMT1-8*, and *BnCOMT1-10* transcripts were expressed highest in the stem, with more than a threefold higher expression compared with that in other tissues. Moreover, similar to *BnCOMT1-4*, *BnCOMT1-5*, *BnCOMT1-8*, and *BnCOMT1-10*, *BnCOMT1-2* expression levels in the stem were higher than that in other tissues, except in 15D seed, which had nearly double the expression levels observed in the stem. Higher expression was detected in leaves for *BnCOMT1-3*, *BnCOMT1-9*, and *BnCOMT1-6* transcripts. Also amassed were *BnCOMT1-11* and *BnCOMT1-12* transcripts in flowers and 45D, respectively. Almost no transcript expression was observed in 30D seeds for *BnCOMT1-3*, *BnCOMT1-9*, and *BnCOMT1-6* while all three appeared in 45D seeds. We hypothesize that different *BnCOMT1*s might cooperate using complementary expression to stabilize the total lignin content in plants because of the differences in lignin precursor transformation in different tissues. In this manner, the gene functions are complementary and not redundant.

### Spatial expression of *BnCOMT1* gene*s* in the stem under drought stress

**Fig 8:**
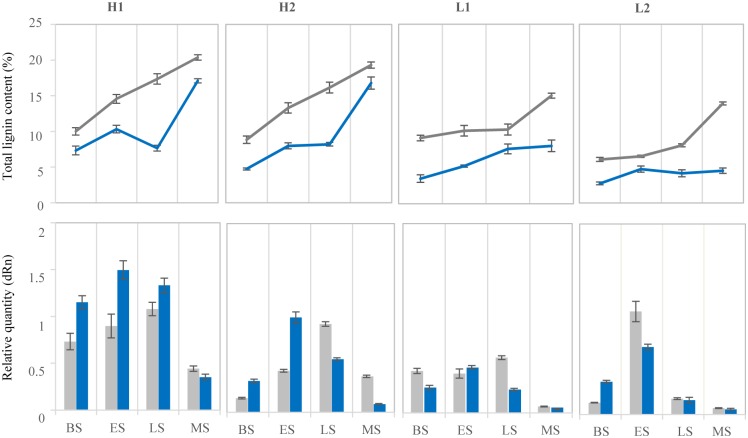
Total lignin content and spatial expression characteristics of stem specific *BnCOMT1s* under drought stress. H1, H2 were high-lignin content materials and L1, L2 were low materials. The grey color represents the drought treatment and blue represents the natural condition. BS, ES, LS, and MS were respectively short for budding stage, early flowering stage, late flowering stage and mature stage.

In addition to the tissue expressional specificity of *BnCOMT1s*, we were also curious about how they accumulated in plant growth processes. As we observed, the plant stem trend to be more rigidity under drought conditions, and similarly, plants with higher lignin content were more insusceptible to the external forces which could result in plant being broken off or flatten by. To illuminate the relationship between stem lignin content and the spatial expression of *BnCOMT1* genes and also, to investigate whether *BnCOMT1* genes are inducible in drought condition, quantitative RT-PCR was conducted to analyse the expression patterns of *BnCOMT1* genes in stems of four growth stages (bud, early flowering, late flowering and mature stages) and in four lignin content extreme lines (two relatively high lignin content lines and two low) under drought stress (Fig 8). To simplify the analysis, the transcripts accumulation level of four genes (*BnCOMT1-4*, *BnCOMT1-5*, *BnCOMT1-8*, and *BnCOMT1-10*) was detected as a whole anchored by a common primer in case these four genes showed a higher consistency in terms of tissue expressional specificity and expression abundance. To further guarantee the repeatability and experimental mutual authentication of each pair of lignin content extreme materials (H1 /H2 and L1/L2), we monitored a correlation analysis by IBM SSPS Statistics 22 (S2 File) in each group. The results showed that the accumulation of total lignin in H1 and H2 were highly positively correlated, reaching a level of 0.05 and 0.01 in drought treatment and natural conditions, respectively. Similarly, the drought-treated lignin contents of L1 and L2 also displayed a positive relationship with the significance of 0.011, which implied that the accumulation rhythms of total lignin between H1 and H2 (or L1 and L2) were consistent or somewhat close.

Fig 8 and S3 File showed the sustained accumulation of total lignin in extreme plants and lignin biosynthesis augmented under drought stress. In natural conditions, high-lignin plants accumulated their lignin content mainly in two separated periods, namely from budding to early flowering stage and late flowering to mature stage, respectively, blocked by an internal plateau. However, for low-lignin plants, this accumulation incident only occurred in early stages, then, remained constant until the mature stage. However, different to the control, in the first two stages from budding to blooming, the lignin contents of high-lignin plants accelerated in water deficit condition and saw a mild slow-down in the speed of accumulation in the following two stages. In contrast, the lignin contents of low-lignin plants were generally flat at a constant level in the first three stages before obviously accelerating in the mature stage. Since the stem lignin content measurement will induce irreversible destruction to the plants, we had to harvest the materials from different plants in the same line, which is why a fluctuation in the lignin accumulation graph was observed in H1 and L2 plant. In this aspect, some deviation of lignin accumulation in the same lines should take into account. Overall, drought stress augmented and accelerated the lignin accumulation in high-lignin plants but delayed it in low-lignin plants.

In natural conditions, the transcripts abundance of *BnCOMT1s* was globally proportional to the accumulation of total lignin since higher lignin content plants showed higher expression levels. (see Fig 8 and S4 File) Our data revealed that in rapeseed stem, *BnCOMT1s* expressed in all evaluated periods, and its level peaked in early flowering stage for natural status plants. However, under water deficit treatment, the peak was delayed to the late flowering stage (apart from L2, which also peaked in early flowering stage).

## Discussion

### Conservation of intron I in evolutionally close *BnCOMT1* genes indicating a potential impact of intron I in gene function

It is widely acknowledged that in addition to miRNA, siRNA, snoRNA and piRNA as well as various long ncRNA short molecules, non-coding RNAs (ncRNAs) are spectrum regulators of genomic processes within the nucleus and cytoplasm, which are indispensable for the proper organization and functioning of eukaryotic cells [34]. One of the pathways in which ncRNAs regulate gene expression is transcriptional gene silencing (TGS), which has been actively employed in plants [35]. Particularly, introns enriched with ncRNAs. A miRNAs contained in an intron of the *Arabidopsis* Dicer gene regulate the expression of its own gene [36]. Although no ncRNAs were predicted in intron I by the available online databases, we conducted a phylogenetic analysis of intron I from three *Brassica* species (*B*. *napus*, *B*. *rapa*, and *B*. *oleracea*) which showed that intron I of homologous *COMT1* genes in *B*. *napus* were well clustered with their counterparts in *B*. *rapa* and *B*. *oleracea*, and different *BnCOMT1* gene family members in the same chromosome were more identical. This conservation might indicate potential functions of *BnCOMT1* intron I. Moreover, our study of *BnCOMT1* intron I revealed that evolutionarily related *BnCOMT1* genes tended to share higher sequence identity in intron I(Fig 1 and S2 Fig). For example, intron I from *BnCOMT1-5* (A10) and *BnCOMT1-10* (C9) possessed 85.4% sequence identity, excluding a 1082 bp (1603 bp to 2684 bp region in *BnCOMT1-10*) deletion in *BnCOMT1-5*, and the gene sequence identity of *BnCOMT1-4* (A3) and *BnCOMT1-8* (C3) increased to 94.1% after excluding a 307 bp region that was radically different (a 412 to 718 bp region unique to *BnCOMT1-8*). Additionally, intron I of the adjacent homologous *BnCOMT1* genes on the same chromosome also tended to exhibit higher similarity. *BnCOMT1-5* (A10, 5734610–5737730) and *BnCOMT1-6* (A10, 5741474–5743071) had a higher sequence identity of 50.8% compared with that between other *BnCOMT1* genes on different chromosomes. *Brassica napus*, an amphidiploid of *Brassica oleracea* and *Brassica rapa*, is a complicated heterozygote possessing genomic triplication and complex rearrangements [37–40]. Although intron lengths varied considerably even between related species [41], intronic sequences of *BnCOMT1* genes were interspecifically conserved, and there was little impact on intron I during complex recombination and evolution. This phenomenon was also observed in *Gossypium*, whose intron size remained unchanged after being subjected to rounds of genome expansion and contraction [42].

In contrast, interspecific conserved intronic sequences showed extraordinary variation in the lengths of *BnCOMT1* intron I, ranging from 85 to 2742 bp (S2 Fig). Intron-specific selective constraints have been reserved during gene duplication and differ between introns within the same gene [43]. The largest variation among *BnCOMT1* introns was also observed. To date, studies related to plant intron length and gene structure have primarily demonstrated that genome size enlargements correlate with increases in the average intron length over a broad phylogenetic spectrum in small genomes [44,45], while introns expansion or contraction within a gene may be uncoupled from genome size and independent across species in conifers [46]. Moreover, a trend of increased length in the first introns in 5’ UTRs was also observed in a previous study [47], which is parallel to our results, while this tendency was not observed in some conifers [46]. Regardless of the potential impacts of intron length on genome structure, we were interested in the possible relationship between intronic variation and gene function. Transposable elements (TEs) attract great interest for their roles in plant genes. The abundance of TEs may be particularly high in long introns of repeat-rich genomes [46,48–50]. In this study, the repeat ‘TCACCTTTCAATATTCTAACTTGTATATTTTATATATAATATGA’ was tripled in intron I of *Bol042352* with its counterpart, *BnCOMT1-9*, conserving only one repeat. This bit of sequences may be a TE inserted into intron I and the insertion of transposons may be one reason for length variation. There is evidence that transposons are not just present to replicate themselves or to act a genomic destroyer that provides no benefit to the host. By regulating other genes, transposons may help plants respond and adapt to environmental stress which influences genetic and epigenetic regulation in plant genomes [51]. Although no more repeats were detected in other introns, the relationship of intron I length diversity and TE activity should be taken into consideration. Aside from transposable elements, selection pressure might be another fact that impacts intron length. To improve transcription efficiency or splicing accuracy, longer introns in regions of low recombination were selectively advantageous [52, 53]. To reduce selection pressure, some functionally important genes conserved longer introns or simply immobilized some TEs, given that large introns represent a higher cost of transcription [54]. Intron length declines following an increase in the expression level of corresponding genes in human [54]. However, from the evolutionary relationship of *BnCOMT1s*, longer introns generally diverged later than shorter ones, and no clear rule govern the relationship between intron size and expression level, which was inconsistent with the low-cost transcription hypothesis described in vertebrates but similar to genes in other plant, such as conifers. Another set of forces may exist driving the evolution of plant introns.

### Functional conservation and divergence in the evolution of *COMTs* in investigated *Brassicaceae* species

The phylogenetic tree topology of COMTs revealed considerable diversification and conservation in investigated *Brassicaceae* species. A comparative analysis indicated that *BnCOMT1-2*, *BnCOMT1-4*, *BnCOMT1-5*, *BnCOMT1-8*, and *BnCOMT1-10* were orthologous copies of *OMT1* genes in *Arabidopsis* while the other seven *Brassica napus COMT1* genes we cloned *in vivo* or *in silico* did not have orthologous copies in the *Arabidopsis* genome, suggesting that these genes were unique to the *Brassica* species. For the *OMT1* subclade, *BnCOMT1-2*, *BnCOMT1-4*, *BnCOMT1-5*, *BnCOMT1-8*, and *BnCOMT1-10* diverged and were reserved for functioning in lignin biosynthesis since their counterpart, *At5g54160*, mastered the indispensable position of *Arabidopsis COMT1s*, and other *BnCOMT1* genes are likely their paralogues. In case of the deletion of some *COMT1* paralogues in *Arabidopsis*, an anterior divergence first emerged between their ancestors, gene duplication then generated these paralogues in *Brassicaceae* after the *Brassica* lineages diverged from the *Arabidopsis* lineage. Likewise, the evolutionarily acceleration and duplication of the Brassicaceae genes could also be observed, which suggested that they were functionally active genes in the evolution process. However, the possibility that the corresponding genes were inactivated or deleted from the genome should also be considered [55]. The *COMT* genes identified in *B*. *rapa* and/or *B*. *oleracea* but not in *B*. *napus* might have been lost during evolution. Gene losses in *Brassica napus* have also been reported among *TT* (*transparent testa*) family genes [56,57]. Indeed, this type of gene loss could be from the accumulation of deleterious mutations, which are particularly prevalent soon after polyploidy events [58].

In general, the evolution of *COMT1* genes was relatively conserved among the three *Brassica* species. As *B*. *napus* was hybridized by *B*. *rapa* and *B*. *oleracea*, *BnCOMT1* genes maintained a high collinearity within each genome regarding their chromosomal locations (S2 Table). However, because of gene duplications and translocations [59] after hybridization, highly homogenous genes from *B*. *napus* (*BnCOMT1-9*) and *B*. *oleracea* (*Bol042352*) were located in distinct but neighbouring chromosomes (C07 and C06). In Group C, two orthologues, *BnaC08g07320D and Bol014175*, were located in C01 (subgenome) and C08 (subgenome), respectively (S2 Table).

In the comparison of multigene families, *COMT* genes from the same lineage tended to cluster into the same clade in the phylogenetic tree, suggesting that their divergence occurred prior to lineage duplication. The majority of *AtCOMTs* were involved in lignin biosynthesis, excluding *At1g21100*, *At1g21110*, *At1g21120*, *At1g21130* and *At1g76790*, which mainly participated in glucosinolate metabolism and other processes. Glucosinolates are natural components of many plants, including *Brassica* species, and contribute to the plant’s defence against biotic stress, in addition to promoting human health [60] via tryptophan or phenylalanine. The functions related to lignin biosynthesis diverged during evolution because the relevant *COMT* genes were involved not only in lignin biosynthesis but also in glucosinolate metabolism and melatonin synthesis suggesting functional divergence. Coincidently, lignin biosynthesis also begins with phenylalanine and tryptophan. A regulatory mechanism may be shared by two pathways to coordinate the distribution of resources and a competitive relationship cannot yet be excluded. The *COMT* genes that still play a role in lignin biosynthesis are crucial for the plant’s lignification. However, novel functions of *COMT* genes have extended the diversity of substrates and broadened the range of effects for *COMT* genes.

### Drought treatment research indicated the non-synchronization of total lignin deposition and *BnCOMT1s* transcripts accumulation in *B*. *napus*

Drought stress had a positive effect on lignin deposition and deepened the lignified degree of developing seedling stems in *Leucaena leucocephala* [61]. The expression of *CAD* (cinnamyl alcohol dehydrogenase) and several other genes involving in lignin biosynthesis increased during 48h to 72h under water stress in rice root [62]. However, this conclusion was not reached in all species. Subjected to drought stress, the amount of lignin decreased in the stem apical regions and showed an increased proportion of S/G in *E*. *urograndis* [63]. Similar results were obtained in maize, which showed that drought stress decreased the biosynthesis of lignin [64]. To date, limited reports are available on the effect of abiotic stresses such as drought in relation to lignin deposition in *B*. *napus*.

Generally, in our study, drought stress did not trigger a *BnCOMT1* expressional explosion but induced a relatively high accumulation of total lignin. That is to say, a lack of synchronization occurred between the accumulation processes of total lignin and *BnCOMT1* transcripts in most stages. Moreover, the total lignin accelerated its accumulation during the late flowering stage and mature stage even under the condition in which scarce amounts of *BnCOMT1s* were detected in L2 plants. Interestingly, higher *BnCOMT1* transcript accumulation of materials in the natural condition corresponded to lower total lignin deposition. Based on our observations, there may be an explanation for this contradiction. In high-lignin content samples, *BnCOMT1s* showed peak expression in the early flowering stage and decreased in the following developmental stages in the natural condition. Accompanied by this, limited deposition of total lignin was detected during the early flowering stage and late flowering stage. Furthermore, consistent *BnCOMT1* transcripts accumulation accompanied consistent total lignin deposition under drought stress from the budding stage to the late flowering stage. It seemed that the accumulation of total lignin was influenced by the variation rather than the absolute amount of *BnCOMT1* transcripts in *B*. *napus* stem. We hypothesize that the variation of *BnCOMT1s* played an elite role in the biosynthesis and accumulation of total lignin in the stem.

A previous study demonstrated that C*OMT1s* participated in monolignol biosynthesis of syringyl (S) units in various plants, including *Arabidopsis* [13], tobacco [65], poplar [66], maize [67], and alfalfa [11]. Since total lignin is mainly composed of three types of monolignols, namely S units, G units and a trace amount of H units in dicots, it is not difficult to speculate another possible reason for desynchrony of *BnCOMT1s* expression level and the total lignin content considering the likely effects on individual units. Another possibility is its participation in the melatonin biosynthesis pathway occupying part of *BnCOMT1*s transcripts. Generally, melatonin biosynthesis is catalysed from serotonin by two enzymes in plants, namely, serotonin N-acetyltransferase (SNAT) and N-acetylserotonin methyltransferase (ASMT) [68]. SNATs catalyse serotonin into N-acetylserotonin in chloroplasts, whereas ASMT is responsible for synthesizing melatonin from N-acetylserotonin, which occurs in the cytoplasm [69]. Due to the lack of ASMT homologues in many plants, COMT was thought to be a substitute enzyme responsible for the last step of melatonin biosynthesis in plants in early studies [16,70]. The catalysis of serotonin into 5-MT by AtCOMT can be achieved when serotonin is induced and accumulated to high levels in plant cells, also, COMT could O-methylate N-acetylserotonin to produce melatonin [15,71]. Interestingly, *in vitro* experiment showed that the major intermediate of melatonin biosynthesis varied depending on incubation temperatures. AtCOMT worked at low temperatures such as 37°C with 5-MT as major intermediate [15].

## Supporting Information

S1 Fig Gene structure of COMT genes. The gene structure schematic diagrams in ML phylogenetic tree were drawn by GSDS v2.0. Each exon is represented by a green box. Box length corresponds to exon length. The intermediate regions present intron length and intron phase. The domain positions were predicted by SMART with white box representing Dimersation domain, yellow for Methyltransf_2 domain and pink for Methyltransf_11.
(PDF)

S2 Fig Intron I sequences of *BnCOMT1* genes. (PDF)

S1 Table Primers for gene cloning and qRT-PCR. (DOCX)

S2 Table *COMT* gene accession numbers in *A*. *thaliana*, *B*. *napus*, *B*. *rapa*, *B*. *oleracea*. (DOCX)

S3 Table Main structural features of the *BnCOMT1* genes. (DOCX)

S1 File Data for tissue expression of *BnCOMT1* genes. (XLSX)

S2 File Correlation analysis of total lignin content in High/ Low lines. (XLSX)

S3 File Data for total lignin content measurement. (XLSX)

S4 File Data for spatio expression of stem specific *BnCOMT1*s. (XLSX)
