# Supplementary figures and images for: Cloning and Phylogenetic Analysis of Brassica napus L. Caffeic Acid O-Methyltransferase 1 Gene Family and Its Expression Pattern under Drought Stress
Source: PLoS One. 2016 Nov 10;11(11):e0165975. doi: 10.1371/journal.pone.0165975 (PMC5104432; doi:10.1371/journal.pone.0165975)

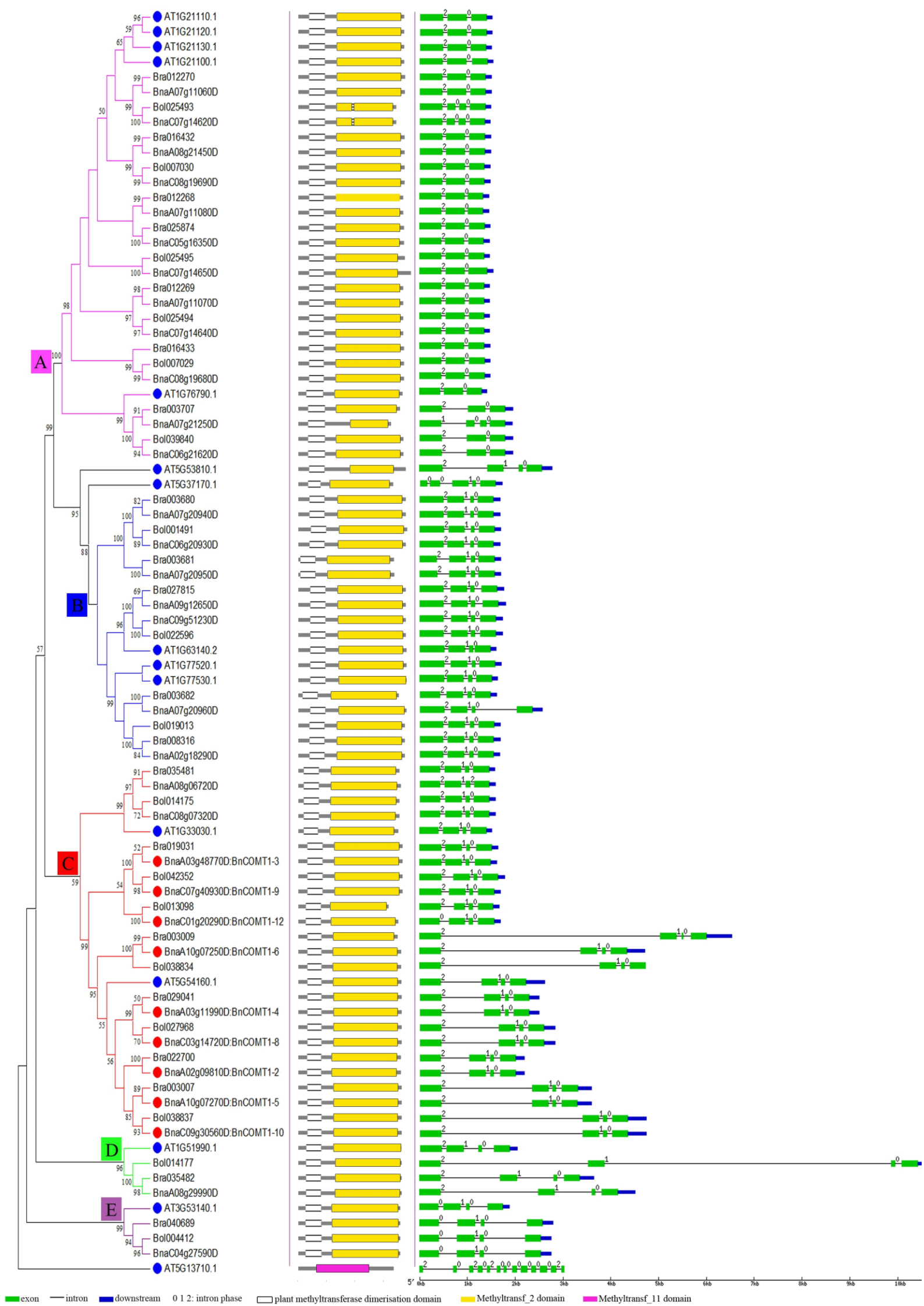

Supplement: S1 Fig — The gene structure schematic diagrams in ML phylogenetic tree were drawn by GSDS v2.0. Each exon is represented by a green box. Box length corresponds to exon length. The intermediate regions present intron length and intron phase. The domain positions were predicted by SMART with white box representing Dimersation domain, yellow for Methyltransf_2 domain and pink for Methyltransf_11. (PDF) [file pone.0165975.s001.pdf]
