# Supplementary material for: Cloning and Phylogenetic Analysis of Brassica napus L. Caffeic Acid O-Methyltransferase 1 Gene Family and Its Expression Pattern under Drought Stress
Source: PLoS One. 2016 Nov 10;11(11):e0165975. doi: 10.1371/journal.pone.0165975 (PMC5104432; doi:10.1371/journal.pone.0165975)
Supplement: S2 Fig — (PDF) [file pone.0165975.s002.pdf]

```
 BnaA10g07270D:BnaCOMT1-5     (1) --------------------------------------------------------------------------------
                Bra003007     (1) --------------------------------------------------------------------------------
BnaC09g30360D:BnaCOMT1-10     (1) --------------------------------------------------------------------------------
                Bol038837     (1) --------------------------------------------------------------------------------
 BnaA02g09810D:BnaCOMT1-2     (1) --------------------------------------------------------------------------------
                Bra022700     (1) --------------------------------------------------------------------------------
 BnaC02g13760D:BnaCOMT1-7     (1) --------------------------------------------------------------------------------
                Bol037465     (1) --------------------------------------------------------------------------------
 BnaA03g11990D:BnaCOMT1-4     (1) --------------------------------------------------------------------------------
                Bra029041     (1) --------------------------------------------------------------------------------
 BnaC03g14720D:BnaCOMT1-8     (1) --------------------------------------------------------------------------------
                Bol027968     (1) --------------------------------------------------------------------------------
 BnaA10g07250D:BnaCOMT1-6     (1) GTAAGTATTTACGAAGCCACACTGAAAATTATCATATCAGTTACTTTTTATAAGTTATGCTTCTTGGTTGATGTACTTAA
                Bra003009     (1) GTAAGTATTTACGAAGCCACACTGAAAATTATCATATCAGTTACTTTTTATAAGTTATGCTTCTTGGTTGATGTACTTAA
 BnaAnng13760D:BnaCOMT1-1     (1) --------------------------------------------------------------------------------
                Bra026320     (1) --------------------------------------------------------------------------------
 BnaC07g40930D:BnaCOMT1-9     (1) --------------------------------------------------------------------------------
                Bol042352     (1) --------------------------------------------------------------------------------
BnaC01g20290D:BnaCOMT1-12     (1) --------------------------------------------------------------------------------
                Bol013098     (1) --------------------------------------------------------------------------------
 BnaA03g48770D:BnaCOMT1-3     (1) --------------------------------------------------------------------------------
                Bra019031     (1) --------------------------------------------------------------------------------

 BnaA10g07270D:BnaCOMT1-5     (1) --------------------------------------------------------------------------------
                Bra003007     (1) --------------------------------------------------------------------------------
BnaC09g30360D:BnaCOMT1-10     (1) --------------------------------------------------------------------------------
                Bol038837     (1) --------------------------------------------------------------------------------
 BnaA02g09810D:BnaCOMT1-2     (1) --------------------------------------------------------------------------------
                Bra022700     (1) --------------------------------------------------------------------------------
 BnaC02g13760D:BnaCOMT1-7     (1) --------------------------------------------------------------------------------
                Bol037465     (1) --------------------------------------------------------------------------------
 BnaA03g11990D:BnaCOMT1-4     (1) --------------------------------------------------------------------------------
                Bra029041     (1) --------------------------------------------------------------------------------
 BnaC03g14720D:BnaCOMT1-8     (1) --------------------------------------------------------------------------------
                Bol027968     (1) --------------------------------------------------------------------------------
 BnaA10g07250D:BnaCOMT1-6    (81) TAATAATATACATAAGTGAGATCCACACAGTGCAAAGACCTTAATTTGTTACTGCACCAAAAGTATACCGGTCATATCAT
                Bra003009    (81) TAATAATATACATAAGTGAGATCCACACAGTGCAAAGACCTTAATTTGTTACTGCACCAAAAGTATACCGGTCATATCAT
 BnaAnng13760D:BnaCOMT1-1     (1) --------------------------------------------------------------------------------
                Bra026320     (1) --------------------------------------------------------------------------------
 BnaC07g40930D:BnaCOMT1-9     (1) --------------------------------------------------------------------------------
                Bol042352     (1) --------------------------------------------------------------------------------
BnaC01g20290D:BnaCOMT1-12     (1) --------------------------------------------------------------------------------
                Bol013098     (1) --------------------------------------------------------------------------------
 BnaA03g48770D:BnaCOMT1-3     (1) --------------------------------------------------------------------------------
                Bra019031     (1) --------------------------------------------------------------------------------

 BnaA10g07270D:BnaCOMT1-5     (1) --------------------------------------------------------------------------------
                Bra003007     (1) --------------------------------------------------------------------------------
BnaC09g30360D:BnaCOMT1-10     (1) --------------------------------------------------------------------------------
                Bol038837     (1) --------------------------------------------------------------------------------
 BnaA02g09810D:BnaCOMT1-2     (1) --------------------------------------------------------------------------------
                Bra022700     (1) --------------------------------------------------------------------------------
 BnaC02g13760D:BnaCOMT1-7     (1) --------------------------------------------------------------------------------
                Bol037465     (1) --------------------------------------------------------------------------------
 BnaA03g11990D:BnaCOMT1-4     (1) --------------------------------------------------------------------------------
                Bra029041     (1) --------------------------------------------------------------------------------
 BnaC03g14720D:BnaCOMT1-8     (1) --------------------------------------------------------------------------------
                Bol027968     (1) --------------------------------------------------------------------------------
 BnaA10g07250D:BnaCOMT1-6   (161) TGGTTACAACGATTAAACAGACTGGAGAACAAAATAATTTTAATAAATTATAGGAAAAA--------------AAATAGA
                Bra003009   (161) TGGTTACAACGATTAAACAGACTGGAGAACAAAATAATTTTAATAAATTATAGGAAAAATAATATATAAAAAAAAATAGA
 BnaAnng13760D:BnaCOMT1-1     (1) --------------------------------------------------------------------------------
                Bra026320     (1) --------------------------------------------------------------------------------
 BnaC07g40930D:BnaCOMT1-9     (1) --------------------------------------------------------------------------------
                Bol042352     (1) --------------------------------------------------------------------------------
BnaC01g20290D:BnaCOMT1-12     (1) --------------------------------------------------------------------------------
                Bol013098     (1) --------------------------------------------------------------------------------
 BnaA03g48770D:BnaCOMT1-3     (1) --------------------------------------------------------------------------------
                Bra019031     (1) --------------------------------------------------------------------------------

 BnaA10g07270D:BnaCOMT1-5     (1) --------------------------------------------------------------------------------
                Bra003007     (1) --------------------------------------------------------------------------------
BnaC09g30360D:BnaCOMT1-10     (1) --------------------------------------------------------------------------------
                Bol038837     (1) --------------------------------------------------------------------------------
 BnaA02g09810D:BnaCOMT1-2     (1) --------------------------------------------------------------------------------
                Bra022700     (1) --------------------------------------------------------------------------------
 BnaC02g13760D:BnaCOMT1-7     (1) --------------------------------------------------------------------------------
                Bol037465     (1) --------------------------------------------------------------------------------
 BnaA03g11990D:BnaCOMT1-4     (1) --------------------------------------------------------------------------------
                Bra029041     (1) --------------------------------------------------------------------------------
 BnaC03g14720D:BnaCOMT1-8     (1) --------------------------------------------------------------------------------
                Bol027968     (1) --------------------------------------------------------------------------------
 BnaA10g07250D:BnaCOMT1-6   (227) TAAAAAGGCATAAATTCAAAGTACGGGGTACTATGTTGGATTGGTCATCAATAAGATGGCCGACATCACTTGCTTTTAGA
                Bra003009   (241) TAAAAAGGCATAAATTCAAAGTACGGGGTACTATGTTGGATTGGTCATCAATAAGATGGCCGACATCACTTGCTTTTAGA
 BnaAnng13760D:BnaCOMT1-1     (1) --------------------------------------------------------------------------------
                Bra026320     (1) --------------------------------------------------------------------------------
 BnaC07g40930D:BnaCOMT1-9     (1) --------------------------------------------------------------------------------
                Bol042352     (1) --------------------------------------------------------------------------------
BnaC01g20290D:BnaCOMT1-12     (1) --------------------------------------------------------------------------------
                Bol013098     (1) --------------------------------------------------------------------------------
 BnaA03g48770D:BnaCOMT1-3     (1) --------------------------------------------------------------------------------
                Bra019031     (1) --------------------------------------------------------------------------------
```

```
  BnaA10g07270D:BnaCOMT1-5     (1) --------------------------------------------------------------------------------
                 Bra003007     (1) --------------------------------------------------------------------------------
 BnaC09g30360D:BnaCOMT1-10     (1) --------------------------------------------------------------------------------
                 Bol038837     (1) --------------------------------------------------------------------------------
  BnaA02g09810D:BnaCOMT1-2     (1) --------------------------------------------------------------------------------
                 Bra022700     (1) --------------------------------------------------------------------------------
  BnaC02g13760D:BnaCOMT1-7     (1) --------------------------------------------------------------------------------
                 Bol037465     (1) --------------------------------------------------------------------------------
  BnaA03g11990D:BnaCOMT1-4     (1) --------------------------------------------------------------------------------
                 Bra029041     (1) --------------------------------------------------------------------------------
  BnaC03g14720D:BnaCOMT1-8     (1) --------------------------------------------------------------------------------
                 Bol027968     (1) --------------------------------------------------------------------------------
  BnaA10g07250D:BnaCOMT1-6   (307) GCGTCCATTTATCGATCTAATATTACGTGTGGCCACAATCAAGATCAGCTTACGTTGTATAAAGAAAACACAATGGTCCC
                 Bra003009   (321) GCGTCCATTTATCGATCTAATATTACGTGTGGCCACAATCAAGATCAGCTTACGTTGTATAAAGAAAACACAATGGTCCC
  BnaAnng13760D:BnaCOMT1-1     (1) --------------------------------------------------------------------------------
                 Bra026320     (1) --------------------------------------------------------------------------------
  BnaC07g40930D:BnaCOMT1-9     (1) --------------------------------------------------------------------------------
                 Bol042352     (1) --------------------------------------------------------------------------------
 BnaC01g20290D:BnaCOMT1-12     (1) --------------------------------------------------------------------------------
                 Bol013098     (1) --------------------------------------------------------------------------------
  BnaA03g48770D:BnaCOMT1-3     (1) --------------------------------------------------------------------------------
                 Bra019031     (1) --------------------------------------------------------------------------------

  BnaA10g07270D:BnaCOMT1-5     (1) --------------------------------------------------------------------------------
                 Bra003007     (1) --------------------------------------------------------------------------------
 BnaC09g30360D:BnaCOMT1-10     (1) --------------------------------------------------------------------------------
                 Bol038837     (1) --------------------------------------------------------------------------------
  BnaA02g09810D:BnaCOMT1-2     (1) --------------------------------------------------------------------------------
                 Bra022700     (1) --------------------------------------------------------------------------------
  BnaC02g13760D:BnaCOMT1-7     (1) --------------------------------------------------------------------------------
                 Bol037465     (1) --------------------------------------------------------------------------------
  BnaA03g11990D:BnaCOMT1-4     (1) --------------------------------------------------------------------------------
                 Bra029041     (1) --------------------------------------------------------------------------------
  BnaC03g14720D:BnaCOMT1-8     (1) --------------------------------------------------------------------------------
                 Bol027968     (1) --------------------------------------------------------------------------------
  BnaA10g07250D:BnaCOMT1-6   (387) TCCGTTGTAAAGGATTTATCTAACTCTGTTCAAAAGAGTTACAAATCAGAAAGTAATACTATTTAAGTTTAAGAATTATA
                 Bra003009   (401) TCCGTTGTAAAGGATTTATCTAACTCTGTTCAAAAGAGTTACAAATCAGAAAGTAATACTATTTAAGTTTAAGAATTATA
  BnaAnng13760D:BnaCOMT1-1     (1) --------------------------------------------------------------------------------
                 Bra026320     (1) --------------------------------------------------------------------------------
  BnaC07g40930D:BnaCOMT1-9     (1) --------------------------------------------------------------------------------
                 Bol042352     (1) --------------------------------------------------------------------------------
 BnaC01g20290D:BnaCOMT1-12     (1) --------------------------------------------------------------------------------
                 Bol013098     (1) --------------------------------------------------------------------------------
  BnaA03g48770D:BnaCOMT1-3     (1) --------------------------------------------------------------------------------
                 Bra019031     (1) --------------------------------------------------------------------------------

  BnaA10g07270D:BnaCOMT1-5     (1) --------------------------------------------------------------------------------
                 Bra003007     (1) --------------------------------------------------------------------------------
 BnaC09g30360D:BnaCOMT1-10     (1) --------------------------------------------------------------------------------
                 Bol038837     (1) --------------------------------------------------------------------------------
  BnaA02g09810D:BnaCOMT1-2     (1) --------------------------------------------------------------------------------
                 Bra022700     (1) --------------------------------------------------------------------------------
  BnaC02g13760D:BnaCOMT1-7     (1) --------------------------------------------------------------------------------
                 Bol037465     (1) --------------------------------------------------------------------------------
  BnaA03g11990D:BnaCOMT1-4     (1) --------------------------------------------------------------------------------
                 Bra029041     (1) --------------------------------------------------------------------------------
  BnaC03g14720D:BnaCOMT1-8     (1) --------------------------------------------------------------------------------
                 Bol027968     (1) --------------------------------------------------------------------------------
  BnaA10g07250D:BnaCOMT1-6   (467) TTGAGATACAAATTGTGGCGAATGCATGCTGCTTTTTTTTACTGGGGGAAAACTAATAACCGACTATTTAGTTTAATACT
                 Bra003009   (481) TTGAGATACAAATTGTGGCGAATGCATGCTGCTTTTTTTTACTGGGGGAAAACTAATAACCGACTATTTAGTTTAATACT
  BnaAnng13760D:BnaCOMT1-1     (1) --------------------------------------------------------------------------------
                 Bra026320     (1) --------------------------------------------------------------------------------
  BnaC07g40930D:BnaCOMT1-9     (1) --------------------------------------------------------------------------------
                 Bol042352     (1) --------------------------------------------------------------------------------
 BnaC01g20290D:BnaCOMT1-12     (1) --------------------------------------------------------------------------------
                 Bol013098     (1) --------------------------------------------------------------------------------
  BnaA03g48770D:BnaCOMT1-3     (1) --------------------------------------------------------------------------------
                 Bra019031     (1) --------------------------------------------------------------------------------

  BnaA10g07270D:BnaCOMT1-5     (1) --------------------------------------------------------------------------------
                 Bra003007     (1) --------------------------------------------------------------------------------
 BnaC09g30360D:BnaCOMT1-10     (1) --------------------------------------------------------------------------------
                 Bol038837     (1) --------------------------------------------------------------------------------
  BnaA02g09810D:BnaCOMT1-2     (1) --------------------------------------------------------------------------------
                 Bra022700     (1) --------------------------------------------------------------------------------
  BnaC02g13760D:BnaCOMT1-7     (1) --------------------------------------------------------------------------------
                 Bol037465     (1) --------------------------------------------------------------------------------
  BnaA03g11990D:BnaCOMT1-4     (1) --------------------------------------------------------------------------------
                 Bra029041     (1) --------------------------------------------------------------------------------
  BnaC03g14720D:BnaCOMT1-8     (1) --------------------------------------------------------------------------------
                 Bol027968     (1) --------------------------------------------------------------------------------
  BnaA10g07250D:BnaCOMT1-6   (547) AACTAGATTTTGATCCGCGAGTTTGCGTAGATATATTTTTTTAAAAATATGATTCTATTTGTTTTTATGTCACTATTTAG
                 Bra003009   (561) AACTAGATTTTGATCCGCGAGTTCGCGTAGATATATTTTTTTAAAAATATGATTCTATTTGTTTTTATGTCACTATTTAG
  BnaAnng13760D:BnaCOMT1-1     (1) --------------------------------------------------------------------------------
                 Bra026320     (1) --------------------------------------------------------------------------------
  BnaC07g40930D:BnaCOMT1-9     (1) --------------------------------------------------------------------------------
                 Bol042352     (1) --------------------------------------------------------------------------------
 BnaC01g20290D:BnaCOMT1-12     (1) --------------------------------------------------------------------------------
                 Bol013098     (1) --------------------------------------------------------------------------------
  BnaA03g48770D:BnaCOMT1-3     (1) --------------------------------------------------------------------------------
                 Bra019031     (1) --------------------------------------------------------------------------------
```

BnaA10g07270D:BnaCOMT1-5 (1) --------------------------------------------------------------------------------
Bra003007 (1) --------------------------------------------------------------------------------
BnaC09g30360D:BnaCOMT1-10 (1) --------------------------------------------------------------------------------
Bol038837 (1) --------------------------------------------------------------------------------
BnaA02g09810D:BnaCOMT1-2 (1) --------------------------------------------------------------------------------
Bra022700 (1) --------------------------------------------------------------------------------
BnaC02g13760D:BnaCOMT1-7 (1) --------------------------------------------------------------------------------
Bol037465 (1) --------------------------------------------------------------------------------
BnaA03g11990D:BnaCOMT1-4 (1) --------------------------------------------------------------------------------
Bra029041 (1) --------------------------------------------------------------------------------
BnaC03g14720D:BnaCOMT1-8 (1) --------------------------------------------------------------------------------
Bol027968 (1) --------------------------------------------------------------------------------
BnaA10g07250D:BnaCOMT1-6 (627) GGTTGGGCAAAAACACTCGAATTCGAAGAACCGAACGGATCCCAATCCGAATAAATAGTACCAAATCCGAACCAAAATTG
Bra003009 (641) GGTTGGGCAAAAACACTCGAATTCGAAGAACCGAACGGATCCCAATCCGAATAAATAGTACCAAATCCGAACCAAAATTG
BnaAnng13760D:BnaCOMT1-1 (1) --------------------------------------------------------------------------------
Bra026320 (1) --------------------------------------------------------------------------------
BnaC07g40930D:BnaCOMT1-9 (1) --------------------------------------------------------------------------------
Bol042352 (1) --------------------------------------------------------------------------------
BnaC01g20290D:BnaCOMT1-12 (1) --------------------------------------------------------------------------------
Bol013098 (1) --------------------------------------------------------------------------------
BnaA03g48770D:BnaCOMT1-3 (1) --------------------------------------------------------------------------------
Bra019031 (1) --------------------------------------------------------------------------------

BnaA10g07270D:BnaCOMT1-5 (1) --------------------------------------------------------------------------------
Bra003007 (1) --------------------------------------------------------------------------------
BnaC09g30360D:BnaCOMT1-10 (1) --------------------------------------------------------------------------------
Bol038837 (1) --------------------------------------------------------------------------------
BnaA02g09810D:BnaCOMT1-2 (1) --------------------------------------------------------------------------------
Bra022700 (1) --------------------------------------------------------------------------------
BnaC02g13760D:BnaCOMT1-7 (1) --------------------------------------------------------------------------------
Bol037465 (1) --------------------------------------------------------------------------------
BnaA03g11990D:BnaCOMT1-4 (1) --------------------------------------------------------------------------------
Bra029041 (1) --------------------------------------------------------------------------------
BnaC03g14720D:BnaCOMT1-8 (1) --------------------------------------------------------------------------------
Bol027968 (1) --------------------------------------------------------------------------------
BnaA10g07250D:BnaCOMT1-6 (707) ATTAAATATCCAAATTATTCAAAATTTTGGTATTTGAAAAACTGAAACTTAATCTGATCCGAACCAGAGTATTTTGGGTA
Bra003009 (721) ATTAAATATCCAAATTATTCAAAATTTTGGTATTTGAAAAACTGAAACTTAATCTGATCCGAACC-----------GG-A
BnaAnng13760D:BnaCOMT1-1 (1) --------------------------------------------------------------------------------
Bra026320 (1) --------------------------------------------------------------------------------
BnaC07g40930D:BnaCOMT1-9 (1) --------------------------------------------------------------------------------
Bol042352 (1) --------------------------------------------------------------------------------
BnaC01g20290D:BnaCOMT1-12 (1) --------------------------------------------------------------------------------
Bol013098 (1) --------------------------------------------------------------------------------
BnaA03g48770D:BnaCOMT1-3 (1) --------------------------------------------------------------------------------
Bra019031 (1) --------------------------------------------------------------------------------

BnaA10g07270D:BnaCOMT1-5 (1) --------------------------------------------------------------------------------
Bra003007 (1) --------------------------------------------------------------------------------
BnaC09g30360D:BnaCOMT1-10 (1) --------------------------------------------------------------------------------
Bol038837 (1) --------------------------------------------------------------------------------
BnaA02g09810D:BnaCOMT1-2 (1) --------------------------------------------------------------------------------
Bra022700 (1) --------------------------------------------------------------------------------
BnaC02g13760D:BnaCOMT1-7 (1) --------------------------------------------------------------------------------
Bol037465 (1) --------------------------------------------------------------------------------
BnaA03g11990D:BnaCOMT1-4 (1) --------------------------------------------------------------------------------
Bra029041 (1) --------------------------------------------------------------------------------
BnaC03g14720D:BnaCOMT1-8 (1) --------------------------------------------------------------------------------
Bol027968 (1) --------------------------------------------------------------------------------
BnaA10g07250D:BnaCOMT1-6 (787) TCCAAAATAGATTTATATACTTATATAAATTAATTATTTTTAGATTTAATATTATATAAAAACATCTAGAATATATATGA
Bra003009 (788) --------------------------------------------------------------------------------
BnaAnng13760D:BnaCOMT1-1 (1) --------------------------------------------------------------------------------
Bra026320 (1) --------------------------------------------------------------------------------
BnaC07g40930D:BnaCOMT1-9 (1) --------------------------------------------------------------------------------
Bol042352 (1) --------------------------------------------------------------------------------
BnaC01g20290D:BnaCOMT1-12 (1) --------------------------------------------------------------------------------
Bol013098 (1) --------------------------------------------------------------------------------
BnaA03g48770D:BnaCOMT1-3 (1) --------------------------------------------------------------------------------
Bra019031 (1) --------------------------------------------------------------------------------

BnaA10g07270D:BnaCOMT1-5 (1) --------------------------------------------------------------------------------
Bra003007 (1) --------------------------------------------------------------------------------
BnaC09g30360D:BnaCOMT1-10 (1) --------------------------------------------------------------------------------
Bol038837 (1) --------------------------------------------------------------------------------
BnaA02g09810D:BnaCOMT1-2 (1) --------------------------------------------------------------------------------
Bra022700 (1) --------------------------------------------------------------------------------
BnaC02g13760D:BnaCOMT1-7 (1) --------------------------------------------------------------------------------
Bol037465 (1) --------------------------------------------------------------------------------
BnaA03g11990D:BnaCOMT1-4 (1) --------------------------------------------------------------------------------
Bra029041 (1) --------------------------------------------------------------------------------
BnaC03g14720D:BnaCOMT1-8 (1) --------------------------------------------------------------------------------
Bol027968 (1) --------------------------------------------------------------------------------
BnaA10g07250D:BnaCOMT1-6 (867) TACTTTTAAGTTTGTTTAAATACTTGAAAATATATACAAATAATCAAACGTAAATATCTAAATAGTTAAAATATACTCAA
Bra003009 (789) --------------------------------------------------------------------------------
BnaAnng13760D:BnaCOMT1-1 (1) --------------------------------------------------------------------------------
Bra026320 (1) --------------------------------------------------------------------------------
BnaC07g40930D:BnaCOMT1-9 (1) --------------------------------------------------------------------------------
Bol042352 (1) --------------------------------------------------------------------------------
BnaC01g20290D:BnaCOMT1-12 (1) --------------------------------------------------------------------------------
Bol013098 (1) --------------------------------------------------------------------------------
BnaA03g48770D:BnaCOMT1-3 (1) --------------------------------------------------------------------------------
Bra019031 (1) --------------------------------------------------------------------------------

```
 BnaA10g07270D:BnaCOMT1-5     (1) --------------------------------------------------------------------------------
                Bra003007     (1) --------------------------------------------------------------------------------
BnaC09g30360D:BnaCOMT1-10     (1) --------------------------------------------------------------------------------
                Bol038837     (1) --------------------------------------------------------------------------------
 BnaA02g09810D:BnaCOMT1-2     (1) --------------------------------------------------------------------------------
                Bra022700     (1) --------------------------------------------------------------------------------
 BnaC02g13760D:BnaCOMT1-7     (1) --------------------------------------------------------------------------------
                Bol037465     (1) --------------------------------------------------------------------------------
 BnaA03g11990D:BnaCOMT1-4     (1) --------------------------------------------------------------------------------
                Bra029041     (1) --------------------------------------------------------------------------------
 BnaC03g14720D:BnaCOMT1-8     (1) --------------------------------------------------------------------------------
                Bol027968     (1) --------------------------------------------------------------------------------
 BnaA10g07250D:BnaCOMT1-6   (947) AACTCCAAAACTACTTAAATAATTATTAATTATCTATCCAAATATTTAAACCAAACCAATTTATATGTTAAGTTAGGTAC
                Bra003009   (789) --CTCCAAAAATACTTAAATAATTATTAATTATCTATCCAAATATTTAAACCAAACCAATTTATATGTTAAGTTAGGTAC
 BnaAnng13760D:BnaCOMT1-1     (1) --------------------------------------------------------------------------------
                Bra026320     (1) --------------------------------------------------------------------------------
 BnaC07g40930D:BnaCOMT1-9     (1) --------------------------------------------------------------------------------
                Bol042352     (1) --------------------------------------------------------------------------------
BnaC01g20290D:BnaCOMT1-12     (1) --------------------------------------------------------------------------------
                Bol013098     (1) --------------------------------------------------------------------------------
 BnaA03g48770D:BnaCOMT1-3     (1) --------------------------------------------------------------------------------
                Bra019031     (1) --------------------------------------------------------------------------------

 BnaA10g07270D:BnaCOMT1-5     (1) --------------------------------------------------------------------------------
                Bra003007     (1) --------------------------------------------------------------------------------
BnaC09g30360D:BnaCOMT1-10     (1) --------------------------------------------------------------------------------
                Bol038837     (1) --------------------------------------------------------------------------------
 BnaA02g09810D:BnaCOMT1-2     (1) --------------------------------------------------------------------------------
                Bra022700     (1) --------------------------------------------------------------------------------
 BnaC02g13760D:BnaCOMT1-7     (1) --------------------------------------------------------------------------------
                Bol037465     (1) --------------------------------------------------------------------------------
 BnaA03g11990D:BnaCOMT1-4     (1) --------------------------------------------------------------------------------
                Bra029041     (1) --------------------------------------------------------------------------------
 BnaC03g14720D:BnaCOMT1-8     (1) --------------------------------------------------------------------------------
                Bol027968     (1) --------------------------------------------------------------------------------
 BnaA10g07250D:BnaCOMT1-6  (1027) TCTAACATATGTTATCTAAATTTATATGTAGTATATTCTATTGTTTATAGTTTTTTTTTTAAATTAAATAATAAAATAAA
                Bra003009   (867) TCTAACATATGTTATCTAAATTTATATGTAGTATATTCTATTTTTTATAGATTTTTTTTAAAATTAAATAATAAAATAAA
 BnaAnng13760D:BnaCOMT1-1     (1) --------------------------------------------------------------------------------
                Bra026320     (1) --------------------------------------------------------------------------------
 BnaC07g40930D:BnaCOMT1-9     (1) --------------------------------------------------------------------------------
                Bol042352     (1) --------------------------------------------------------------------------------
BnaC01g20290D:BnaCOMT1-12     (1) --------------------------------------------------------------------------------
                Bol013098     (1) --------------------------------------------------------------------------------
 BnaA03g48770D:BnaCOMT1-3     (1) --------------------------------------------------------------------------------
                Bra019031     (1) --------------------------------------------------------------------------------

 BnaA10g07270D:BnaCOMT1-5     (1) --------------------------------------------------------------------------------
                Bra003007     (1) --------------------------------------------------------------------------------
BnaC09g30360D:BnaCOMT1-10     (1) --------------------------------------------------------------------------------
                Bol038837     (1) --------------------------------------------------------------------------------
 BnaA02g09810D:BnaCOMT1-2     (1) --------------------------------------------------------------------------------
                Bra022700     (1) --------------------------------------------------------------------------------
 BnaC02g13760D:BnaCOMT1-7     (1) --------------------------------------------------------------------------------
                Bol037465     (1) --------------------------------------------------------------------------------
 BnaA03g11990D:BnaCOMT1-4     (1) --------------------------------------------------------------------------------
                Bra029041     (1) --------------------------------------------------------------------------------
 BnaC03g14720D:BnaCOMT1-8     (1) --------------------------------------------------------------------------------
                Bol027968     (1) --------------------------------------------------------------------------------
 BnaA10g07250D:BnaCOMT1-6  (1107) TTTTAAAATAATTTAAATTGGTTATCCAAATCCAAACTGAATCCTCAAAGATTTGAACTAAACACGAAATCCCAAACAAA
                Bra003009   (947) TTTTAAAATAATTTAAATTGGTTATCCAAATCCAAACTGAATCCTCAAAGATTTGAACTAAACACGAAATCCCAAACAAA
 BnaAnng13760D:BnaCOMT1-1     (1) --------------------------------------------------------------------------------
                Bra026320     (1) --------------------------------------------------------------------------------
 BnaC07g40930D:BnaCOMT1-9     (1) --------------------------------------------------------------------------------
                Bol042352     (1) --------------------------------------------------------------------------------
BnaC01g20290D:BnaCOMT1-12     (1) --------------------------------------------------------------------------------
                Bol013098     (1) --------------------------------------------------------------------------------
 BnaA03g48770D:BnaCOMT1-3     (1) --------------------------------------------------------------------------------
                Bra019031     (1) --------------------------------------------------------------------------------

 BnaA10g07270D:BnaCOMT1-5     (1) --------------------------------------------------------------------------------
                Bra003007     (1) --------------------------------------------------------------------------------
BnaC09g30360D:BnaCOMT1-10     (1) --------------------------------------------------------------------------------
                Bol038837     (1) --------------------------------------------------------------------------------
 BnaA02g09810D:BnaCOMT1-2     (1) --------------------------------------------------------------------------------
                Bra022700     (1) --------------------------------------------------------------------------------
 BnaC02g13760D:BnaCOMT1-7     (1) --------------------------------------------------------------------------------
                Bol037465     (1) --------------------------------------------------------------------------------
 BnaA03g11990D:BnaCOMT1-4     (1) --------------------------------------------------------------------------------
                Bra029041     (1) --------------------------------------------------------------------------------
 BnaC03g14720D:BnaCOMT1-8     (1) --------------------------------------------------------------------------------
                Bol027968     (1) --------------------------------------------------------------------------------
 BnaA10g07250D:BnaCOMT1-6  (1187) CCCCGAAAACGAACCCGAACGCCCACCCCTAATCACTATTATATATCTCATATGTGTCATCATAGGTTACAAAAATATGT
                Bra003009  (1027) CCCCGAAAACGAACCCGAACGCCCACCCCTAATCACTATTATATATCTCATATGTGTCATCATAGGTTACAAAAATATGT
 BnaAnng13760D:BnaCOMT1-1     (1) --------------------------------------------------------------------------------
                Bra026320     (1) --------------------------------------------------------------------------------
 BnaC07g40930D:BnaCOMT1-9     (1) --------------------------------------------------------------------------------
                Bol042352     (1) --------------------------------------------------------------------------------
BnaC01g20290D:BnaCOMT1-12     (1) --------------------------------------------------------------------------------
                Bol013098     (1) --------------------------------------------------------------------------------
 BnaA03g48770D:BnaCOMT1-3     (1) --------------------------------------------------------------------------------
                Bra019031     (1) --------------------------------------------------------------------------------
```

```
 BnaA10g07270D:BnaCOMT1-5     (1) --------------------------------------------------------------------------------
                Bra003007     (1) --------------------------------------------------------------------------------
BnaC09g30360D:BnaCOMT1-10     (1) --------------------------------------------------------------------------------
                Bol038837     (1) --------------------------------------------------------------------------------
 BnaA02g09810D:BnaCOMT1-2     (1) --------------------------------------------------------------------------------
                Bra022700     (1) --------------------------------------------------------------------------------
 BnaC02g13760D:BnaCOMT1-7     (1) --------------------------------------------------------------------------------
                Bol037465     (1) --------------------------------------------------------------------------------
 BnaA03g11990D:BnaCOMT1-4     (1) --------------------------------------------------------------------------------
                Bra029041     (1) --------------------------------------------------------------------------------
 BnaC03g14720D:BnaCOMT1-8     (1) --------------------------------------------------------------------------------
                Bol027968     (1) --------------------------------------------------------------------------------
 BnaA10g07250D:BnaCOMT1-6  (1267) GTTATCATATAATTAATCGTATTATATATGTACCATCAAATAGGTAATCTTATAATTAATAATATTTTATATGTACAATC
                Bra003009  (1107) GTTATCATATAATTAATCGTATTATATATGTACCATTAAATAAGTAATCTTATAATTAATAATATTTTATATGTACAATC
 BnaAnng13760D:BnaCOMT1-1     (1) --------------------------------------------------------------------------------
                Bra026320     (1) --------------------------------------------------------------------------------
 BnaC07g40930D:BnaCOMT1-9     (1) --------------------------------------------------------------------------------
                Bol042352     (1) --------------------------------------------------------------------------------
BnaC01g20290D:BnaCOMT1-12     (1) --------------------------------------------------------------------------------
                Bol013098     (1) --------------------------------------------------------------------------------
 BnaA03g48770D:BnaCOMT1-3     (1) --------------------------------------------------------------------------------
                Bra019031     (1) --------------------------------------------------------------------------------

 BnaA10g07270D:BnaCOMT1-5     (1) --------------------------------------------------------------------------------
                Bra003007     (1) --------------------------------------------------------------------------------
BnaC09g30360D:BnaCOMT1-10     (1) --------------------------------------------------------------------------------
                Bol038837     (1) --------------------------------------------------------------------------------
 BnaA02g09810D:BnaCOMT1-2     (1) --------------------------------------------------------------------------------
                Bra022700     (1) --------------------------------------------------------------------------------
 BnaC02g13760D:BnaCOMT1-7     (1) --------------------------------------------------------------------------------
                Bol037465     (1) --------------------------------------------------------------------------------
 BnaA03g11990D:BnaCOMT1-4     (1) --------------------------------------------------------------------------------
                Bra029041     (1) --------------------------------------------------------------------------------
 BnaC03g14720D:BnaCOMT1-8     (1) --------------------------------------------------------------------------------
                Bol027968     (1) --------------------------------------------------------------------------------
 BnaA10g07250D:BnaCOMT1-6  (1347) ATATAAATGATCACATATATTATATTTTTAAATTCCAATATGAAATATAAAAACCATAATTTAAGTTGATGTTTGAAATT
                Bra003009  (1187) ATATAAATGATCACATATATTATATTTTTAAATTCCAATATGAAATATAAAAACCATAATTTAAGTTGATGTTTGAAATT
 BnaAnng13760D:BnaCOMT1-1     (1) --------------------------------------------------------------------------------
                Bra026320     (1) --------------------------------------------------------------------------------
 BnaC07g40930D:BnaCOMT1-9     (1) --------------------------------------------------------------------------------
                Bol042352     (1) --------------------------------------------------------------------------------
BnaC01g20290D:BnaCOMT1-12     (1) --------------------------------------------------------------------------------
                Bol013098     (1) --------------------------------------------------------------------------------
 BnaA03g48770D:BnaCOMT1-3     (1) --------------------------------------------------------------------------------
                Bra019031     (1) --------------------------------------------------------------------------------

 BnaA10g07270D:BnaCOMT1-5     (1) --------------------------------------------------------------------------------
                Bra003007     (1) --------------------------------------------------------------------------------
BnaC09g30360D:BnaCOMT1-10     (1) --------------------------------------------------------------------------------
                Bol038837     (1) --------------------------------------------------------------------------------
 BnaA02g09810D:BnaCOMT1-2     (1) --------------------------------------------------------------------------------
                Bra022700     (1) --------------------------------------------------------------------------------
 BnaC02g13760D:BnaCOMT1-7     (1) --------------------------------------------------------------------------------
                Bol037465     (1) --------------------------------------------------------------------------------
 BnaA03g11990D:BnaCOMT1-4     (1) --------------------------------------------------------------------------------
                Bra029041     (1) --------------------------------------------------------------------------------
 BnaC03g14720D:BnaCOMT1-8     (1) --------------------------------------------------------------------------------
                Bol027968     (1) --------------------------------------------------------------------------------
 BnaA10g07250D:BnaCOMT1-6  (1427) GGGCTTTGTATTGTAATTTTCTTATATATATTGAAAACATTTTTATAATGGTTATTGGAAAATATGTTAGTAAAAATCAA
                Bra003009  (1267) GGGCTTTGTATTGTAATTTTCTTATATATATTGAAAACATTTTTATAATGGTTATTAGAAAATATGTTAGTAAAAATCAA
 BnaAnng13760D:BnaCOMT1-1     (1) --------------------------------------------------------------------------------
                Bra026320     (1) --------------------------------------------------------------------------------
 BnaC07g40930D:BnaCOMT1-9     (1) --------------------------------------------------------------------------------
                Bol042352     (1) --------------------------------------------------------------------------------
BnaC01g20290D:BnaCOMT1-12     (1) --------------------------------------------------------------------------------
                Bol013098     (1) --------------------------------------------------------------------------------
 BnaA03g48770D:BnaCOMT1-3     (1) --------------------------------------------------------------------------------
                Bra019031     (1) --------------------------------------------------------------------------------

 BnaA10g07270D:BnaCOMT1-5     (1) --------------------------------------------------------------------------------
                Bra003007     (1) --------------------------------------------------------------------------------
BnaC09g30360D:BnaCOMT1-10     (1) --------------------------------------------------------------------------------
                Bol038837     (1) --------------------------------------------------------------------------------
 BnaA02g09810D:BnaCOMT1-2     (1) --------------------------------------------------------------------------------
                Bra022700     (1) --------------------------------------------------------------------------------
 BnaC02g13760D:BnaCOMT1-7     (1) --------------------------------------------------------------------------------
                Bol037465     (1) --------------------------------------------------------------------------------
 BnaA03g11990D:BnaCOMT1-4     (1) --------------------------------------------------------------------------------
                Bra029041     (1) --------------------------------------------------------------------------------
 BnaC03g14720D:BnaCOMT1-8     (1) --------------------------------------------------------------------------------
                Bol027968     (1) --------------------------------------------------------------------------------
 BnaA10g07250D:BnaCOMT1-6  (1507) TTTTTGAATATATGTATATTTTTGACTGAATTTTTGATATAAATAAATTTAAATTATTATTTTGATTTGAATATATATAT
                Bra003009  (1347) TTTTTGAATATATGTATATTTTTGACTGAATTTTTGATATAAATAAATTTAAATTATTATTTTGATTTGAATATATATAT
 BnaAnng13760D:BnaCOMT1-1     (1) --------------------------------------------------------------------------------
                Bra026320     (1) --------------------------------------------------------------------------------
 BnaC07g40930D:BnaCOMT1-9     (1) --------------------------------------------------------------------------------
                Bol042352     (1) --------------------------------------------------------------------------------
BnaC01g20290D:BnaCOMT1-12     (1) --------------------------------------------------------------------------------
                Bol013098     (1) --------------------------------------------------------------------------------
 BnaA03g48770D:BnaCOMT1-3     (1) --------------------------------------------------------------------------------
                Bra019031     (1) --------------------------------------------------------------------------------
```

```
 BnaA10g07270D:BnaCOMT1-5     (1) -----------------------------------------------------------------------GTAAGTA-G
               Bra003007      (1) -----------------------------------------------------------------------GTAAGTA-G
BnaC09g30360D:BnaCOMT1-10     (1) -----------------------------------------------------------------------GTAAGTA-G
               Bol038837      (1) -----------------------------------------------------------------------GTAAGTA-G
 BnaA02g09810D:BnaCOMT1-2     (1) ---------------------------------------------------------------------------GTAAG
               Bra022700      (1) ---------------------------------------------------------------------------GTAAG
 BnaC02g13760D:BnaCOMT1-7     (1) ---------------------------------------------------------------------------GTACG
               Bol037465      (1) ---------------------------------------------------------------------------GTACG
 BnaA03g11990D:BnaCOMT1-4     (1) ---------------------------------------------------------------------------GTAAG
               Bra029041      (1) ---------------------------------------------------------------------------GTAAG
 BnaC03g14720D:BnaCOMT1-8     (1) ---------------------------------------------------------------------------GTAAG
               Bol027968      (1) ---------------------------------------------------------------------------GTAAG
 BnaA10g07250D:BnaCOMT1-6  (1587) CAAGTAACATAAGCCCATTACTTTTTAATATAATGTAATGAACTTCTAATTTTATTAATAGCATAAGTCCATTATGTTTT
               Bra003009   (1427) CAAGTAACATAAGCCCATTACTTTTTAATATAATGTAATGAACTTCTAATTTTATTAATAGCATAAGTCCATTATGTTTT
 BnaAnng13760D:BnaCOMT1-1     (1) --------------------------------------------------------------------------------
               Bra026320      (1) --------------------------------------------------------------------------------
 BnaC07g40930D:BnaCOMT1-9     (1) --------------------------------------------------------------------------------
               Bol042352      (1) --------------------------------------------------------------------------------
BnaC01g20290D:BnaCOMT1-12     (1) --------------------------------------------------------------------------------
               Bol013098      (1) --------------------------------------------------------------------------------
 BnaA03g48770D:BnaCOMT1-3     (1) --------------------------------------------------------------------------------
               Bra019031      (1) --------------------------------------------------------------------------------

 BnaA10g07270D:BnaCOMT1-5     (9) TATTTC--TATATCACCAAGGAGAATGCTCATATTATTTTCAAAGATCTCGTGCTAATTCTAGTTAGAAAAACTC--TTC
               Bra003007      (9) TATTTC--TATATCACCAAGGAGAGTACTCATATTATTTTCAAAGATCTCGTGCTAATTCTAGTTAGAAAAACTC--TTC
BnaC09g30360D:BnaCOMT1-10     (9) TATTTC--TATATCACCAAGGAGAGTACTCATATTATTTTCATAGATCTCGTGCTAATTCTAGTTAGAAAAACTC--TTC
               Bol038837      (9) TATTTC--TATATCACCAAGGAGAGTACTCATATTATTTTCATAGATCTCGTGCTAATTCTAGTTAGAAAAACTC--TTC
 BnaA02g09810D:BnaCOMT1-2     (6) TATTTT--GATATTATCAA-AATTATTATCATATGATTTCAAAACAGCTTGTGCCAAACCAAGTTAGAAAATCTA--TTC
               Bra022700      (6) TATTTT--GATATTATCAA-AATTATTATCATATGATTTCAAAACAGCTTGTGCCAAACCAAGTTAGAAAATCTA--TTC
 BnaC02g13760D:BnaCOMT1-7     (6) TATTTT--GATATTATCAAGAATTATTATCATATGATTTCAAAACATCTTGTGCCAACCCATGTTAGAAAAACTA--TTC
               Bol037465      (6) TATTTT--GATGTTATCAAGAATTATTATCATATGATTTCAAAACATCTTGTGCCAACCCATGTTAGAAAAACTA--TTC
 BnaA03g11990D:BnaCOMT1-4     (6) TTTTTTACGATATAACCATG----A----CATCTTTATTTGATGGATTTTGTGCTATATCAAGTTAGAAAAAAAAAAAAG
               Bra029041      (6) TTTTTTACGATATAACCATG----A----CATCTTTATTTGATGGATTTTGTGCTATATCAAGTTAGAAAAAAAAAAAAG
 BnaC03g14720D:BnaCOMT1-8     (6) TTTTTTTCGATATTACCAAG----A----CATCTTTATTGGATAGATCTTGTGCTATATCAAGTTAGAAAAAAAAGCTTC
               Bol027968      (6) TTTTTTTCGATATTACCAAG----A----CATCTTTATTGGATAGATCTTGTGCTATATCAAGTTAGAAAAAAAAGCTTC
 BnaA10g07250D:BnaCOMT1-6  (1667) TTTTTTCCAATTTACTGCTATTAATATTTCCAAACAACACTATACTTTTTTAACTACTATCTATGTTGCCAAACAAAAGC
               Bra003009   (1507) TTTTTTCTAATTTACTGCTATTAATATTTCCAAACAACACTATACTTTTTTAACTACTATCTATGTTGCCAAACAAAAGC
 BnaAnng13760D:BnaCOMT1-1     (1) --------------------------------------------------------------------------------
               Bra026320      (1) --------------------------------------------------------------------------------
 BnaC07g40930D:BnaCOMT1-9     (1) --------------------------------------------------------------------------------
               Bol042352      (1) --------------------------------------------------------------------------------
BnaC01g20290D:BnaCOMT1-12     (1) --------------------------------------------------------------------------------
               Bol013098      (1) --------------------------------------------------------------------------------
 BnaA03g48770D:BnaCOMT1-3     (1) --------------------------------------------------------------------------------
               Bra019031      (1) --------------------------------------------------------------------------------

 BnaA10g07270D:BnaCOMT1-5    (85) TTT----------ACTTTTGATAATGTTGTTTCATAAGCTATGAT----G--TGATAATTAAT---ATGCGATTGTTAAA
               Bra003007     (85) TTT----------ACTTTTGATAATGTTGTTTCATAAGCTATGAT----G--TGATAATTAAT---ATGCGATTGTTAAA
BnaC09g30360D:BnaCOMT1-10    (85) TTT----------ACTTTTGATAATGTTGTTTCATAAGCTATGAT----G--TGATAAACAAT---ATGCGATTGTTGAA
               Bol038837     (85) TTT----------ACTTTTGATAATGTTGTTTCATAAGCTATGAT----G--TGATAAACAAT---ATGCGATTGTTGAA
 BnaA02g09810D:BnaCOMT1-2    (81) TTT----------ATTATCGATAATGTTGTTTCATAAGCTATGCCTCCTGG-TTACCATATGC---AATTAATTGTTGAA
               Bra022700     (81) TTT----------ATTATCGATAATGTTGTTTCATAAGCTATGCTTCCTGG-TTACCATATGC---AATTAATTGTTGAA
 BnaC02g13760D:BnaCOMT1-7    (82) TTT----------ATTTTTGATTATGTTGTTTCATAAGCTATGCTTCCTGG-TTATCATCAT----ATGCAATTGTTGAA
               Bol037465     (82) TTT----------ATTTTTGATTATGTTGTTTCATAAGCTATGCTTCCTGG-TTATCATCAT----ATGCAATTGTTGAA
 BnaA03g11990D:BnaCOMT1-4    (78) TTAGAAAAAAAAGCTTCTTGATAATGTTGTTTCATAAGCTATGGTTCTTGGTTGATGTGAATC---ATGTGATGATTAAA
               Bra029041     (78) TTAGAAAAAAAAGCTTCTTGATAATGTTGTTTCATAAGCTATGGTTCTTGGTTGATGTGAATC---ATGTGATGATTAAA
 BnaC03g14720D:BnaCOMT1-8    (78) TT-----------CTT--TGATAATTTTGTTTCATTAGCTATAGTTCTTGGTTGATGTGAATC---ATGTGATGATTAAA
               Bol027968     (78) TT-----------CTT--TGATAATTTTGTTTCATTAGCTATAGTTCTTGGTTGATGTGAATC---ATGTGATGATTAAA
 BnaA10g07250D:BnaCOMT1-6  (1747) TTAAAG-TACTTCTACTTTAATAAGATAGATTTACAAGGTTGAGATCCACGCTTTGCGTGGGTTGAGATCCACGCTTTGC
               Bra003009   (1587) TTAAAG-TACTTCTACTTTAATAAGATAGATTTACAAGGTTGAGATCCACGCT-----------------------TTGC
 BnaAnng13760D:BnaCOMT1-1     (1) --------------------------------------------------------------------------------
               Bra026320      (1) --------------------------------------------------------------------------------
 BnaC07g40930D:BnaCOMT1-9     (1) --------------------------------------------------------------------------------
               Bol042352      (1) --------------------------------------------------------------------------------
BnaC01g20290D:BnaCOMT1-12     (1) --------------------------------------------------------------------------------
               Bol013098      (1) --------------------------------------------------------------------------------
 BnaA03g48770D:BnaCOMT1-3     (1) --------------------------------------------------------------------------------
               Bra019031      (1) --------------------------------------------------------------------------------

 BnaA10g07270D:BnaCOMT1-5   (146) AGGTAA-------A-GGTCAGAGAGTTG-GCCAC----CAAGTGA---------CAATCAGCCAAAACA---------GA
               Bra003007    (146) AGGTAA-------A-GGTCAGAGAGTTG-GCCAC----CAAGTGA---------CAATCAGCCAAAACA---------GA
BnaC09g30360D:BnaCOMT1-10   (146) AGGTAA-------AAGGTCAGAGAGTTG-GCCAC----CAAGTGA---------CAATCAGCCAAAACA---------GA
               Bol038837    (146) AGGTAA-------AAGGTCAGAGAGTTG-GCCAC----CAAGTGA---------CAATCAGCCAAAACA---------GA
 BnaA02g09810D:BnaCOMT1-2   (147) AGGAAAGCC-GTAAAGGTTAGAGAGTTA-GCCACGAAGCTAGTGATCAAAAAGACAATCAAGTAATCAACCATAAACAGA
               Bra022700    (147) AGGAAAGCC-GTAAAGGTTAGAGAGTTA-GCCACGAAGCTAGTGATCAAAAAGACAATCAAGTAATCAACCATAAACAGA
 BnaC02g13760D:BnaCOMT1-7   (147) ACTTAAACA-GACAAATTAATGGAT-------AC----CTGCTG-------------TCCACTAAAGAA-----------
               Bol037465    (147) ACTTAAACA-GACAAATTAATGGAT-------AC----CTGCTG-------------TCCACTAAAGAA-----------
 BnaA03g11990D:BnaCOMT1-4   (155) ATTGAATTGTGCAAAGGTAAAAGATGAGAGAGACTCGGCCACAAAG-----------TGTCATAGACAATCA------GA
               Bra029041    (155) ATTGAATTGTGCAAAGGTAAAAGATGAGAGAGACTCGGCCACAAAG-----------TGTCATAGACAATCA------GA
 BnaC03g14720D:BnaCOMT1-8   (142) ATTGAATTGTGCAAAGGTAAA-GATGAGAGAGACTCGGCCACAAAG-----------TGTCATAGACAATCA------GA
               Bol027968    (142) ATTGAATTGTGCAAAGGTAAA-GATGAGAGAGACTCGGCCACAAAG-----------TGTCATAGACAATCA------GA
 BnaA10g07250D:BnaCOMT1-6  (1826) GTGGGTTGAGACGAGTGTAAAAATTATATATGTCAGGTAAGGTTGTTCAATACAGATTTTGATCTGGTTTCAGTTCA---
               Bra003009   (1643) GTGGGTTGAGACGAGTGTAAAAATTATATATGTCAGGTAAGGTTGTTCAATACAGATTTTGATCTGGTTTCAGTTCA---
 BnaAnng13760D:BnaCOMT1-1     (1) --------------------------------------------------------------------------------
               Bra026320      (1) --------------------------------------------------------------------------------
 BnaC07g40930D:BnaCOMT1-9     (1) --------------------------------------------------------------------------------
               Bol042352      (1) --------------------------------------------------------------------------------
BnaC01g20290D:BnaCOMT1-12     (1) --------------------------------------------------------------------------------
               Bol013098      (1) --------------------------------------------------------------------------------
 BnaA03g48770D:BnaCOMT1-3     (1) --------------------------------------------------------------------------------
               Bra019031      (1) --------------------------------------------------------------------------------
```

```
   BnaA10g07270D:BnaCOMT1-5  (195) CAAAATGATGTCG----TTTGACACGTATTTGATACCTGCTGTCCACTAA---GAAATTATATTTATATACGA---T-TT
                  Bra003007  (195) CAAAATGATGTCG----TTTGACACGTATTTGATACCTGCTGTCCACTAA---GAAATTATATTTATATACGA---T-TT
  BnaC09g30360D:BnaCOMT1-10  (196) CAAAATGATGTCG----TTTGACACGTATTTGATACCTGCTGTCCACTAATAAGAAATTATATTTATATACGA---T-TT
                  Bol038837  (196) CAAAATGATGTCG----TTTGACACGTATTTGATACCTGCTGTCCACTAATAAGAAATTATATTTATATACGA---T-TT
   BnaA02g09810D:BnaCOMT1-2  (225) CACATTGATGATGTAGTTTTGACACTTATTCTATACCTTCTGTCCACTAA--ATAAATTATATTTATATACCTCTTTTTT
                  Bra022700  (225) CACATTGATGATGTAGTTTTGACACTTATTCTATACCTTCTGTCCACTAA--ATAAATTATATTTATATACCT-TTTTTT
   BnaC02g13760D:BnaCOMT1-7  (191) ---ATTGAT--------TCT-------ATTCTATACCTGCTGTCCACTAA--AGAAATTATATTTATATACCA---TTTT
                  Bol037465  (191) ---ATTGAT--------TCT-------ATTCTATACCTGCTGTCCACTAA--AGAAATTATATTTATATACCA---TTTT
   BnaA03g11990D:BnaCOMT1-4  (218) CAAAATGATGC------GTTGACACGTTTTCTATATCTGCT-TCCACTAA---AAAACTATATA-ATAT-----------
                  Bra029041  (218) CAAAATGATGC------GTTGACACGTTTTCTATATCTGCT-TCCACTAA---AAAACTATATA-ATAT-----------
   BnaC03g14720D:BnaCOMT1-8  (204) CAAAATGATGC------GCTGACACGTTTTCTATATCTGCT-TCCACTAA---AAAGCTATCTA-ATAT-----------
                  Bol027968  (204) CAAAATGATGC------GCTGACACGTTTTCTATATCTGCT-TCCACTAA---AAAGCTATCTA-ATAT-----------
   BnaA10g07250D:BnaCOMT1-6 (1903) -GTTTTTTGGTATTTTGGTTTATAAGAATTAGATACCATATTTAAACCAT-ATATATATAT--TGGTTTGGTTCGGGCTA
                  Bra003009 (1720) -GTTTTTTGGTATTTTGGTTTATAAGAATTAGATACCATATTTAAACCAT-ATATATATATATTGGTTTGGTTCGGGCTA
   BnaAnng13760D:BnaCOMT1-1    (1) ---------------GTAAGTACAAAACCCTAATAACACACTAACATCAT-AAAAACCATT-TCAATTTAATATAAGTGA
                  Bra026320    (1) ---------------GTAAGTACAAAACCCTAATAACACACTAACATCAT-AAAAACCATT-TCAATTTAATATAAGTGA
   BnaC07g40930D:BnaCOMT1-9    (1) ----------------------------GTATATAAAATATTAAAACCTT-TCAATATTCTAACTTGTATATTTTATATA
                  Bol042352    (1) ----------------------------GTATATAAAATATTAAAACCTT-TCAATATTCTAACTTGTATATTTTATATA
  BnaC01g20290D:BnaCOMT1-12    (1) --------------------------------------------------------------------------------
                  Bol013098    (1) --------------------------------------------------------------------------------
   BnaA03g48770D:BnaCOMT1-3    (1) --------------------------------------------------------------------------------
                  Bra019031    (1) --------------------------------------------------------------------------------
```

```
   BnaA10g07270D:BnaCOMT1-5  (264) CTTTT----AGTGA-TT-----AAATGAGCTCCATACATATATGAAAATTGTGTTTGTTACTGTACAACAAAAACAATAC
                  Bra003007  (264) CTTTT----AGTGA-TT-----AAATGAGCTCCATACATATATGAAAATTGTGTTTGTTACTGTACAACAAAAACAATAC
  BnaC09g30360D:BnaCOMT1-10  (268) CTTTT----AGTGA-TT-----AAATGAGCTCCATACATAGATGAAAATTGTGTTTGTTACTGTACAACAAAAACAATAC
                  Bol038837  (268) CTTTT----AGTGA-TT-----AAATGAGCTCCATACATAGATGAAAATTGTGTTTGTTACTGTACAACAAAAACAATAC
   BnaA02g09810D:BnaCOMT1-2  (303) TTTTTCTAAAGTGG-TTTGGTTATACGAGTTCCATAGTT-GATTTTTCTTTTGTTTGTTACTGTGC--CAAAATCA-TAC
                  Bra022700  (302) TTTTTCTAAAGTGG-TTTGGTTATACGAGTTCCATAGTT-GATTTTTCTTTTGTTTGTTACTGTGC--CAAAATCA-TAC
   BnaC02g13760D:BnaCOMT1-7  (248) TTTTTCTTTAGAGG-TT-----ATACGAGCTCCATAGTT-GATTTTTTGTTTGTTTGTTACTGTGC--CAAAATCA-TAC
                  Bol037465  (248) TTTTTCTTTAGAGG-TT-----ATACGAGCTCCATAGTT-GATTTTTTGTTTGTTTGTTACTGTGC--CAAAATCA-TAC
   BnaA03g11990D:BnaCOMT1-4  (276) -TTTT----AGTGG-TT-----AAGCGAGCTCTATCGTT----GAAATTTTTGTTTGCTA--GTAC--TAT--ACA----
                  Bra029041  (276) -TTTT----AGTGG-TT-----AAGCGAGCTCTATCGTT----GAAATTTTTGTTTGCTA--GTAC--TAT--ACA----
   BnaC03g14720D:BnaCOMT1-8  (262) -TTTT----AGTGG-TT-----AAGCGAGCTCTATCGTT----GAAATTTTTGTTTGCTA--GTAC--TAT--ACA----
                  Bol027968  (262) -TTTT----AGTGG-TT-----AAGCGAGCTCTATCGTT----GAAATTTTTGTTTGCTA--GTAC--TAT--ACA----
   BnaA10g07250D:BnaCOMT1-6 (1979) TATACTGTCAGT-T-TT--TTTATACCAAAAACTTAAAC--TATATTTATCTGTTATAATATTTTTTCAATTTAATTTTG
                  Bra003009 (1798) TATACTGTCAGT-T-TT--TTTATACCAAAAACTTAAAC--TATATTTATCTGTTATAATATTTTTTCAATTTAATTTTG
   BnaAnng13760D:BnaCOMT1-1   (64) CTTAATCTCAGT--G----CACCAATAAAAAAAAACACT--TTTAAAATTAGAAAAAACTATTTTCTCTATATAAGAAGT
                  Bra026320   (64) CTTAATCTCAGT--G----CACCAATAAAAAAAAACACT--TTTAAAATTAGAAAAAACTATTTTCTCTATATAAGAAGT
   BnaC07g40930D:BnaCOMT1-9   (52) TAATATGATCACCT-TT--CAATATTCTAACTTGTATAT--TTTATATATAATATGA-----------------------
                  Bol042352   (52) TAATATGATCACCT-TT--CAATATTCTAACTTGTATAT--TTTATATATAATATGATCACCTTTCAATATTCTAACTTG
  BnaC01g20290D:BnaCOMT1-12    (1) ------GTAAGT--GC---AAAACCCTAATAGAATATCC--TTTATATATAT-ATAAAAAAGAAGTAGAAAACCCTAATT
                  Bol013098    (1) ------GTAAGT--GC---AAAACCCTAATAGAATATCC--TTTATATATAT-AAAAAAAAGAAGTAGAAAACCCTAATT
   BnaA03g48770D:BnaCOMT1-3    (1) --------------------------------------------------------------------------------
                  Bra019031    (1) --------------------------------------------------------------------------------
```

**Repetitions** TCACCT-TT--CAATATTCTAACTTGTATAT--TTTATATATAATATGATCACCTTTCAATATTCTAACTTG

```
   BnaA10g07270D:BnaCOMT1-5  (334) AACTAATACGAAGAAAATTCAACAA--TAGATGTTTTAGTTGATCCCAGTCTAGAAGAAC--TAGAAAAATAACAAT---
                  Bra003007  (334) AACTAATACGAAGAAAATTCAACAA--TAGATGTTTTAGTTGATCCCAGTCTAGAAGAAC--TAGAAAAATAACAAT---
  BnaC09g30360D:BnaCOMT1-10  (338) AACTAATACGAAGAAAATTCAACAA--T---------------CCCA--CTAGAAGAAC--TAGAAAAATAACAAT---
                  Bol038837  (338) AACTAATACGAAGAAAATTCAACAA--T---------------CCCA--CTAGAAGAAC--TAGAAAAATAACAAT---
   BnaA02g09810D:BnaCOMT1-2  (378) AATCTAAA-GAATAATATTCAAAAA--GAAA---------T------ATTTTAGTTGAGG--TAGAG--AAAATTAA---
                  Bra022700  (377) AATCTAAA-GAATAATATTCAAAAA--GAAA---------T------ATTTTAGTTGAGG--TAGAG--AAAATTAA---
   BnaC02g13760D:BnaCOMT1-7  (318) AATCTAAA-GAATAATAATCAAAA---GAAA---------T------ATTTTTGTTGATCCCTAGAGCTAGAGGAAA---
                  Bol037465  (318) AATCTAAA-GAATAATAATCAAAA---GAAA---------T------ATTTTTGTTGATCCCTAGAGCTAGAGGAAA---
   BnaA03g11990D:BnaCOMT1-4  (331) AAATAATAAGAGGAAAATTAAAAAA--CACACTCTGT----------ACTCTAGTTGTTTCATAGCTTTAAATATTT---
                  Bra029041  (331) AAATAATAAGAGGAAAATTAAAAAA--CACACTCTGT----------ACTCTAGTTGTTTCATAGCTTTAAATATTT---
   BnaC03g14720D:BnaCOMT1-8  (317) AAATAATAAGAGGAAAATTAAAAAA--CACACTATGT----------ACTCTAGTTGTTTCATAGCTTTAAATATTT---
                  Bol027968  (317) AAATAATAAGAGGAAAATTAAAAAA--CACACTATGT----------ACTCTAGTTGTTTCATAGCTTTAAATATTT---
   BnaA10g07250D:BnaCOMT1-6 (2053) TATGATTAAAATTTGAATTTATTA---AACAGTACTATAATTTTTAAATATGATGTTTTCTATTTAATTATATTAT----
                  Bra003009 (1872) TATGATTAAAATTTGAATTTATTA---AACAGTACTATAATTTTTAAATATGATGTTTTCTATTTAATTTAAATAT-TCA
   BnaAnng13760D:BnaCOMT1-1  (136) TTTTCTAAAAGGAAAATATCGGGTTTTGAGATCGTATAAATATGGATATTTGATGCAATCAAGTTTTCT-CTCTAA----
                  Bra026320  (136) TTTTCTAAAAGGAAAATATCGGGTTTTGAGATCGTATAAATATGGATATTTGATGCAATCAAGTTTTCT-CTCTAA----
   BnaC07g40930D:BnaCOMT1-9  (104) -------------------------------------------------------------------TTACACTAA----
                  Bol042352  (127) TATATTTTATATATAATATGATCACCTTTCAATATTCTAACTTGTATATTTTATATAT--AATATGATTACACTAA----
  BnaC01g20290D:BnaCOMT1-12   (67) AATTATTAACATACTAACTTCATA---AAAACTATTTCAACTTA-ATATGTGGGTTTTCATAATTGATATAACAATC---
                  Bol013098   (67) AATTATTAACATACTAACTTCATA---AAAACTATTTCAACGTA-ATATGTGGGTTTTCATAATTGATATAACAATC---
   BnaA03g48770D:BnaCOMT1-3    (1) ------GTATATAAAATAT--------TCCAA----CCAACTTGTATATTTGATAAAT--AGTATTAT------------
                  Bra019031    (1) ------GTATATAAAATAT--------TCCAA----CCAATTTATATATTTGATAAAT--AATATTAT------------
```

TATATTTTATATATAATATGATCACCTTTCAATATTCTAACTTGTATATTTTATATAT--AATATGA

```
   BnaA10g07270D:BnaCOMT1-5  (407) --------------------------------------------------------------------------------
                  Bra003007  (407) --------------------------------------------------------------------------------
  BnaC09g30360D:BnaCOMT1-10  (393) --------------------------------------------------------------------------------
                  Bol038837  (393) --------------------------------------------------------------------------------
   BnaA02g09810D:BnaCOMT1-2  (433) --------------------------------------------------------------------------------
                  Bra022700  (432) --------------------------------------------------------------------------------
   BnaC02g13760D:BnaCOMT1-7  (376) --------------------------------------------------------------------------------
                  Bol037465  (376) --------------------------------------------------------------------------------
   BnaA03g11990D:BnaCOMT1-4  (396) --------------------------------------------------------------------------------
                  Bra029041  (396) --------------------------------------------------------------------------------
   BnaC03g14720D:BnaCOMT1-8  (382) --------------------------------------------------------------------------------
                  Bol027968  (382) --------------------------------------------------------------------------------
   BnaA10g07250D:BnaCOMT1-6 (2126) --------------------------------------------------------------------------------
                  Bra003009 (1948) AAATAACTAACAAATAAATTATAAGATTATAATTTCATGAGATGAAAACAAAAAAGATTAACAATATAAATAACTAAATT
   BnaAnng13760D:BnaCOMT1-1  (211) --------------------------------------------------------------------------------
                  Bra026320  (211) --------------------------------------------------------------------------------
   BnaC07g40930D:BnaCOMT1-9  (113) --------------------------------------------------------------------------------
                  Bol042352  (201) --------------------------------------------------------------------------------
  BnaC01g20290D:BnaCOMT1-12  (140) --------------------------------------------------------------------------------
                  Bol013098  (140) --------------------------------------------------------------------------------
   BnaA03g48770D:BnaCOMT1-3   (49) --------------------------------------------------------------------------------
                  Bra019031   (49) --------------------------------------------------------------------------------
```

```
 BnaA10g07270D:BnaCOMT1-5   (407) --------------------------------------------------------------------------------
                Bra003007   (407) --------------------------------------------------------------------------------
BnaC09g30360D:BnaCOMT1-10   (393) --------------------------------------------------------------------------------
                Bol038837   (393) --------------------------------------------------------------------------------
 BnaA02g09810D:BnaCOMT1-2   (433) --------------------------------------------------------------------------------
                Bra022700   (432) --------------------------------------------------------------------------------
 BnaC02g13760D:BnaCOMT1-7   (376) --------------------------------------------------------------------------------
                Bol037465   (376) --------------------------------------------------------------------------------
 BnaA03g11990D:BnaCOMT1-4   (396) --------------------------------------------------------------------------------
                Bra029041   (396) --------------------------------------------------------------------------------
 BnaC03g14720D:BnaCOMT1-8   (382) --------------------------------------------------------------------------------
                Bol027968   (382) --------------------------------------------------------------------------------
 BnaA10g07250D:BnaCOMT1-6  (2126) --------------------------------------------------------------------------------
                Bra003009  (2028) TTAGCAAAAATGTATGTCAATGAAAGTCTTTAGTTAAAAAAAAAATTATTCAAGTTAACGTTTTAAATATTAAAATCATA
 BnaAnng13760D:BnaCOMT1-1   (211) --------------------------------------------------------------------------------
                Bra026320   (211) --------------------------------------------------------------------------------
 BnaC07g40930D:BnaCOMT1-9   (113) --------------------------------------------------------------------------------
                Bol042352   (201) --------------------------------------------------------------------------------
BnaC01g20290D:BnaCOMT1-12   (140) --------------------------------------------------------------------------------
                Bol013098   (140) --------------------------------------------------------------------------------
 BnaA03g48770D:BnaCOMT1-3    (49) --------------------------------------------------------------------------------
                Bra019031    (49) --------------------------------------------------------------------------------

 BnaA10g07270D:BnaCOMT1-5   (407) --------------------------------------------------------------------------------
                Bra003007   (407) --------------------------------------------------------------------------------
BnaC09g30360D:BnaCOMT1-10   (393) --------------------------------------------------------------------------------
                Bol038837   (393) --------------------------------------------------------------------------------
 BnaA02g09810D:BnaCOMT1-2   (433) --------------------------------------------------------------------------------
                Bra022700   (432) --------------------------------------------------------------------------------
 BnaC02g13760D:BnaCOMT1-7   (376) --------------------------------------------------------------------------------
                Bol037465   (376) --------------------------------------------------------------------------------
 BnaA03g11990D:BnaCOMT1-4   (396) --------------------------------------------------------------------------------
                Bra029041   (396) --------------------------------------------------------------------------------
 BnaC03g14720D:BnaCOMT1-8   (382) --------------------------------------------------------------------------------
                Bol027968   (382) --------------------------------------------------------------------------------
 BnaA10g07250D:BnaCOMT1-6  (2126) --------------------------------------------------------------------------------
                Bra003009  (2108) CCAATAAACTGAACTATATATATATATATATCATCAATTATATTATGTAATTTATGTATAACTACATTAATATATTTTAA
 BnaAnng13760D:BnaCOMT1-1   (211) --------------------------------------------------------------------------------
                Bra026320   (211) --------------------------------------------------------------------------------
 BnaC07g40930D:BnaCOMT1-9   (113) --------------------------------------------------------------------------------
                Bol042352   (201) --------------------------------------------------------------------------------
BnaC01g20290D:BnaCOMT1-12   (140) --------------------------------------------------------------------------------
                Bol013098   (140) --------------------------------------------------------------------------------
 BnaA03g48770D:BnaCOMT1-3    (49) --------------------------------------------------------------------------------
                Bra019031    (49) --------------------------------------------------------------------------------

 BnaA10g07270D:BnaCOMT1-5   (407) --------------------------------------------------------------------------------
                Bra003007   (407) --------------------------------------------------------------------------------
BnaC09g30360D:BnaCOMT1-10   (393) --------------------------------------------------------------------------------
                Bol038837   (393) --------------------------------------------------------------------------------
 BnaA02g09810D:BnaCOMT1-2   (433) --------------------------------------------------------------------------------
                Bra022700   (432) --------------------------------------------------------------------------------
 BnaC02g13760D:BnaCOMT1-7   (376) --------------------------------------------------------------------------------
                Bol037465   (376) --------------------------------------------------------------------------------
 BnaA03g11990D:BnaCOMT1-4   (396) --------------------------------------------------------------------------------
                Bra029041   (396) --------------------------------------------------------------------------------
 BnaC03g14720D:BnaCOMT1-8   (382) --------------------------------------------------------------------------------
                Bol027968   (382) --------------------------------------------------------------------------------
 BnaA10g07250D:BnaCOMT1-6  (2126) --------------------------------------------------------------------------------
                Bra003009  (2188) TCTTTTTGTTTGGTTGGATCAATTTATATACTGAATCATATTCATTTACGTATAACTATATTACTATACTAACTTGTACT
 BnaAnng13760D:BnaCOMT1-1   (211) --------------------------------------------------------------------------------
                Bra026320   (211) --------------------------------------------------------------------------------
 BnaC07g40930D:BnaCOMT1-9   (113) --------------------------------------------------------------------------------
                Bol042352   (201) --------------------------------------------------------------------------------
BnaC01g20290D:BnaCOMT1-12   (140) --------------------------------------------------------------------------------
                Bol013098   (140) --------------------------------------------------------------------------------
 BnaA03g48770D:BnaCOMT1-3    (49) --------------------------------------------------------------------------------
                Bra019031    (49) --------------------------------------------------------------------------------

 BnaA10g07270D:BnaCOMT1-5   (407) --------------------------------------------------------------------------------
                Bra003007   (407) --------------------------------------------------------------------------------
BnaC09g30360D:BnaCOMT1-10   (393) --------------------------------------------------------------------------------
                Bol038837   (393) --------------------------------------------------------------------------------
 BnaA02g09810D:BnaCOMT1-2   (433) --------------------------------------------------------------------------------
                Bra022700   (432) --------------------------------------------------------------------------------
 BnaC02g13760D:BnaCOMT1-7   (376) --------------------------------------------------------------------------------
                Bol037465   (376) --------------------------------------------------------------------------------
 BnaA03g11990D:BnaCOMT1-4   (396) --------------------------------------------------------------------------------
                Bra029041   (396) --------------------------------------------------------------------------------
 BnaC03g14720D:BnaCOMT1-8   (382) --------------------------------------------------------------------------------
                Bol027968   (382) --------------------------------------------------------------------------------
 BnaA10g07250D:BnaCOMT1-6  (2126) --------------------------------------------------------------------------------
                Bra003009  (2268) TAATGTAATCAAAGTTTTTTTATTTTATAATTAAACCTAAAATAATTTTTGAAAATAGATCCCATCTTTCAAAGTAATAA
 BnaAnng13760D:BnaCOMT1-1   (211) --------------------------------------------------------------------------------
                Bra026320   (211) --------------------------------------------------------------------------------
 BnaC07g40930D:BnaCOMT1-9   (113) --------------------------------------------------------------------------------
                Bol042352   (201) --------------------------------------------------------------------------------
BnaC01g20290D:BnaCOMT1-12   (140) --------------------------------------------------------------------------------
                Bol013098   (140) --------------------------------------------------------------------------------
 BnaA03g48770D:BnaCOMT1-3    (49) --------------------------------------------------------------------------------
                Bra019031    (49) --------------------------------------------------------------------------------
```

```
BnaA10g07270D:BnaCOMT1-5   (407) --------------------------------------------------------------------------------
                Bra003007   (407) --------------------------------------------------------------------------------
BnaC09g30360D:BnaCOMT1-10  (393) --------------------------------------------------------------------------------
                Bol038837   (393) --------------------------------------------------------------------------------
 BnaA02g09810D:BnaCOMT1-2  (433) --------------------------------------------------------------------------------
                Bra022700   (432) --------------------------------------------------------------------------------
 BnaC02g13760D:BnaCOMT1-7  (376) --------------------------------------------------------------------------------
                Bol037465   (376) --------------------------------------------------------------------------------
 BnaA03g11990D:BnaCOMT1-4  (396) --------------------------------------------------------------------------------
                Bra029041   (396) --------------------------------------------------------------------------------
 BnaC03g14720D:BnaCOMT1-8  (382) --------------------------------------------------------------------------------
                Bol027968   (382) --------------------------------------------------------------------------------
 BnaA10g07250D:BnaCOMT1-6 (2126) --------------------------------------------------------------------------------
                Bra003009  (2348) CCGAACATAATATATAAATTTGTGATATAATTTCTTCAAAGAATATTTTTTTTTGGAATATATACTTTCATATGTTCCAT
 BnaAnng13760D:BnaCOMT1-1  (211) --------------------------------------------------------------------------------
                Bra026320   (211) --------------------------------------------------------------------------------
 BnaC07g40930D:BnaCOMT1-9  (113) --------------------------------------------------------------------------------
                Bol042352   (201) --------------------------------------------------------------------------------
BnaC01g20290D:BnaCOMT1-12  (140) --------------------------------------------------------------------------------
                Bol013098   (140) --------------------------------------------------------------------------------
 BnaA03g48770D:BnaCOMT1-3   (49) --------------------------------------------------------------------------------
                Bra019031    (49) --------------------------------------------------------------------------------

 BnaA10g07270D:BnaCOMT1-5  (407) --------------------------------------------------------------------------------
                Bra003007   (407) --------------------------------------------------------------------------------
BnaC09g30360D:BnaCOMT1-10  (393) --------------------------------------------------------------------------------
                Bol038837   (393) --------------------------------------------------------------------------------
 BnaA02g09810D:BnaCOMT1-2  (433) --------------------------------------------------------------------------------
                Bra022700   (432) --------------------------------------------------------------------------------
 BnaC02g13760D:BnaCOMT1-7  (376) --------------------------------------------------------------------------------
                Bol037465   (376) --------------------------------------------------------------------------------
 BnaA03g11990D:BnaCOMT1-4  (396) --------------------------------------------------------------------------------
                Bra029041   (396) --------------------------------------------------------------------------------
 BnaC03g14720D:BnaCOMT1-8  (382) --------------------------------------------------------------------------------
                Bol027968   (382) --------------------------------------------------------------------------------
 BnaA10g07250D:BnaCOMT1-6 (2126) --------------------------------------------------------------------------------
                Bra003009  (2428) AAATATATTGATTTTTCCTATTTGTTAGGATATATTTATGACGATATATCTTTTGATTCATATATACTTTGAAACAAAAA
 BnaAnng13760D:BnaCOMT1-1  (211) --------------------------------------------------------------------------------
                Bra026320   (211) --------------------------------------------------------------------------------
 BnaC07g40930D:BnaCOMT1-9  (113) --------------------------------------------------------------------------------
                Bol042352   (201) --------------------------------------------------------------------------------
BnaC01g20290D:BnaCOMT1-12  (140) --------------------------------------------------------------------------------
                Bol013098   (140) --------------------------------------------------------------------------------
 BnaA03g48770D:BnaCOMT1-3   (49) --------------------------------------------------------------------------------
                Bra019031    (49) --------------------------------------------------------------------------------

 BnaA10g07270D:BnaCOMT1-5  (407) --------------------------------------------------------------------------------
                Bra003007   (407) --------------------------------------------------------------------------------
BnaC09g30360D:BnaCOMT1-10  (393) --------------------------------------------------------------------------------
                Bol038837   (393) --------------------------------------------------------------------------------
 BnaA02g09810D:BnaCOMT1-2  (433) --------------------------------------------------------------------------------
                Bra022700   (432) --------------------------------------------------------------------------------
 BnaC02g13760D:BnaCOMT1-7  (376) --------------------------------------------------------------------------------
                Bol037465   (376) --------------------------------------------------------------------------------
 BnaA03g11990D:BnaCOMT1-4  (396) --------------------------------------------------------------------------------
                Bra029041   (396) --------------------------------------------------------------------------------
 BnaC03g14720D:BnaCOMT1-8  (382) --------------------------------------------------------------------------------
                Bol027968   (382) --------------------------------------------------------------------------------
 BnaA10g07250D:BnaCOMT1-6 (2126) --------------------------------------------------------------------------------
                Bra003009  (2508) AATAGTAAGAAAGATATTTTTTATTTAATACAAATGAGTTGTATATTGCCAAATACAGATTTGAAGTCAACTTGTATATA
 BnaAnng13760D:BnaCOMT1-1  (211) --------------------------------------------------------------------------------
                Bra026320   (211) --------------------------------------------------------------------------------
 BnaC07g40930D:BnaCOMT1-9  (113) --------------------------------------------------------------------------------
                Bol042352   (201) --------------------------------------------------------------------------------
BnaC01g20290D:BnaCOMT1-12  (140) --------------------------------------------------------------------------------
                Bol013098   (140) --------------------------------------------------------------------------------
 BnaA03g48770D:BnaCOMT1-3   (49) --------------------------------------------------------------------------------
                Bra019031    (49) --------------------------------------------------------------------------------

 BnaA10g07270D:BnaCOMT1-5  (407) --------------------------------------------------------------------------------
                Bra003007   (407) --------------------------------------------------------------------------------
BnaC09g30360D:BnaCOMT1-10  (393) --------------------------------------------------------------------------------
                Bol038837   (393) --------------------------------------------------------------------------------
 BnaA02g09810D:BnaCOMT1-2  (433) --------------------------------------------------------------------------------
                Bra022700   (432) --------------------------------------------------------------------------------
 BnaC02g13760D:BnaCOMT1-7  (376) --------------------------------------------------------------------------------
                Bol037465   (376) --------------------------------------------------------------------------------
 BnaA03g11990D:BnaCOMT1-4  (396) --------------------------------------------------------------------------------
                Bra029041   (396) --------------------------------------------------------------------------------
 BnaC03g14720D:BnaCOMT1-8  (382) --------------------------------------------------------------------------------
                Bol027968   (382) --------------------------------------------------------------------------------
 BnaA10g07250D:BnaCOMT1-6 (2126) --------------------------------------------------------------------------------
                Bra003009  (2588) ACGTGTATTGATTTAGTATTTGGTTTACATTTTCTCTCTAGGCTAATAAGAAAATTAATTGACTTCTAATCAGTCGACAG
 BnaAnng13760D:BnaCOMT1-1  (211) --------------------------------------------------------------------------------
                Bra026320   (211) --------------------------------------------------------------------------------
 BnaC07g40930D:BnaCOMT1-9  (113) --------------------------------------------------------------------------------
                Bol042352   (201) --------------------------------------------------------------------------------
BnaC01g20290D:BnaCOMT1-12  (140) --------------------------------------------------------------------------------
                Bol013098   (140) --------------------------------------------------------------------------------
 BnaA03g48770D:BnaCOMT1-3   (49) --------------------------------------------------------------------------------
                Bra019031    (49) --------------------------------------------------------------------------------
```

```
 BnaA10g07270D:BnaCOMT1-5   (407) --------------------------------------------------------------------------------
                Bra003007   (407) --------------------------------------------------------------------------------
BnaC09g30360D:BnaCOMT1-10   (393) --------------------------------------------------------------------------------
                Bol038837   (393) --------------------------------------------------------------------------------
 BnaA02g09810D:BnaCOMT1-2   (433) --------------------------------------------------------------------------------
                Bra022700   (432) --------------------------------------------------------------------------------
 BnaC02g13760D:BnaCOMT1-7   (376) --------------------------------------------------------------------------------
                Bol037465   (376) --------------------------------------------------------------------------------
 BnaA03g11990D:BnaCOMT1-4   (396) --------------------------------------------------------------------------------
                Bra029041   (396) --------------------------------------------------------------------------------
 BnaC03g14720D:BnaCOMT1-8   (382) --------------------------------------------------------------------------------
                Bol027968   (382) --------------------------------------------------------------------------------
 BnaA10g07250D:BnaCOMT1-6  (2126) --------------------------------------------------------------------------------
                Bra003009  (2668) ATCATTGTTTTGATCTGGTCCGGTATAAAAAATAGTTGAATACTCAACTAAAATGGATTAACCAACTACATGTACGTTTT
 BnaAnng13760D:BnaCOMT1-1   (211) --------------------------------------------------------------------------------
                Bra026320   (211) --------------------------------------------------------------------------------
 BnaC07g40930D:BnaCOMT1-9   (113) --------------------------------------------------------------------------------
                Bol042352   (201) --------------------------------------------------------------------------------
BnaC01g20290D:BnaCOMT1-12   (140) --------------------------------------------------------------------------------
                Bol013098   (140) --------------------------------------------------------------------------------
 BnaA03g48770D:BnaCOMT1-3    (49) --------------------------------------------------------------------------------
                Bra019031    (49) --------------------------------------------------------------------------------

 BnaA10g07270D:BnaCOMT1-5   (407) --------------------------------------------------------------------------------
                Bra003007   (407) --------------------------------------------------------------------------------
BnaC09g30360D:BnaCOMT1-10   (393) --------------------------------------------------------------------------------
                Bol038837   (393) --------------------------------------------------------------------------------
 BnaA02g09810D:BnaCOMT1-2   (433) --------------------------------------------------------------------------------
                Bra022700   (432) --------------------------------------------------------------------------------
 BnaC02g13760D:BnaCOMT1-7   (376) --------------------------------------------------------------------------------
                Bol037465   (376) --------------------------------------------------------------------------------
 BnaA03g11990D:BnaCOMT1-4   (396) --------------------------------------------------------------------------------
                Bra029041   (396) --------------------------------------------------------------------------------
 BnaC03g14720D:BnaCOMT1-8   (382) --------------------------------------------------------------------------------
                Bol027968   (382) --------------------------------------------------------------------------------
 BnaA10g07250D:BnaCOMT1-6  (2126) --------------------------------------------------------------------------------
                Bra003009  (2748) GTACGTTTTGGCGTTGCACCATCATCCTACATAGATGTTTTGGACATGCCCCAACATACAATTTAAAACCAACGAAGAAT
 BnaAnng13760D:BnaCOMT1-1   (211) --------------------------------------------------------------------------------
                Bra026320   (211) --------------------------------------------------------------------------------
 BnaC07g40930D:BnaCOMT1-9   (113) --------------------------------------------------------------------------------
                Bol042352   (201) --------------------------------------------------------------------------------
BnaC01g20290D:BnaCOMT1-12   (140) --------------------------------------------------------------------------------
                Bol013098   (140) --------------------------------------------------------------------------------
 BnaA03g48770D:BnaCOMT1-3    (49) --------------------------------------------------------------------------------
                Bra019031    (49) --------------------------------------------------------------------------------

 BnaA10g07270D:BnaCOMT1-5   (407) --------------------------------------------------------------------------------
                Bra003007   (407) --------------------------------------------------------------------------------
BnaC09g30360D:BnaCOMT1-10   (393) --------------------------------------------------------------------------------
                Bol038837   (393) --------------------------------------------------------------------------------
 BnaA02g09810D:BnaCOMT1-2   (433) --------------------------------------------------------------------------------
                Bra022700   (432) --------------------------------------------------------------------------------
 BnaC02g13760D:BnaCOMT1-7   (376) --------------------------------------------------------------------------------
                Bol037465   (376) --------------------------------------------------------------------------------
 BnaA03g11990D:BnaCOMT1-4   (396) --------------------------------------------------------------------------------
                Bra029041   (396) --------------------------------------------------------------------------------
 BnaC03g14720D:BnaCOMT1-8   (382) --------------------------------------------------------------------------------
                Bol027968   (382) --------------------------------------------------------------------------------
 BnaA10g07250D:BnaCOMT1-6  (2126) --------------------------------------------------------------------------------
                Bra003009  (2828) TATTCTGTACATGACAGTACAGAATAATAAAACAGTTGAAACAAATATGTTTTGAGCATAACAACGTATAACTATCTTAA
 BnaAnng13760D:BnaCOMT1-1   (211) --------------------------------------------------------------------------------
                Bra026320   (211) --------------------------------------------------------------------------------
 BnaC07g40930D:BnaCOMT1-9   (113) --------------------------------------------------------------------------------
                Bol042352   (201) --------------------------------------------------------------------------------
BnaC01g20290D:BnaCOMT1-12   (140) --------------------------------------------------------------------------------
                Bol013098   (140) --------------------------------------------------------------------------------
 BnaA03g48770D:BnaCOMT1-3    (49) --------------------------------------------------------------------------------
                Bra019031    (49) --------------------------------------------------------------------------------

 BnaA10g07270D:BnaCOMT1-5   (407) ---------------------AGAAAATACACTCTTTGTTCATTTTTGTTTCA------------------TCTAAACTA
                Bra003007   (407) ---------------------AGAAAATACACTCTTTGTTCATTTTTGTTTCA------------------TCTAAACTA
BnaC09g30360D:BnaCOMT1-10   (393) ---------------------AGAAAATACACTCTTTGTTCATTTTTGTTTCAACTATACTATTAAAAAGATCTATACTA
                Bol038837   (393) ---------------------AGAAAATACACTCTTTGTTCATTTTTGTTTCAACTATACTATTAAAAAGATCTATACTA
 BnaA02g09810D:BnaCOMT1-2   (433) --------------------------------------------------------------------------------
                Bra022700   (432) --------------------------------------------------------------------------------
 BnaC02g13760D:BnaCOMT1-7   (376) --------------------------------------------------------------------------------
                Bol037465   (376) --------------------------------------------------------------------------------
 BnaA03g11990D:BnaCOMT1-4   (396) --------------------------------------------------------------------------------
                Bra029041   (396) --------------------------------------------------------------------------------
 BnaC03g14720D:BnaCOMT1-8   (382) --------------------------------------------------------------------------------
                Bol027968   (382) --------------------------------------------------------------------------------
 BnaA10g07250D:BnaCOMT1-6  (2126) --------------------------------------------------------------------------------
                Bra003009  (2908) CAAAACAATGCAGACAATTATTATTATGTCTAATTTGTATAAGGGATATGGCAGTGGGTCATAAGTATCAATCTTAATAA
 BnaAnng13760D:BnaCOMT1-1   (211) --------------------------------------------------------------------------------
                Bra026320   (211) --------------------------------------------------------------------------------
 BnaC07g40930D:BnaCOMT1-9   (113) --------------------------------------------------------------------------------
                Bol042352   (201) --------------------------------------------------------------------------------
BnaC01g20290D:BnaCOMT1-12   (140) --------------------------------------------------------------------------------
                Bol013098   (140) --------------------------------------------------------------------------------
 BnaA03g48770D:BnaCOMT1-3    (49) --------------------------------------------------------------------------------
                Bra019031    (49) --------------------------------------------------------------------------------
```

```
  BnaA10g07270D:BnaCOMT1-5   (448) TTAAAAAA-AATCACTCCAAAAATCTACTT-AAAAAATTTGTTGCACTTATTCATTAACCAATTAATTTTTTGGTCCTAC
                 Bra003007   (448) TTAAAAAA-AATCACTCCAAAAATCTACTTTAAAAAAATTGTTGCACTTATTCATTAACCAATTAATTTTTTGGTCCTAC
 BnaC09g30360D:BnaCOMT1-10   (452) TTAAAAAAGAATCACTCTAATAATGTACTTAAAAAAAAATTGTTGCACCTATTCATTAACCAATTAATTTTTTGATCCTAC
                 Bol038837   (452) TTAAAAAAGAATCACTCTAATAATGTACTTAAAAAAAAATTGTTGCACCTATTCATTAACCAATTAATTTTTTGATCCTAC
  BnaA02g09810D:BnaCOMT1-2   (433) --------------------------------------------------------------------------------
                 Bra022700   (432) --------------------------------------------------------------------------------
  BnaC02g13760D:BnaCOMT1-7   (376) --------------------------------------------------------------------------------
                 Bol037465   (376) --------------------------------------------------------------------------------
  BnaA03g11990D:BnaCOMT1-4   (396) --------------------------------------------------------------------------------
                 Bra029041   (396) --------------------------------------------------------------------------------
  BnaC03g14720D:BnaCOMT1-8   (382) --------------------------------------------------------------------------------
                 Bol027968   (382) --------------------------------------------------------------------------------
  BnaA10g07250D:BnaCOMT1-6  (2126) --------------------------------------------------------------------------------
                 Bra003009  (2988) GTTGTGTAAGAATACTTTTTAATGTTGTCAAAAAAAATTGTTGGTCTAAGGTTATATGTGTAGTTAATTAACTGATATGC
  BnaAnng13760D:BnaCOMT1-1   (211) --------------------------------------------------------------------------------
                 Bra026320   (211) --------------------------------------------------------------------------------
  BnaC07g40930D:BnaCOMT1-9   (113) --------------------------------------------------------------------------------
                 Bol042352   (201) --------------------------------------------------------------------------------
 BnaC01g20290D:BnaCOMT1-12   (140) --------------------------------------------------------------------------------
                 Bol013098   (140) --------------------------------------------------------------------------------
  BnaA03g48770D:BnaCOMT1-3    (49) --------------------------------------------------------------------------------
                 Bra019031    (49) --------------------------------------------------------------------------------

  BnaA10g07270D:BnaCOMT1-5   (526) CTTATATTTCCTAATATATATCTAACTATACTAATCATAGTTAATGCTCTCTAACTATACATACTTTATTTTGAACCACC
                 Bra003007   (527) CTTATATTTTCTAATATATATCTAACTATACTAATCATAGTTAATGCTCTCTAACTATACATACTTTATTTTGAACCACC
 BnaC09g30360D:BnaCOMT1-10   (532) TTTAAATTTCTTAATATTTATCTAACTATACTAATCATAGTTAATGCTCTCTAACAATACATACTTTATTTTGAACCACT
                 Bol038837   (532) TTTAAATTTCTTAATATTTATCTAACTATACTAATCATAGTTAATGCTCTCTAACAATACATACTTTATTTTGAACCACT
  BnaA02g09810D:BnaCOMT1-2   (433) --------------------------------------------------------------------------------
                 Bra022700   (432) --------------------------------------------------------------------------------
  BnaC02g13760D:BnaCOMT1-7   (376) --------------------------------------------------------------------------------
                 Bol037465   (376) --------------------------------------------------------------------------------
  BnaA03g11990D:BnaCOMT1-4   (396) --------------------------------------------------------------------------------
                 Bra029041   (396) --------------------------------------------------------------------------------
  BnaC03g14720D:BnaCOMT1-8   (382) --------------------------------------------------------------------------------
                 Bol027968   (382) --------------------------------------------------------------------------------
  BnaA10g07250D:BnaCOMT1-6  (2126) --------------------------------------------------------------------------------
                 Bra003009  (3068) ATAAAGATAGCTGTGAATTTATTATATGTCTGATTAATAAATTTTTCTAGAGAATAAAAAGTTGGTCTAAGTTTGTCCAA
  BnaAnng13760D:BnaCOMT1-1   (211) --------------------------------------------------------------------------------
                 Bra026320   (211) --------------------------------------------------------------------------------
  BnaC07g40930D:BnaCOMT1-9   (113) --------------------------------------------------------------------------------
                 Bol042352   (201) --------------------------------------------------------------------------------
 BnaC01g20290D:BnaCOMT1-12   (140) --------------------------------------------------------------------------------
                 Bol013098   (140) --------------------------------------------------------------------------------
  BnaA03g48770D:BnaCOMT1-3    (49) --------------------------------------------------------------------------------
                 Bra019031    (49) --------------------------------------------------------------------------------

  BnaA10g07270D:BnaCOMT1-5   (606) GTTATTTTTACCATCTATATTATTAGAAAA--ATCACTCCAAAAATCTACTTAAAAAAATTGTTTCACCTATTCATTAAC
                 Bra003007   (607) GTTATTTTTACCATCTATATTATTAGAAAA--ATCACTCCAAAAATCTACTTAAAAAAATTGTTTCACCTATTCATTAAC
 BnaC09g30360D:BnaCOMT1-10   (612) ATTATTTTTACCATCTATACTATTAAAAAAGAATCATTCCAAAAATCTACTTAAAAAA-TTGTTGCAGCTATTCATTAAC
                 Bol038837   (612) ATTATTTTTACCATCTATACTATTAAAAAAGAATCATTCCAAAAATCTACTTAAAAAA-TTGTTGCAGCTATTCATTAAC
  BnaA02g09810D:BnaCOMT1-2   (433) --------------------------------------------------------------------------------
                 Bra022700   (432) --------------------------------------------------------------------------------
  BnaC02g13760D:BnaCOMT1-7   (376) --------------------------------------------------------------------------------
                 Bol037465   (376) --------------------------------------------------------------------------------
  BnaA03g11990D:BnaCOMT1-4   (396) --------------------------------------------------------------------------------
                 Bra029041   (396) --------------------------------------------------------------------------------
  BnaC03g14720D:BnaCOMT1-8   (382) --------------------------------------------------------------------------------
                 Bol027968   (382) --------------------------------------------------------------------------------
  BnaA10g07250D:BnaCOMT1-6  (2126) --------------------------------------------------------------------------------
                 Bra003009  (3148) ATTAGTTGTAAGACTGCCACGTAATCAAATATAGAGATTTAAAATAAAATGCTGACAATTCAGTAAAATTAAAATGTAAT
  BnaAnng13760D:BnaCOMT1-1   (211) --------------------------------------------------------------------------------
                 Bra026320   (211) --------------------------------------------------------------------------------
  BnaC07g40930D:BnaCOMT1-9   (113) --------------------------------------------------------------------------------
                 Bol042352   (201) --------------------------------------------------------------------------------
 BnaC01g20290D:BnaCOMT1-12   (140) --------------------------------------------------------------------------------
                 Bol013098   (140) --------------------------------------------------------------------------------
  BnaA03g48770D:BnaCOMT1-3    (49) --------------------------------------------------------------------------------
                 Bra019031    (49) --------------------------------------------------------------------------------

  BnaA10g07270D:BnaCOMT1-5   (684) CAATTAT-TTTTTTGGCTCTATCTTATATTTTCTAATATTTATCTAACTATACTAACCATAGTTAATATTCTCTAACTAT
                 Bra003007   (685) CAATTATCTTTTTTGGCTCTATCTTATATTTTCTAATATTTATCTAACTATACTAACCATAGTTAATATTCTCTAACTAT
 BnaC09g30360D:BnaCOMT1-10   (691) CAATTAATTTTTTTGGTTCTACCTTATATTTCCTAATATTTATCTAACTATACTAACCATAGTTAATGCTCTCTAACTAT
                 Bol038837   (691) CAATTAATTTTTTTGGTTCTACCTTATATTTCCTAATATTTATCTAACTATACTAACCATAGTTAATGCTCTCTAACTAT
  BnaA02g09810D:BnaCOMT1-2   (433) --------------------------------------------------------------------------------
                 Bra022700   (432) --------------------------------------------------------------------------------
  BnaC02g13760D:BnaCOMT1-7   (376) --------------------------------------------------------------------------------
                 Bol037465   (376) --------------------------------------------------------------------------------
  BnaA03g11990D:BnaCOMT1-4   (396) --------------------------------------------------------------------------------
                 Bra029041   (396) --------------------------------------------------------------------------------
  BnaC03g14720D:BnaCOMT1-8   (382) --------------------------------------------------------------------------------
                 Bol027968   (382) --------------------------------------------------------------------------------
  BnaA10g07250D:BnaCOMT1-6  (2126) --------------------------------------------------------------------------------
                 Bra003009  (3228) TAATACAAAATAGAAGGTTAGATCTTTTTAAATGATCTTCAAATCATATACAGTAGAACCTTTACAATTAATAATGTTGG
  BnaAnng13760D:BnaCOMT1-1   (211) --------------------------------------------------------------------------------
                 Bra026320   (211) --------------------------------------------------------------------------------
  BnaC07g40930D:BnaCOMT1-9   (113) --------------------------------------------------------------------------------
                 Bol042352   (201) --------------------------------------------------------------------------------
 BnaC01g20290D:BnaCOMT1-12   (140) --------------------------------------------------------------------------------
                 Bol013098   (140) --------------------------------------------------------------------------------
  BnaA03g48770D:BnaCOMT1-3    (49) --------------------------------------------------------------------------------
                 Bra019031    (49) --------------------------------------------------------------------------------
```

```
BnaA10g07270D:BnaCOMT1-5   (763) ACATACTTTATTTTGAACCACCGTTATTTTTACCATCTACATATATACTAATTTTTTGGTATACCAAATTTTTTACATAC
                 Bra003007   (765) ACATACTTTATTTTGAACCACCGTTATTTTTACCATCTACATATATACTAATTTTTTGGTATAGCAAATTTTTTACATAC
BnaC09g30360D:BnaCOMT1-10  (771) ACATACTTTATTTTGAACCACCATTATTTTTACAATCTACATATATACTATTTTTTTGGTATACCAAATTTTTTACATAC
                 Bol038837   (771) ACATACTTTATTTTGAACCACCATTATTTTTACAATCTACATATATACTATTTTTTTGGTATACCAAATTTTTTACATAC
BnaA02g09810D:BnaCOMT1-2   (433) --------------------------------------------------------------------------------
                 Bra022700   (432) --------------------------------------------------------------------------------
BnaC02g13760D:BnaCOMT1-7   (376) --------------------------------------------------------------------------------
                 Bol037465   (376) --------------------------------------------------------------------------------
BnaA03g11990D:BnaCOMT1-4   (396) --------------------------------------------------------------------------------
                 Bra029041   (396) --------------------------------------------------------------------------------
BnaC03g14720D:BnaCOMT1-8   (382) --------------------------------------------------------------------------------
                 Bol027968   (382) --------------------------------------------------------------------------------
BnaA10g07250D:BnaCOMT1-6  (2126) --------------------------------------------------------------------------------
                 Bra003009  (3308) GACTTTTGAAAATTTATTAATATATAGAGATATTAATTTCTAAAAGTTTCATTATTTAGATTTTTTATTTTAAGATATAT
BnaAnng13760D:BnaCOMT1-1   (211) --------------------------------------------------------------------------------
                 Bra026320   (211) --------------------------------------------------------------------------------
BnaC07g40930D:BnaCOMT1-9   (113) --------------------------------------------------------------------------------
                 Bol042352   (201) --------------------------------------------------------------------------------
BnaC01g20290D:BnaCOMT1-12  (140) --------------------------------------------------------------------------------
                 Bol013098   (140) --------------------------------------------------------------------------------
BnaA03g48770D:BnaCOMT1-3    (49) --------------------------------------------------------------------------------
                 Bra019031    (49) --------------------------------------------------------------------------------

BnaA10g07270D:BnaCOMT1-5   (843) ATAACTCCATCATACTAATCCATATAAATTCATTATACTAACATATGATCTTCTCTAACCGTATCTCATGAATCTCACGA
                 Bra003007   (845) ATAACTCCATCATACTAATCCATATAAATTCATTATACTAACATATGATCTTCTCTAACCGTATCTCATGAATCTCACGA
BnaC09g30360D:BnaCOMT1-10  (851) ATGACTCCATCATACTAATTCATATAAATTCATTATACTAACATATGATCTTCTCTAACCGTATCTCATGAATTTCACGA
                 Bol038837   (851) ATGACTCCATCATACTAATTCATATAAATTCATTATACTAACATATGATCTTCTCTAACCGTATCTCATGAATTTCACGA
BnaA02g09810D:BnaCOMT1-2   (433) --------------------------------------------------------------------------------
                 Bra022700   (432) --------------------------------------------------------------------------------
BnaC02g13760D:BnaCOMT1-7   (376) --------------------------------------------------------------------------------
                 Bol037465   (376) --------------------------------------------------------------------------------
BnaA03g11990D:BnaCOMT1-4   (396) --------------------------------------------------------------------------------
                 Bra029041   (396) --------------------------------------------------------------------------------
BnaC03g14720D:BnaCOMT1-8   (382) -----------TTGTTGTATTCACTCTAGAGGTTTTTTTTTAGAAATTCCGTGGAGTAACATTTCTAAATTTTTTTGTTA
                 Bol027968   (382) -----------TTGTTGTATTCACTCTAGAGGTTTTTTTTTAGAAATTCCGTGGAGTAACATTTCTAAGTTTTTT-GTTA
BnaA10g07250D:BnaCOMT1-6  (2126) --------------------------------------------------------------------------------
                 Bra003009  (3388) ATATTCTAAGATAACAAAAATATTTGATTTTTGTGTATAGACATTAATCGTTTTCTTCTGAAATTTGACATCTATATTAA
BnaAnng13760D:BnaCOMT1-1   (211) --------------------------------------------------------------------------------
                 Bra026320   (211) --------------------------------------------------------------------------------
BnaC07g40930D:BnaCOMT1-9   (113) --------------------------------------------------------------------------------
                 Bol042352   (201) --------------------------------------------------------------------------------
BnaC01g20290D:BnaCOMT1-12  (140) --------------------------------------------------------------------------------
                 Bol013098   (140) --------------------------------------------------------------------------------
BnaA03g48770D:BnaCOMT1-3    (49) --------------------------------------------------------------------------------
                 Bra019031    (49) --------------------------------------------------------------------------------

BnaA10g07270D:BnaCOMT1-5   (923) CCAAAACATTACTGATTTATCCAAATAAACAGAAATTATATTGTTTTTGGGTGCCATTTTTTTCCATTCATCCAACTGTG
                 Bra003007   (925) CCAAAACATTACTGATTTATCCAAATAAACAGAAATTATATTGTTTTTGGGTGCCATTTTTTTCCATTCATCCAACTGTG
BnaC09g30360D:BnaCOMT1-10  (931) CCAAAATATTACTAATTTATCCAAATAAAC-GAAA---------------------------------------------
                 Bol038837   (931) CCAAAATATTACTAATTTATCCAAATAAAC-GAAA---------------------------------------------
BnaA02g09810D:BnaCOMT1-2   (433) --------------------------------------------------------------------------------
                 Bra022700   (432) --------------------------------------------------------------------------------
BnaC02g13760D:BnaCOMT1-7   (376) --------------------------------------------------------------------------------
                 Bol037465   (376) --------------------------------------------------------------------------------
BnaA03g11990D:BnaCOMT1-4   (396) --------------------------------------------------------------------------------
                 Bra029041   (396) --------------------------------------------------------------------------------
BnaC03g14720D:BnaCOMT1-8   (451) CCAAAATAGTACTCAATAAAAAAAAATGACCAAAATAAATTTTATTAAATGGTAAAAATATATTTT-TACTTTAGAGTTAA
                 Bol027968   (450) CCAAAATAGTACTCAATAAAAAAAAATAACCAAAATAAATTTTATTAAATGGTAAAAATATATTTT-TACTTTAGAGTTAA
BnaA10g07250D:BnaCOMT1-6  (2126) --------------------------------------------------------------------------------
                 Bra003009  (3468) TTTTATTACATTATTTGGTGTATATAATAATGTTGGGACTTTGAAATTTTATTAATTTATAGAGATATTAATTTTAAAAA
BnaAnng13760D:BnaCOMT1-1   (211) --------------------------------------------------------------------------------
                 Bra026320   (211) --------------------------------------------------------------------------------
BnaC07g40930D:BnaCOMT1-9   (113) --------------------------------------------------------------------------------
                 Bol042352   (201) --------------------------------------------------------------------------------
BnaC01g20290D:BnaCOMT1-12  (140) --------------------------------------------------------------------------------
                 Bol013098   (140) --------------------------------------------------------------------------------
BnaA03g48770D:BnaCOMT1-3    (49) --------------------------------------------------------------------------------
                 Bra019031    (49) --------------------------------------------------------------------------------

BnaA10g07270D:BnaCOMT1-5  (1003) TCTATTTAAAACAAAAATCTCCTTTGATGTCTGATGTGACCTGATACATTATAGCTTCACTTTAATAACGGAGAATGTAA
                 Bra003007  (1005) TCTATTTAAAACAAAAATCTCCTTTGATGTCTGATGTGACCTGATACATTATAGCTTCACTTTAATAACGGAGAATGTAA
BnaC09g30360D:BnaCOMT1-10  (965) -----------------------------------------------ATTATAGCTTCGCTTTAATAACGGAGAATGTAA
                 Bol038837   (965) -----------------------------------------------ATTATAGCTTCGCTTTAATAACGGAGAATGTAA
BnaA02g09810D:BnaCOMT1-2   (433) --------------------------------------------------------------------------------
                 Bra022700   (432) --------------------------------------------------------------------------------
BnaC02g13760D:BnaCOMT1-7   (376) --------------------------------------------------------------------------------
                 Bol037465   (376) --------------------------------------------------------------------------------
BnaA03g11990D:BnaCOMT1-4   (396) --------------------------------------------------------------------------------
                 Bra029041   (396) --------------------------------------------------------------------------------
BnaC03g14720D:BnaCOMT1-8   (530) CTAATCTAAACTTATGGTTTAGAGTTAAAGAGGTGGAGTTTTGGGGTAGGGTTTAAAATTAAAAAAAAATTAAAATTAAA
                 Bol027968   (529) CTAATCTAAACTTATGGTTTAGAGTTAAAGAGGTGGAGTTTTGGGGTAGGGTTTAAAATTAAAAAAAAATTAAAATTAAA
BnaA10g07250D:BnaCOMT1-6  (2126) --------------------------------------------------------------------------------
                 Bra003009  (3548) AATTCATTATTTAGATTTCTTATTTTAAGATATATTTATTAAAAGATAAGAAAAATATTTGATTTTAGTGTATATCGTCA
BnaAnng13760D:BnaCOMT1-1   (211) --------------------------------------------------------------------------------
                 Bra026320   (211) --------------------------------------------------------------------------------
BnaC07g40930D:BnaCOMT1-9   (113) --------------------------------------------------------------------------------
                 Bol042352   (201) --------------------------------------------------------------------------------
BnaC01g20290D:BnaCOMT1-12  (140) --------------------------------------------------------------------------------
                 Bol013098   (140) --------------------------------------------------------------------------------
BnaA03g48770D:BnaCOMT1-3    (49) --------------------------------------------------------------------------------
                 Bra019031    (49) --------------------------------------------------------------------------------
```

```
 BnaA10g07270D:BnaCOMT1-5  (1083) ATGTAATATTCCAAACAATTTTTTTCCTCCTAAAGCAAAAATACGACATGTCAATACTTTTTTTCAAAAAACTCTTATTT
               Bra003007  (1085) ATGTAATATTCCAAACAATTTTTTTCCTCCTAAAGCAAAAATACGACATGTCAATACTTTTTTTCAAAAAACTCTTATTT
BnaC09g30360D:BnaCOMT1-10  (998) ATGTAATATTCCAAACAATTTTTTTCCTCCTAAAGCAAAAATACGACATGTCAATACTTTTATTCAAAAAACTCTTATTT
               Bol038837   (998) ATGTAATATTCCAAACAATTTTTTTCCTCCTAAAGCAAAAATACGACATGTCAATACTTTTATTCAAAAAACTCTTATTT
 BnaA02g09810D:BnaCOMT1-2  (433) --------------------------------------------------------------------------------
               Bra022700   (432) --------------------------------------------------------------------------------
 BnaC02g13760D:BnaCOMT1-7  (376) --------------------------------------------------------------------------------
               Bol037465   (376) --------------------------------------------------------------------------------
 BnaA03g11990D:BnaCOMT1-4  (396) ------------------------------------------------------------------------TTGTTGTA
               Bra029041   (396) ------------------------------------------------------------------------TTGTTGTA
 BnaC03g14720D:BnaCOMT1-8  (610) ATTAAAATTTTCAAAATAAAAGAGGTTATTT-AGTTATTTTCTTTCTGGAAGACTATTTTGCGAAAAAAAACTTAAAATA
               Bol027968   (609) ATTAAAATTTTCAAAATAAAAGAGGTTATTTTAGTTATTTTCTTTCTGGAAGACTATTTTGCTAAAAAAAACTTAAAATA
 BnaA10g07250D:BnaCOMT1-6 (2126) -----------------------------------------TTGGTGTATATAATATATATATTTCATAGAACTTAAATG
               Bra003009  (3628) TTTTCTGAAATTTGACATTTATATTAATTATATTATATTATTTGGTGTATATAATATATATATTTCATAGAACTTAAATG
 BnaAnng13760D:BnaCOMT1-1  (211) --------------------------------------------------------------------------------
               Bra026320   (211) --------------------------------------------------------------------------------
 BnaC07g40930D:BnaCOMT1-9  (113) --------------------------------------------------------------------------------
               Bol042352   (201) --------------------------------------------------------------------------------
BnaC01g20290D:BnaCOMT1-12  (140) --------------------------------------------------------------------------------
               Bol013098   (140) --------------------------------------------------------------------------------
 BnaA03g48770D:BnaCOMT1-3   (49) --------------------------------------------------------------------------------
               Bra019031    (49) --------------------------------------------------------------------------------

 BnaA10g07270D:BnaCOMT1-5 (1163) ATCTCA---AAACCATAAATATTATTTTAAAAAACTATCCTAAAAATCTATTGTTAAATGTTGTTGTACCTATTCATTTT
               Bra003007  (1165) ATCTCA---AAACCATAAATATTATTTTAAAAAACTATCCTAAAAATCTATTGTTAAATGTTGTTGTACCTATTCATTTT
BnaC09g30360D:BnaCOMT1-10 (1078) ATCTCA---AAACCATAAATATTATTTTAAAAAACTATCCTAAAAATCTATT-------GTTGTTGTACCTATTCATTTT
               Bol038837  (1078) ATCTCA---AAACCATAAATATTATTTTAAAAAACTATCCTAAAAATCTATT-------GTTGTTGTACCTATTCATTTT
 BnaA02g09810D:BnaCOMT1-2  (433) -----------------------------ACAATATTTTCTGGATGTTACCAG---AAAAAAACTATTATA--TGAT--T
               Bra022700   (432) -----------------------------ACAATATTTTCTGGATGTTACCAG---AAAAAAACTATTATA--TGAT--T
 BnaC02g13760D:BnaCOMT1-7  (376) -----------------------------ACAATATTTTCTGGATATTACCA----AAAAATACTATTATA--TGAT--T
               Bol037465   (376) -----------------------------ACAATATTTTCTGGATATTACCA----AAAAATACTATTATA--TGAT--T
 BnaA03g11990D:BnaCOMT1-4  (404) TTCAATGT-AGAGGTTTTTTTTTTTTTTGTCAGAAAACACTCTCCATCATTATT--ATT-TTATTTTGTGT-TTCATTTT
               Bra029041   (404) TTCAATGT-AGAGGTTTTTTTTTTTTTTTGCAGAAAACACTCTCCATCATTATT--ATT-TTATTTTGTGT-TTCATTTT
 BnaC03g14720D:BnaCOMT1-8  (689) AACTATTTGAAAGAATTTTTTTTTTTTTTGCAGAAAATACTCTCTATTATTATT--ATT-TTATTTTGTGT-TTCATTTT
               Bol027968   (689) AACTATTTGAAAGAATTTTTTTTTTTTTTGCAGAAAATACTCT--ATTATTATT--ATT-TTATTTTGTGT-TTCATTTT
 BnaA10g07250D:BnaCOMT1-6 (2165) TGGTTTTAAATATAAAATTACTAAATCTCATCAAAAATATTAAATGTTAAGAA---AATATAAAGATAATT--CCATT-G
               Bra003009  (3708) TGGTTTTAAATATAAAATTACTAAATCTCATCAAAAATATTAAATGTTAAGAA---AATATAAAGATAATT--CCATT-G
 BnaAnng13760D:BnaCOMT1-1  (211) ----------------------------------------------------GT--ATTCTTGCTTTGATT-TGCATTTG
               Bra026320   (211) ----------------------------------------------------GT--ATTCTTGCTTTGATT-TGCATTTG
 BnaC07g40930D:BnaCOMT1-9  (113) ---------------------------------------TCAAGATACAAG-----TTTATAATGATAT-----CATT-A
               Bol042352   (201) ---------------------------------------TCAAGATACAAG-----TTTATAATGATAT-----CATT-A
BnaC01g20290D:BnaCOMT1-12  (140) ----------------------------------------TGAAACTGTATGTCG--GTACCATTTAAAG----------
               Bol013098   (140) ----------------------------------------TGAAACTGTATGTCG--GTACCATTTAAAGGATGCAGTTC
 BnaA03g48770D:BnaCOMT1-3   (49) ----------------------------------------CAAAAAACAAG-----TTTATAATGATAT-----CAT--A
               Bra019031    (49) ----------------------------------------CAAAAAACAAGG----TTTATAATGATAT-----CAT--A

 BnaA10g07270D:BnaCOMT1-5 (1240) TAATTTAACATGTCTTATAGT---A-ATATAAGTTCTGAACCAATTCAATGATTATATTAAGGTAATTAAATCAACCA--
               Bra003007  (1242) TAATTTAACATGTCTTATAGT---A-ATATAAGTTCTGAACCAATTCAATGATTATATTTAAGTAATTAAATCAACCA--
BnaC09g30360D:BnaCOMT1-10 (1148) TAATTTAACATGTCTTATAGT---A-ATATAAGTTCTGAACCAAATCAATGATTATATTTAAGTAATTAAATCAACCA--
               Bol038837  (1148) TAATTTAACATGTCTTATAGT---A-ATATAAGTTCTGAACCAAATCAATGATTATATTTAAGTAATTAAATCAACCA--
 BnaA02g09810D:BnaCOMT1-2  (477) TCATTA--GATCTTGTGCTAAGCTAGCTAAACCTAGTTAGAAAAAAAAA-CTATTCTTTATAATTGATAATGTTGTTT--
               Bra022700   (476) TCATTA--GATCTTGTGCTAAGCTAGCTAAACCTAGTTAGAAAAAAAAA-CTATTCTTTATAATTGATAATGTTGTTT--
 BnaC02g13760D:BnaCOMT1-7  (419) TCAGTATAGATCTTGTGCTAAGATAGATAAACCTAGTTAGAAAAAAAAAACTGTTCTTTATGATTGATAATGTTGTTTCT
               Bol037465   (419) TCAGTATAGATCTTGTGCTAAGATAGATAAACCTAGTTAGAAAAAAAAAACTGTTCTTTATGATTGATAATGTTGTTTCT
 BnaA03g11990D:BnaCOMT1-4  (479) CTTGTCAACGTGTTTCATGGTTCTAAATAGAGAAGATGTAATTGTTCTTCCCTTTATTGAGGATCATTTAAGGAAACT--
               Bra029041   (479) CTTGTCAACGTGTTTCATGGTTCTAAATAGAGAAGATGTAATTGTTCTTCCCTTTATTGAGGATCATTTAAGGAAACT--
 BnaC03g14720D:BnaCOMT1-8  (765) CTTGTCAACGTGTTTCATGGTTCTAAATAGAGAAGATGTAATTGTTCTTCCCTTTACTGAGGATCATTTAAGGAAACT--
               Bol027968   (763) CTTGTCAACGTGTTTCATGGTTCTAAATAGAGAAGATGTAATTGTTCTTCCCTTTACTGAGGATCATTTAAGGAAACT--
 BnaA10g07250D:BnaCOMT1-6 (2239) TGAGTATAAAACAAACATTATAATAAAACATACTTATTTATATAAAATATGCATAGATATAAATTATTATTTTATGATTT
               Bra003009  (3782) TGAGTATAAAACAAACATTATAATAAAACATACTAATTTATATAAAATATGCATAGATATAAATTATTATTTTATGATTT
 BnaAnng13760D:BnaCOMT1-1  (236) TTCATAACAG----------------------------------------------------------------------
               Bra026320   (236) TTCATAACAG----------------------------------------------------------------------
 BnaC07g40930D:BnaCOMT1-9  (143) TGAGTCCAG-----------------------------------------------------------------------
               Bol042352   (231) TGAGTCCAG-----------------------------------------------------------------------
BnaC01g20290D:BnaCOMT1-12  (168) --------------------------------------------------------------------------------
               Bol013098   (178) TTGAAGGAGGGATCCCATTCGATAAGGGATACGGTATGTCAACCTTCGTTTACCACTGGAAAGATCAGCGATTTGCAAAT
 BnaA03g48770D:BnaCOMT1-3   (77) TGAGTGTAG-----------------------------------------------------------------------
               Bra019031    (78) TGAGTGTAG-----------------------------------------------------------------------

 BnaA10g07270D:BnaCOMT1-5 (1314) AATTTATGGCGACATTTGATATTCAAATTATTTTTGGTCATATCATTAATTAATTTAACTATATAATATTTTTAATTGAA
               Bra003007  (1316) AATTTATGGCGACATTTGATATTCAAATTATTTTTGGTCATATCATTAATTAATTTAACTATATAATATTTTTAATTGAA
BnaC09g30360D:BnaCOMT1-10 (1222) AATTTATGGCGACATTTGATATTCAAATTATTTTTGGTCATATCATTAATCAATTTAACTATATAATATTTTTAATTGAA
               Bol038837  (1222) AATTTATGGCGACATTTGATATTCAAATTATTTTTGGTCATATCATTAATCAATTTAACTATATAATATTTTTAATTGAA
 BnaA02g09810D:BnaCOMT1-2  (552) -ACTTGTTTCAG--------------------------------------------------------------------
               Bra022700   (551) -ACTTGTTTCAG--------------------------------------------------------------------
 BnaC02g13760D:BnaCOMT1-7  (499) TACTCATGCCAACGATACTTGTTTCAG-----------------------------------------------------
               Bol037465   (499) TACTCATGCCAACGATACTTGTTTCAG-----------------------------------------------------
 BnaA03g11990D:BnaCOMT1-4  (557) ATAATAAGAAGAGAACCGGTCTTCCGGTTATTTAG---CAGCTAGCAAAGGAATATAGAAACATAAT--TTCAAACCGGC
               Bra029041   (557) ATAATAAGAAGAGAACCGGTCTTCCGGTTATTTAG---CAGCTAGCAAAGGAATATAGAAACATAAT--TTCAAACCGGC
 BnaC03g14720D:BnaCOMT1-8  (843) ATAATAAGAAGAGAACCGGTCTTCCGGTTATTTAG---CAGCTAGCAAAGGAATATAGAAACATAAT--CTCAAACCGGC
               Bol027968   (841) ATAATAAGAAGAGAACCGGTCTTCCGGTTATTTAG---CAGCTAGCAAAGGAATATAGAAACATAAT--CTCAAACCGGC
 BnaA10g07250D:BnaCOMT1-6 (2319) TAATGGGACCATATATTTGCATAAAAGTTTCTAAAAAATTATTATCTTATTATTTTAACGATTTTTGTCAAATTTTGAAC
               Bra003009  (3862) TAATGGGACCATATATTTGCATAAAAGTTTCTAAAAAATTATTATCTTATTATTTTAACGATTTTTGTCAAATTTTGAAC
 BnaAnng13760D:BnaCOMT1-1  (246) --------------------------------------------------------------------------------
               Bra026320   (246) --------------------------------------------------------------------------------
 BnaC07g40930D:BnaCOMT1-9  (152) --------------------------------------------------------------------------------
               Bol042352   (240) --------------------------------------------------------------------------------
BnaC01g20290D:BnaCOMT1-12  (168) --------------------------------------------------------------------------------
               Bol013098   (258) GTATTTAG------------------------------------------------------------------------
 BnaA03g48770D:BnaCOMT1-3   (86) --------------------------------------------------------------------------------
               Bra019031    (87) --------------------------------------------------------------------------------
```

```
  BnaA10g07270D:BnaCOMT1-5  (1394) TTTTACTATTACTCCTCATTTCATGTGTTATATTTCTTCTCTAAATATTTTTAATTGATTTTGATAAAGAGTATTTAGAA
                Bra003007  (1396) TTTTACTATTACTCCTCATTTCATGTGTTATATTTCTTCTCTAAATATTTTTAATTGATTTTGATAAAGAGTATTTAGAA
 BnaC09g30360D:BnaCOMT1-10  (1302) TTATACTGTTACTCCTCATTTCATGTGATATATTTCTTCTCTAAATATTTTTAATTGATTTTCATAAGAAGTATTTGGAA
                Bol038837  (1302) TTATACTGTTACTCCTCATTTCATGTGATATATTTCTTCTCTAAATATTTTTAATTGATTTTCATAAGAAGTATTTGGAA
  BnaA02g09810D:BnaCOMT1-2   (563) --------------------------------------------------------------------------------
                Bra022700   (562) --------------------------------------------------------------------------------
  BnaC02g13760D:BnaCOMT1-7   (526) --------------------------------------------------------------------------------
                Bol037465   (526) --------------------------------------------------------------------------------
  BnaA03g11990D:BnaCOMT1-4   (632) TGGTTCTCTT--TGCATACCGGCAGCTCTATCATGATAACCAATTATAATCAACCTCAGGTGGATCAAGTCTGATCAATA
                Bra029041   (632) TGGTTCTCTT--TGCATACCGGCAGCTCTATCATGATAACCAATTATAATCAACCTCAGGTGGATCAAGTCTGATCAATA
  BnaC03g14720D:BnaCOMT1-8   (918) TGGTTCTCTT--TGCATACCGGCAGCTCTATCATGATAACCAATTATAATCAACCTCAGGTGGATCAAGTCTGATCAATA
                Bol027968   (916) TGGTTCTCTT--TGCATACCGGCAGCTCTATCATGATAACCAATTATAATCAACCTCAGGTGGATCAAGTCTGATCAATA
  BnaA10g07250D:BnaCOMT1-6  (2399) GAAACCAAGTTGGGACTGGCAAAATTTATTAATTTATAGAGATAATTAATTTATAGAATATTAATTAATAGAGATTCTAC
                Bra003009  (3942) GAAACCAAGTTGGGACTGGCAAAATTTATTAATTTATAGAGATAATTAATTTATAGATTATTAATTAATAGAGATTCTAC
  BnaAnng13760D:BnaCOMT1-1   (246) --------------------------------------------------------------------------------
                Bra026320   (246) --------------------------------------------------------------------------------
  BnaC07g40930D:BnaCOMT1-9   (152) --------------------------------------------------------------------------------
                Bol042352   (240) --------------------------------------------------------------------------------
 BnaC01g20290D:BnaCOMT1-12   (168) --------------------------------------------------------------------------------
                Bol013098   (266) --------------------------------------------------------------------------------
  BnaA03g48770D:BnaCOMT1-3    (86) --------------------------------------------------------------------------------
                Bra019031    (87) --------------------------------------------------------------------------------

  BnaA10g07270D:BnaCOMT1-5  (1474) TAAAAATTTGTTATTTAAGAAAAAAA-TTAAAACTATTAAAAACCATATAAATCTATACTATTAAATTAGCCCAAAATCT
                Bra003007  (1476) TAAAAATTTGTTATTTAAGAAAAAAA-TTAAAACTATTAAAAACCATATAAATCTATACTATTAAATTAGTCCAAAATCT
 BnaC09g30360D:BnaCOMT1-10  (1382) TAAAAATTTGCTATTTAAGAAAAAAAATTAAAACTATTAAAAACCATATAAATCTATACTATTAAATTAACCAAAAATCT
                Bol038837  (1382) TAAAAATTTGCTATTTAAGAAAAAAAATTAAAACTATTAAAAACCATATAAATCTATACTATTAAATTAACCAAAAATCT
  BnaA02g09810D:BnaCOMT1-2   (563) --------------------------------------------------------------------------------
                Bra022700   (562) --------------------------------------------------------------------------------
  BnaC02g13760D:BnaCOMT1-7   (526) --------------------------------------------------------------------------------
                Bol037465   (526) --------------------------------------------------------------------------------
  BnaA03g11990D:BnaCOMT1-4   (710) GATTATCTTGAGATCAATTTATAAAA---AAATATCCTAACTAGTG-ATAAATAAAAACGGTTAGATTAAGCTT---TGT
                Bra029041   (710) GATTATCTTGAGATCAATTTATAAAA---AAATATCCTAACTAGTG-ATAAATAAAAACGGTTAGATTAAGCTT---TGT
  BnaC03g14720D:BnaCOMT1-8   (996) GATTATCGTGAGACCAATTTATAAAA---AAATATCCTAACTAGTG-ATAAATAAAAACGGTTAGATTAAGCTT---TGT
                Bol027968   (994) GATTATCGTGAGATCAATTTATAAAA---AAATATCCTAACTAGTG-ATAAATAAAAACGGTTAGATTAAGCTT---TGT
  BnaA10g07250D:BnaCOMT1-6  (2479) TGTATATAGGGGATTAGAAAAGACTATGTCTCAACAGTATAATATATATTTGATAGTTTTCAATAAGTTATAATATAAAT
                Bra003009  (4022) TGTATATAGGGGATTAGAAAAGACTATGTCTCAACAGTATAATATATATTTGATAGTTTTCAATAAGTTATAATATAAAT
  BnaAnng13760D:BnaCOMT1-1   (246) --------------------------------------------------------------------------------
                Bra026320   (246) --------------------------------------------------------------------------------
  BnaC07g40930D:BnaCOMT1-9   (152) --------------------------------------------------------------------------------
                Bol042352   (240) --------------------------------------------------------------------------------
 BnaC01g20290D:BnaCOMT1-12   (168) --------------------------------------------------------------------------------
                Bol013098   (266) --------------------------------------------------------------------------------
  BnaA03g48770D:BnaCOMT1-3    (86) --------------------------------------------------------------------------------
                Bra019031    (87) --------------------------------------------------------------------------------

  BnaA10g07270D:BnaCOMT1-5  (1553) GTCAATTAAATTTTAAGTCTCAACAACTCAACAAATATAATTCGTATAAAATACACACAAAATATAATAAGATAAAGTAG
                Bra003007  (1555) GTCAATTAAATTTTAAGTCTCAACAACTCAACAAATATAATTCGTATAAAATACACACAAAATATAATAAGATAAAGTAG
 BnaC09g30360D:BnaCOMT1-10  (1462) GCCAA----ATCTTAAGTCTCACCAACTCAACAAATATAACTCTTATAAAATGCATACAAAATATAAAAAGATAAAGTAG
                Bol038837  (1462) GCCAA----ATCTTAAGTCTCACCAACTCAACAAATATAACTCTTATAAAATGCATACAAAATATAAAAAGATAAAGTAG
  BnaA02g09810D:BnaCOMT1-2   (563) --------------------------------------------------------------------------------
                Bra022700   (562) --------------------------------------------------------------------------------
  BnaC02g13760D:BnaCOMT1-7   (526) --------------------------------------------------------------------------------
                Bol037465   (526) --------------------------------------------------------------------------------
  BnaA03g11990D:BnaCOMT1-4   (783) GTGAATGT-GTGTGTTTTCTTAAACTCATACCAACGATACTTGTTTCAG-------------------------------
                Bra029041   (783) GTGAATGT-GTGTGTTTTCTTAAACTCATACCAACGATACTTGTTTCAG-------------------------------
  BnaC03g14720D:BnaCOMT1-8  (1069) GTGAATGT-GTGTGTTTTCTTAAACTCATACCAAAGATACTTGTTTCAG-------------------------------
                Bol027968  (1067) GTGAATGT-GTGTGTTTTCTTAAACTCATACCAAAGATACTTGTTTCAG-------------------------------
  BnaA10g07250D:BnaCOMT1-6  (2559) GCAAATTGTGTCATACAATTTCAGAACACATTTATAATTAGTGATAAAGACTTAATTGATCTTTTAAGAAAAATGTTGAT
                Bra003009  (4102) GCAAATTGTGTCATACAATTTCAGAACACATTTATAATTAGTGATAAAGACTTAATTGATCTTTTAAGAAAAATGTTGAT
  BnaAnng13760D:BnaCOMT1-1   (246) --------------------------------------------------------------------------------
                Bra026320   (246) --------------------------------------------------------------------------------
  BnaC07g40930D:BnaCOMT1-9   (152) --------------------------------------------------------------------------------
                Bol042352   (240) --------------------------------------------------------------------------------
 BnaC01g20290D:BnaCOMT1-12   (168) --------------------------------------------------------------------------------
                Bol013098   (266) --------------------------------------------------------------------------------
  BnaA03g48770D:BnaCOMT1-3    (86) --------------------------------------------------------------------------------
                Bra019031    (87) --------------------------------------------------------------------------------

  BnaA10g07270D:BnaCOMT1-5  (1633) ATATGTGCATTTGGGTCTTGGGTTTGGTTATGTATTGGTTTCTTTCGGGTCCGGATCTTCTAGGTC--AAAGTTTAAGG-
                Bra003007  (1635) ATATGTGCATTTGGGTCTTGGGTTTGGTTATGTATTGGTTTCTTTCGGGTCCGGATCTTCTAGGTC--AAAGTTTAAGG-
 BnaC09g30360D:BnaCOMT1-10  (1538) ACATGTGCATTTGGGTCTTGGGTTTGGT-ATGTATTGGTTTCTTTCGGATCCGGATCTTCTAGGTCCTAAACATTTAGAC
                Bol038837  (1538) ACATGTGCATTTGGGTCTTGGGTTTGGT-ATGTATTGGTTTCTTTCGGATCCGGATCTTCTAGGTCCTAAACATTTAGAC
  BnaA02g09810D:BnaCOMT1-2   (563) --------------------------------------------------------------------------------
                Bra022700   (562) --------------------------------------------------------------------------------
  BnaC02g13760D:BnaCOMT1-7   (526) --------------------------------------------------------------------------------
                Bol037465   (526) --------------------------------------------------------------------------------
  BnaA03g11990D:BnaCOMT1-4   (831) --------------------------------------------------------------------------------
                Bra029041   (831) --------------------------------------------------------------------------------
  BnaC03g14720D:BnaCOMT1-8  (1117) --------------------------------------------------------------------------------
                Bol027968  (1115) --------------------------------------------------------------------------------
  BnaA10g07250D:BnaCOMT1-6  (2639) GGGTTAGTTAAGCTAAAGGAGAGATTGTTTGTTTGTTTGTTTTGTTAAACTCATGCCAAAGAGTTTTGTTGCAG------
                Bra003009  (4182) GGGTTAGTTAAGCTAAAGGAGAGATTGTTTGTTTGTTTGTTTTGTTAAACTCATGCCAAAGAGTTTTGTTGCAG------
  BnaAnng13760D:BnaCOMT1-1   (246) --------------------------------------------------------------------------------
                Bra026320   (246) --------------------------------------------------------------------------------
  BnaC07g40930D:BnaCOMT1-9   (152) --------------------------------------------------------------------------------
                Bol042352   (240) --------------------------------------------------------------------------------
 BnaC01g20290D:BnaCOMT1-12   (168) --------------------------------------------------------------------------------
                Bol013098   (266) --------------------------------------------------------------------------------
  BnaA03g48770D:BnaCOMT1-3    (86) --------------------------------------------------------------------------------
                Bra019031    (87) --------------------------------------------------------------------------------
```

```
 BnaA10g07270D:BnaCOMT1-5  (1710) ---AGTGTGTCTGTG------TGTGTTTACTTAA---AACCATG------CCAACATGACTTGTTATAG-----------
                Bra003007  (1712) ---AGTGTGTCTGTG------TGTGTTTACTTAA---AACCATG------CCAACATGACTTGTTATAG-----------
BnaC09g30360D:BnaCOMT1-10  (1617) CCAACTAGGTATTTCAAAAAATTTCAATACGTAATGGATCCGGGTATTTCCTAAAAAGATTTGAAGTATCTGAAAACTCA
                Bol038837  (1617) CCAACTAGGTATTTCAAAAAATTTCAATACGTAATGGATCCGGGTATTTCCTAAAAAGATTTGAAGTATCTGAAAACTCA
 BnaA02g09810D:BnaCOMT1-2   (563) --------------------------------------------------------------------------------
                Bra022700   (562) --------------------------------------------------------------------------------
 BnaC02g13760D:BnaCOMT1-7   (526) --------------------------------------------------------------------------------
                Bol037465   (526) --------------------------------------------------------------------------------
 BnaA03g11990D:BnaCOMT1-4   (831) --------------------------------------------------------------------------------
                Bra029041   (831) --------------------------------------------------------------------------------
 BnaC03g14720D:BnaCOMT1-8  (1117) --------------------------------------------------------------------------------
                Bol027968  (1115) --------------------------------------------------------------------------------
 BnaA10g07250D:BnaCOMT1-6  (2713) --------------------------------------------------------------------------------
                Bra003009  (4256) --------------------------------------------------------------------------------
 BnaAnng13760D:BnaCOMT1-1   (246) --------------------------------------------------------------------------------
                Bra026320   (246) --------------------------------------------------------------------------------
 BnaC07g40930D:BnaCOMT1-9   (152) --------------------------------------------------------------------------------
                Bol042352   (240) --------------------------------------------------------------------------------
BnaC01g20290D:BnaCOMT1-12   (168) --------------------------------------------------------------------------------
                Bol013098   (266) --------------------------------------------------------------------------------
 BnaA03g48770D:BnaCOMT1-3    (86) --------------------------------------------------------------------------------
                Bra019031    (87) --------------------------------------------------------------------------------

 BnaA10g07270D:BnaCOMT1-5  (1761) --------------------------------------------------------------------------------
                Bra003007  (1763) --------------------------------------------------------------------------------
BnaC09g30360D:BnaCOMT1-10  (1697) AAATATCTGAAACTCCAAACAAATACCCGGAAATATTAAAATACCAAACTCCTCCTCCCCCCCCCCCCCCCCCTCCCAA
                Bol038837  (1697) AAATATCTGAAACTCCAAACAAATACCCGGAAATATTAAAATACCAAACTCCTCCTCCCCCCCCCCCCCCCCCTCCCAA
 BnaA02g09810D:BnaCOMT1-2   (563) --------------------------------------------------------------------------------
                Bra022700   (562) --------------------------------------------------------------------------------
 BnaC02g13760D:BnaCOMT1-7   (526) --------------------------------------------------------------------------------
                Bol037465   (526) --------------------------------------------------------------------------------
 BnaA03g11990D:BnaCOMT1-4   (831) --------------------------------------------------------------------------------
                Bra029041   (831) --------------------------------------------------------------------------------
 BnaC03g14720D:BnaCOMT1-8  (1117) --------------------------------------------------------------------------------
                Bol027968  (1115) --------------------------------------------------------------------------------
 BnaA10g07250D:BnaCOMT1-6  (2713) --------------------------------------------------------------------------------
                Bra003009  (4256) --------------------------------------------------------------------------------
 BnaAnng13760D:BnaCOMT1-1   (246) --------------------------------------------------------------------------------
                Bra026320   (246) --------------------------------------------------------------------------------
 BnaC07g40930D:BnaCOMT1-9   (152) --------------------------------------------------------------------------------
                Bol042352   (240) --------------------------------------------------------------------------------
BnaC01g20290D:BnaCOMT1-12   (168) --------------------------------------------------------------------------------
                Bol013098   (266) --------------------------------------------------------------------------------
 BnaA03g48770D:BnaCOMT1-3    (86) --------------------------------------------------------------------------------
                Bra019031    (87) --------------------------------------------------------------------------------

 BnaA10g07270D:BnaCOMT1-5  (1761) --------------------------------------------------------------------------------
                Bra003007  (1763) --------------------------------------------------------------------------------
BnaC09g30360D:BnaCOMT1-10  (1777) ATTTTACCTGAAATCTAACGCAAGAACCAAAAACACACTCAAAATTTTACCCGAATACCCAAAACAAAATTTAAACAAAA
                Bol038837  (1777) ATTTTACCTGAAATCTAACGCAAGAACCAAAAACACACTCAAAATTTTACCCGAATACCCAAAACAAAATTTAAACAAAA
 BnaA02g09810D:BnaCOMT1-2   (563) --------------------------------------------------------------------------------
                Bra022700   (562) --------------------------------------------------------------------------------
 BnaC02g13760D:BnaCOMT1-7   (526) --------------------------------------------------------------------------------
                Bol037465   (526) --------------------------------------------------------------------------------
 BnaA03g11990D:BnaCOMT1-4   (831) --------------------------------------------------------------------------------
                Bra029041   (831) --------------------------------------------------------------------------------
 BnaC03g14720D:BnaCOMT1-8  (1117) --------------------------------------------------------------------------------
                Bol027968  (1115) --------------------------------------------------------------------------------
 BnaA10g07250D:BnaCOMT1-6  (2713) --------------------------------------------------------------------------------
                Bra003009  (4256) --------------------------------------------------------------------------------
 BnaAnng13760D:BnaCOMT1-1   (246) --------------------------------------------------------------------------------
                Bra026320   (246) --------------------------------------------------------------------------------
 BnaC07g40930D:BnaCOMT1-9   (152) --------------------------------------------------------------------------------
                Bol042352   (240) --------------------------------------------------------------------------------
BnaC01g20290D:BnaCOMT1-12   (168) --------------------------------------------------------------------------------
                Bol013098   (266) --------------------------------------------------------------------------------
 BnaA03g48770D:BnaCOMT1-3    (86) --------------------------------------------------------------------------------
                Bra019031    (87) --------------------------------------------------------------------------------

 BnaA10g07270D:BnaCOMT1-5  (1761) --------------------------------------------------------------------------------
                Bra003007  (1763) --------------------------------------------------------------------------------
BnaC09g30360D:BnaCOMT1-10  (1857) TTGAAATTTTATTCAAAACCTGAAACTATCCTGAAAACCAAACCCAAATTTTTTTAAAAATATTCGCAATACAGAAAAAA
                Bol038837  (1857) TTGAAATTTTATTCAAAACCTGAAACTATCCTGAAAACCAAACCCAAATTTTTTTAAAAATATTCGCAATACAGAAAAAA
 BnaA02g09810D:BnaCOMT1-2   (563) --------------------------------------------------------------------------------
                Bra022700   (562) --------------------------------------------------------------------------------
 BnaC02g13760D:BnaCOMT1-7   (526) --------------------------------------------------------------------------------
                Bol037465   (526) --------------------------------------------------------------------------------
 BnaA03g11990D:BnaCOMT1-4   (831) --------------------------------------------------------------------------------
                Bra029041   (831) --------------------------------------------------------------------------------
 BnaC03g14720D:BnaCOMT1-8  (1117) --------------------------------------------------------------------------------
                Bol027968  (1115) --------------------------------------------------------------------------------
 BnaA10g07250D:BnaCOMT1-6  (2713) --------------------------------------------------------------------------------
                Bra003009  (4256) --------------------------------------------------------------------------------
 BnaAnng13760D:BnaCOMT1-1   (246) --------------------------------------------------------------------------------
                Bra026320   (246) --------------------------------------------------------------------------------
 BnaC07g40930D:BnaCOMT1-9   (152) --------------------------------------------------------------------------------
                Bol042352   (240) --------------------------------------------------------------------------------
BnaC01g20290D:BnaCOMT1-12   (168) --------------------------------------------------------------------------------
                Bol013098   (266) --------------------------------------------------------------------------------
 BnaA03g48770D:BnaCOMT1-3    (86) --------------------------------------------------------------------------------
                Bra019031    (87) --------------------------------------------------------------------------------
```

```
BnaA10g07270D:BnaCOMT1-5  (1761) --------------------------------------------------------------------------------
                Bra003007  (1763) --------------------------------------------------------------------------------
BnaC09g30360D:BnaCOMT1-10 (1937) TATTCGAGATACGCAAATATACCTAATATACACAAAATATTTTAGGGTTTTAGGTACCCAGTCGGATCTCGAGCAGGATC
                Bol038837  (1937) TATTCGAGATACGCAAATATACCTAATATACACAAAATATTTTAGGGTTTTAGGTACCCAGTCGGATCTCGAGCAGGATC
BnaA02g09810D:BnaCOMT1-2   (563) --------------------------------------------------------------------------------
                Bra022700   (562) --------------------------------------------------------------------------------
BnaC02g13760D:BnaCOMT1-7   (526) --------------------------------------------------------------------------------
                Bol037465   (526) --------------------------------------------------------------------------------
BnaA03g11990D:BnaCOMT1-4   (831) --------------------------------------------------------------------------------
                Bra029041   (831) --------------------------------------------------------------------------------
BnaC03g14720D:BnaCOMT1-8  (1117) --------------------------------------------------------------------------------
                Bol027968  (1115) --------------------------------------------------------------------------------
BnaA10g07250D:BnaCOMT1-6  (2713) --------------------------------------------------------------------------------
                Bra003009  (4256) --------------------------------------------------------------------------------
BnaAnng13760D:BnaCOMT1-1   (246) --------------------------------------------------------------------------------
                Bra026320   (246) --------------------------------------------------------------------------------
BnaC07g40930D:BnaCOMT1-9   (152) --------------------------------------------------------------------------------
                Bol042352   (240) --------------------------------------------------------------------------------
BnaC01g20290D:BnaCOMT1-12  (168) --------------------------------------------------------------------------------
                Bol013098   (266) --------------------------------------------------------------------------------
BnaA03g48770D:BnaCOMT1-3    (86) --------------------------------------------------------------------------------
                Bra019031    (87) --------------------------------------------------------------------------------

BnaA10g07270D:BnaCOMT1-5  (1761) --------------------------------------------------------------------------------
                Bra003007  (1763) --------------------------------------------------------------------------------
BnaC09g30360D:BnaCOMT1-10 (2017) CAAATTCAAACCAAAACCCAACTCAGACTCGAACCGAGACCCACAGGTCCAAAAAAAATACCCACTAAGTATTTTACCCT
                Bol038837  (2017) CAAATTCAAACCAAAACCCAACTCAGACTCGAACCGAGACCCACAGGTCCAAAAAAAATACCCACTAAGTATTTTACCCT
BnaA02g09810D:BnaCOMT1-2   (563) --------------------------------------------------------------------------------
                Bra022700   (562) --------------------------------------------------------------------------------
BnaC02g13760D:BnaCOMT1-7   (526) --------------------------------------------------------------------------------
                Bol037465   (526) --------------------------------------------------------------------------------
BnaA03g11990D:BnaCOMT1-4   (831) --------------------------------------------------------------------------------
                Bra029041   (831) --------------------------------------------------------------------------------
BnaC03g14720D:BnaCOMT1-8  (1117) --------------------------------------------------------------------------------
                Bol027968  (1115) --------------------------------------------------------------------------------
BnaA10g07250D:BnaCOMT1-6  (2713) --------------------------------------------------------------------------------
                Bra003009  (4256) --------------------------------------------------------------------------------
BnaAnng13760D:BnaCOMT1-1   (246) --------------------------------------------------------------------------------
                Bra026320   (246) --------------------------------------------------------------------------------
BnaC07g40930D:BnaCOMT1-9   (152) --------------------------------------------------------------------------------
                Bol042352   (240) --------------------------------------------------------------------------------
BnaC01g20290D:BnaCOMT1-12  (168) --------------------------------------------------------------------------------
                Bol013098   (266) --------------------------------------------------------------------------------
BnaA03g48770D:BnaCOMT1-3    (86) --------------------------------------------------------------------------------
                Bra019031    (87) --------------------------------------------------------------------------------

BnaA10g07270D:BnaCOMT1-5  (1761) --------------------------------------------------------------------------------
                Bra003007  (1763) --------------------------------------------------------------------------------
BnaC09g30360D:BnaCOMT1-10 (2097) GGATCCGGATCCGAACCAAACCTGTATTTTCAAGTCAACTTTAGTTTGGTTTCTCGGGTCCGGATAATATGTCAGGCCTA
                Bol038837  (2097) GGATCCGGATCCGAACCAAACCTGTATTTTCAAGTCAACTTTAGTTTGGTTTCTCGGGTCCGGATAATATGTCAGGCCTA
BnaA02g09810D:BnaCOMT1-2   (563) --------------------------------------------------------------------------------
                Bra022700   (562) --------------------------------------------------------------------------------
BnaC02g13760D:BnaCOMT1-7   (526) --------------------------------------------------------------------------------
                Bol037465   (526) --------------------------------------------------------------------------------
BnaA03g11990D:BnaCOMT1-4   (831) --------------------------------------------------------------------------------
                Bra029041   (831) --------------------------------------------------------------------------------
BnaC03g14720D:BnaCOMT1-8  (1117) --------------------------------------------------------------------------------
                Bol027968  (1115) --------------------------------------------------------------------------------
BnaA10g07250D:BnaCOMT1-6  (2713) --------------------------------------------------------------------------------
                Bra003009  (4256) --------------------------------------------------------------------------------
BnaAnng13760D:BnaCOMT1-1   (246) --------------------------------------------------------------------------------
                Bra026320   (246) --------------------------------------------------------------------------------
BnaC07g40930D:BnaCOMT1-9   (152) --------------------------------------------------------------------------------
                Bol042352   (240) --------------------------------------------------------------------------------
BnaC01g20290D:BnaCOMT1-12  (168) --------------------------------------------------------------------------------
                Bol013098   (266) --------------------------------------------------------------------------------
BnaA03g48770D:BnaCOMT1-3    (86) --------------------------------------------------------------------------------
                Bra019031    (87) --------------------------------------------------------------------------------

BnaA10g07270D:BnaCOMT1-5  (1761) --------------------------------------------------------------------------------
                Bra003007  (1763) --------------------------------------------------------------------------------
BnaC09g30360D:BnaCOMT1-10 (2177) GAAATAGCTACATAAAAGTGTACATACAAAAGTCTTAAATAAAATAATTTATAAATTCTATAGTTTTAAATATATTCATT
                Bol038837  (2177) GAAATAGCTACATAAAAGTGTACATACAAAAGTCTTAAATAAAATAATTTATAAATTCTATAGTTTTAAATATATTCATT
BnaA02g09810D:BnaCOMT1-2   (563) --------------------------------------------------------------------------------
                Bra022700   (562) --------------------------------------------------------------------------------
BnaC02g13760D:BnaCOMT1-7   (526) --------------------------------------------------------------------------------
                Bol037465   (526) --------------------------------------------------------------------------------
BnaA03g11990D:BnaCOMT1-4   (831) --------------------------------------------------------------------------------
                Bra029041   (831) --------------------------------------------------------------------------------
BnaC03g14720D:BnaCOMT1-8  (1117) --------------------------------------------------------------------------------
                Bol027968  (1115) --------------------------------------------------------------------------------
BnaA10g07250D:BnaCOMT1-6  (2713) --------------------------------------------------------------------------------
                Bra003009  (4256) --------------------------------------------------------------------------------
BnaAnng13760D:BnaCOMT1-1   (246) --------------------------------------------------------------------------------
                Bra026320   (246) --------------------------------------------------------------------------------
BnaC07g40930D:BnaCOMT1-9   (152) --------------------------------------------------------------------------------
                Bol042352   (240) --------------------------------------------------------------------------------
BnaC01g20290D:BnaCOMT1-12  (168) --------------------------------------------------------------------------------
                Bol013098   (266) --------------------------------------------------------------------------------
BnaA03g48770D:BnaCOMT1-3    (86) --------------------------------------------------------------------------------
                Bra019031    (87) --------------------------------------------------------------------------------
```

```
BnaA10g07270D:BnaCOMT1-5  (1761) --------------------------------------------------------------------------------
               Bra003007  (1763) --------------------------------------------------------------------------------
BnaC09g30360D:BnaCOMT1-10 (2257) TTCAGTATAATACGAATTTGTAACATAAATATATAGCAATTTTAAAATACTTATTCATATTAAACTTTGTTTACATTCAA
               Bol038837  (2257) TTCAGTATAATACGAATTTGTAACATAAATATATAGCAATTTTAAAATACTTATTCATATTAAACTTTGTTTACATTCAA
BnaA02g09810D:BnaCOMT1-2   (563) --------------------------------------------------------------------------------
               Bra022700   (562) --------------------------------------------------------------------------------
BnaC02g13760D:BnaCOMT1-7   (526) --------------------------------------------------------------------------------
               Bol037465   (526) --------------------------------------------------------------------------------
BnaA03g11990D:BnaCOMT1-4   (831) --------------------------------------------------------------------------------
               Bra029041   (831) --------------------------------------------------------------------------------
BnaC03g14720D:BnaCOMT1-8  (1117) --------------------------------------------------------------------------------
               Bol027968  (1115) --------------------------------------------------------------------------------
BnaA10g07250D:BnaCOMT1-6  (2713) --------------------------------------------------------------------------------
               Bra003009  (4256) --------------------------------------------------------------------------------
BnaAnng13760D:BnaCOMT1-1   (246) --------------------------------------------------------------------------------
               Bra026320   (246) --------------------------------------------------------------------------------
BnaC07g40930D:BnaCOMT1-9   (152) --------------------------------------------------------------------------------
               Bol042352   (240) --------------------------------------------------------------------------------
BnaC01g20290D:BnaCOMT1-12  (168) --------------------------------------------------------------------------------
               Bol013098   (266) --------------------------------------------------------------------------------
BnaA03g48770D:BnaCOMT1-3    (86) --------------------------------------------------------------------------------
               Bra019031    (87) --------------------------------------------------------------------------------

BnaA10g07270D:BnaCOMT1-5  (1761) --------------------------------------------------------------------------------
               Bra003007  (1763) --------------------------------------------------------------------------------
BnaC09g30360D:BnaCOMT1-10 (2337) AAAAAAAGAATCCGGTCAAAATCTAGTATGGCTTAAAATAGAGAAGATGTAAGAGTAACAATTTAAGGAGTTGATTCGGA
               Bol038837  (2337) AAAAAAAGAATCCGGTCAAAATCTAGTATGGCTTAAAATAGAGAAGATGTAAGAGTAACAATTTAAGGAGTTGATTCGGA
BnaA02g09810D:BnaCOMT1-2   (563) --------------------------------------------------------------------------------
               Bra022700   (562) --------------------------------------------------------------------------------
BnaC02g13760D:BnaCOMT1-7   (526) --------------------------------------------------------------------------------
               Bol037465   (526) --------------------------------------------------------------------------------
BnaA03g11990D:BnaCOMT1-4   (831) --------------------------------------------------------------------------------
               Bra029041   (831) --------------------------------------------------------------------------------
BnaC03g14720D:BnaCOMT1-8  (1117) --------------------------------------------------------------------------------
               Bol027968  (1115) --------------------------------------------------------------------------------
BnaA10g07250D:BnaCOMT1-6  (2713) --------------------------------------------------------------------------------
               Bra003009  (4256) --------------------------------------------------------------------------------
BnaAnng13760D:BnaCOMT1-1   (246) --------------------------------------------------------------------------------
               Bra026320   (246) --------------------------------------------------------------------------------
BnaC07g40930D:BnaCOMT1-9   (152) --------------------------------------------------------------------------------
               Bol042352   (240) --------------------------------------------------------------------------------
BnaC01g20290D:BnaCOMT1-12  (168) --------------------------------------------------------------------------------
               Bol013098   (266) --------------------------------------------------------------------------------
BnaA03g48770D:BnaCOMT1-3    (86) --------------------------------------------------------------------------------
               Bra019031    (87) --------------------------------------------------------------------------------

BnaA10g07270D:BnaCOMT1-5  (1761) --------------------------------------------------------------------------------
               Bra003007  (1763) --------------------------------------------------------------------------------
BnaC09g30360D:BnaCOMT1-10 (2417) ACCTAATATAAGAAGAGAATCGGTCTTCCAGTTTTTGAGCAACAAGAAAAGAAAATGGAAAACTTATATGATCTCTGGTT
               Bol038837  (2417) ACCTAATATAAGAAGAGAATCGGTCTTCCAGTTTTTGAGCAACAAGAAAAGAAAATGGAAAACTTATATGATCTCTGGTT
BnaA02g09810D:BnaCOMT1-2   (563) --------------------------------------------------------------------------------
               Bra022700   (562) --------------------------------------------------------------------------------
BnaC02g13760D:BnaCOMT1-7   (526) --------------------------------------------------------------------------------
               Bol037465   (526) --------------------------------------------------------------------------------
BnaA03g11990D:BnaCOMT1-4   (831) --------------------------------------------------------------------------------
               Bra029041   (831) --------------------------------------------------------------------------------
BnaC03g14720D:BnaCOMT1-8  (1117) --------------------------------------------------------------------------------
               Bol027968  (1115) --------------------------------------------------------------------------------
BnaA10g07250D:BnaCOMT1-6  (2713) --------------------------------------------------------------------------------
               Bra003009  (4256) --------------------------------------------------------------------------------
BnaAnng13760D:BnaCOMT1-1   (246) --------------------------------------------------------------------------------
               Bra026320   (246) --------------------------------------------------------------------------------
BnaC07g40930D:BnaCOMT1-9   (152) --------------------------------------------------------------------------------
               Bol042352   (240) --------------------------------------------------------------------------------
BnaC01g20290D:BnaCOMT1-12  (168) --------------------------------------------------------------------------------
               Bol013098   (266) --------------------------------------------------------------------------------
BnaA03g48770D:BnaCOMT1-3    (86) --------------------------------------------------------------------------------
               Bra019031    (87) --------------------------------------------------------------------------------

BnaA10g07270D:BnaCOMT1-5  (1761) --------------------------------------------------------------------------------
               Bra003007  (1763) --------------------------------------------------------------------------------
BnaC09g30360D:BnaCOMT1-10 (2497) TATGTTTTGGTTTCCTACTATTGAACCGGCTGGTTCTCTTTGCATAGCGGCTGTACATCAGATTCACCAATTAAACTAAA
               Bol038837  (2497) TATGTTTTGGTTTCCTACTATTGAACCGGCTGGTTCTCTTTGCATAGCGGCTGTACATCAGATTCACCAATTAAACTAAA
BnaA02g09810D:BnaCOMT1-2   (563) --------------------------------------------------------------------------------
               Bra022700   (562) --------------------------------------------------------------------------------
BnaC02g13760D:BnaCOMT1-7   (526) --------------------------------------------------------------------------------
               Bol037465   (526) --------------------------------------------------------------------------------
BnaA03g11990D:BnaCOMT1-4   (831) --------------------------------------------------------------------------------
               Bra029041   (831) --------------------------------------------------------------------------------
BnaC03g14720D:BnaCOMT1-8  (1117) --------------------------------------------------------------------------------
               Bol027968  (1115) --------------------------------------------------------------------------------
BnaA10g07250D:BnaCOMT1-6  (2713) --------------------------------------------------------------------------------
               Bra003009  (4256) --------------------------------------------------------------------------------
BnaAnng13760D:BnaCOMT1-1   (246) --------------------------------------------------------------------------------
               Bra026320   (246) --------------------------------------------------------------------------------
BnaC07g40930D:BnaCOMT1-9   (152) --------------------------------------------------------------------------------
               Bol042352   (240) --------------------------------------------------------------------------------
BnaC01g20290D:BnaCOMT1-12  (168) --------------------------------------------------------------------------------
               Bol013098   (266) --------------------------------------------------------------------------------
BnaA03g48770D:BnaCOMT1-3    (86) --------------------------------------------------------------------------------
               Bra019031    (87) --------------------------------------------------------------------------------
```

```
BnaA10g07270D:BnaCOMT1-5  (1761) --------------------------------------------------------------------------------
                Bra003007  (1763) --------------------------------------------------------------------------------
BnaC09g30360D:BnaCOMT1-10  (2577) CTGACCTGGATAAAGCCCTGATCATATAAACTAGAGTTCAATCTTGATATCAGTTTTAAAACAGATTATCCTAATAATTG
                Bol038837  (2577) CTGACCTGGATAAAGCCCTGATCATATAAACTAGAGTTCAATCTTGATATCAGTTTTAAAACAGATTATCCTAATAATTG
 BnaA02g09810D:BnaCOMT1-2   (563) --------------------------------------------------------------------------------
                Bra022700   (562) --------------------------------------------------------------------------------
 BnaC02g13760D:BnaCOMT1-7   (526) --------------------------------------------------------------------------------
                Bol037465   (526) --------------------------------------------------------------------------------
 BnaA03g11990D:BnaCOMT1-4   (831) --------------------------------------------------------------------------------
                Bra029041   (831) --------------------------------------------------------------------------------
 BnaC03g14720D:BnaCOMT1-8  (1117) --------------------------------------------------------------------------------
                Bol027968  (1115) --------------------------------------------------------------------------------
 BnaA10g07250D:BnaCOMT1-6  (2713) --------------------------------------------------------------------------------
                Bra003009  (4256) --------------------------------------------------------------------------------
 BnaAnng13760D:BnaCOMT1-1   (246) --------------------------------------------------------------------------------
                Bra026320   (246) --------------------------------------------------------------------------------
 BnaC07g40930D:BnaCOMT1-9   (152) --------------------------------------------------------------------------------
                Bol042352   (240) --------------------------------------------------------------------------------
BnaC01g20290D:BnaCOMT1-12   (168) --------------------------------------------------------------------------------
                Bol013098   (266) --------------------------------------------------------------------------------
 BnaA03g48770D:BnaCOMT1-3    (86) --------------------------------------------------------------------------------
                Bra019031    (87) --------------------------------------------------------------------------------

 BnaA10g07270D:BnaCOMT1-5  (1761) --------------------------------------------------------------------------------
                Bra003007  (1763) --------------------------------------------------------------------------------
BnaC09g30360D:BnaCOMT1-10  (2657) ATGAACCAAAAGTTGAACAGTTTAGGTTAAAGCTTAAGGAGTGTGTGTGTGTTTACTTAAAATCATGCCAACATGACTTG
                Bol038837  (2657) ATGAACCAAAAGTTGAACAGTTTAGGTTAAAGCTTAAGGAGTGTGTGTGTGTTTACTTAAAATCATGCCAACATGACTTG
 BnaA02g09810D:BnaCOMT1-2   (563) --------------------------------------------------------------------------------
                Bra022700   (562) --------------------------------------------------------------------------------
 BnaC02g13760D:BnaCOMT1-7   (526) --------------------------------------------------------------------------------
                Bol037465   (526) --------------------------------------------------------------------------------
 BnaA03g11990D:BnaCOMT1-4   (831) --------------------------------------------------------------------------------
                Bra029041   (831) --------------------------------------------------------------------------------
 BnaC03g14720D:BnaCOMT1-8  (1117) --------------------------------------------------------------------------------
                Bol027968  (1115) --------------------------------------------------------------------------------
 BnaA10g07250D:BnaCOMT1-6  (2713) --------------------------------------------------------------------------------
                Bra003009  (4256) --------------------------------------------------------------------------------
 BnaAnng13760D:BnaCOMT1-1   (246) --------------------------------------------------------------------------------
                Bra026320   (246) --------------------------------------------------------------------------------
 BnaC07g40930D:BnaCOMT1-9   (152) --------------------------------------------------------------------------------
                Bol042352   (240) --------------------------------------------------------------------------------
BnaC01g20290D:BnaCOMT1-12   (168) --------------------------------------------------------------------------------
                Bol013098   (266) --------------------------------------------------------------------------------
 BnaA03g48770D:BnaCOMT1-3    (86) --------------------------------------------------------------------------------
                Bra019031    (87) --------------------------------------------------------------------------------

 BnaA10g07270D:BnaCOMT1-5  (1761) --------------------------------------------------------------------------------
                Bra003007  (1763) --------------------------------------------------------------------------------
BnaC09g30360D:BnaCOMT1-10  (2737) TTATAG--------------------------------------------------------------------------
                Bol038837  (2737) TTATAG--------------------------------------------------------------------------
 BnaA02g09810D:BnaCOMT1-2   (563) --------------------------------------------------------------------------------
                Bra022700   (562) --------------------------------------------------------------------------------
 BnaC02g13760D:BnaCOMT1-7   (526) --------------------------------------------------------------------------------
                Bol037465   (526) --------------------------------------------------------------------------------
 BnaA03g11990D:BnaCOMT1-4   (831) --------------------------------------------------------------------------------
                Bra029041   (831) --------------------------------------------------------------------------------
 BnaC03g14720D:BnaCOMT1-8  (1117) --------------------------------------------------------------------------------
                Bol027968  (1115) --------------------------------------------------------------------------------
 BnaA10g07250D:BnaCOMT1-6  (2713) --------------------------------------------------------------------------------
                Bra003009  (4256) --------------------------------------------------------------------------------
 BnaAnng13760D:BnaCOMT1-1   (246) --------------------------------------------------------------------------------
                Bra026320   (246) --------------------------------------------------------------------------------
 BnaC07g40930D:BnaCOMT1-9   (152) --------------------------------------------------------------------------------
                Bol042352   (240) --------------------------------------------------------------------------------
BnaC01g20290D:BnaCOMT1-12   (168) --------------------------------------------------------------------------------
                Bol013098   (266) --------------------------------------------------------------------------------
 BnaA03g48770D:BnaCOMT1-3    (86) --------------------------------------------------------------------------------
                Bra019031    (87) --------------------------------------------------------------------------------
```
