# Supplementary material for: Cloning and Phylogenetic Analysis of Brassica napus L. Caffeic Acid O-Methyltransferase 1 Gene Family and Its Expression Pattern under Drought Stress
Source: PLoS One. 2016 Nov 10;11(11):e0165975. doi: 10.1371/journal.pone.0165975 (PMC5104432; doi:10.1371/journal.pone.0165975)
Supplement: S1 Table — (DOCX) [file pone.0165975.s003.docx]

**S1 Table.** Primers for gene cloning and qRT-PCR

| Name of primers | Sequence of primers (5'-3') | | Gene |
| --- | --- | --- | --- |
| Primers for gene cloning | | | |
| FCOMT-1 | 5'-ATGGCGGATACACAGATAACTCCACAAC-3' | | *BnCOMT1-2* |
| FCOMT-2 | 5’-ATGGGATCAACGGCGGAGACAC-3’ | | *BnCOMT1-4/BnCOMT1-8* |
| FCOMT-3 | 5’-ATGGGGTTAACGGAGGAGACACAGAT-3’ | | *BnCOMT1-5* |
| FCOMT-4 | 5’-ATGGGGTCAACGGAGGAGACACAG-3’ | *BnCOMT1-10* | |
| RCOMT-1 | 5'-TTAGTCGATCTTCTTGAGCAACTCGATAATG-3' | *BnCOMT1-2* | |
| RCOMT-2 | 5’-TTACATCTTTTTGAGCAGCTCAATAACG-3’ | *BnCOMT1-4/BnCOMT1-8* | |
| RCOMT-3 | 5’-TTAGATCTTCTTGAGCAACTCAATAACGTAAAC-3’ | *BnCOMT1-5/BnCOMT1-10* | |
| Primers for qRT-PCR | | | |
| FCOMT-2 Q | 5'-ACAACAAGTAACCAACGACGACGAA-3' | | *BnCOMT1-2* |
| FCOMT-3 Q | 5'-CGGAGGTAAGGAACGTACCGAGGAAG-3' | | *BnCOMT1-3* |
| FCOMT-4 Q | 5'-AGCCTCTCGACCAAACAAGTAGTCCATG-3' | | *BnCOMT1-4* |
| FCOMT-5 Q | 5'-AGTCGTACTATTCCCGGCGGC-3' | | *BnCOMT1-5* |
| FCOMT-6 Q | 5'-CTCAACGGCAGAGACACATTCAACT-3' | | *BnCOMT1-6* |
| FCOMT-8 Q | 5'-AGCCTCTCGACTAAACAAGTAGTCCATG-3' | | *BnCOMT1-8* |
| FCOMT-9 Q | 5'-AGAAGGAGTTTGAGGCTTTGGCCAA-3' | | *BnCOMT1-9* |
| FCOMT-10 Q | 5'-AGTCGTGCTATTCCCGGCG-3' | | *BnCOMT1-10* |
| FCOMT-11 Q | 5'-GTTTGAGTACCATGGGACTGACCTTAG-3' | | *BnCOMT1-11* |
| FCOMT-12 Q | 5'-ATTCCTTAAGTGGGTYTGCCATGAC-3' | | *BnCOMT1-12* |
| RCOMT-2 Q | 5'-TCGATACGAAGTTGAGAAGCAATCTC-3' | | *BnCOMT1-2* |
| RCOMT-3 (9) Q | 5'-TTATATCTTCTTGAGGAACTCAATGATGTG-3' | | *BnCOMT1-3/BnCOMT1-9* |
| RCOMT-4 (8) Q | 5'-CGTAAACACCAAAAGCATTGCAGGC-3' | | *BnCOMT1-4/BnCOMT1-8* |
| RCOMT-5 (10) Q | 5'-GGACACACCATCCTCGTTCTTG-3' | | *BnCOMT1-5/BnCOMT1-10* |
| RCOMT-6 Q | 5'-TAGCTGATTTTAAAGCCATAGGGAGAACTG-3' | | *BnCOMT1-6* |
| RCOMT-11 Q | 5'-GACCAAACCCTCAAAACCATTATAG-3' | | *BnCOMT1-11* |
| RCOMT-12 Q | 5'-ATGTGTGAGCCATCATGATACAGTCCAAG-3' | | *BnCOMT1-12* |
