# Supplementary material for: Cloning and Phylogenetic Analysis of Brassica napus L. Caffeic Acid O-Methyltransferase 1 Gene Family and Its Expression Pattern under Drought Stress
Source: PLoS One. 2016 Nov 10;11(11):e0165975. doi: 10.1371/journal.pone.0165975 (PMC5104432; doi:10.1371/journal.pone.0165975)
Supplement: S2 Table — (DOCX) [file pone.0165975.s004.docx]

**S2 Table.** *COMT* genes accession numbers in *A. thaliana*, *B. napus*, *B. rapa*, *B. oleracea*.

| Name in TAIR | Nomenclature | Name in Genescope | Subgenome | Counterpart name in BRAD | Subgenome |
| --- | --- | --- | --- | --- | --- |
| *At5g54160* | *BnCOMT1-1* | *BnaAnng13760D* |  | *Bra026320* |  |
|  | *BnCOMT1-2* | *BnaA02g09810D* | MF2 | *Bra022700* | MF2 |
|  | *BnCOMT1-3* | *BnaA03g48770D* |  | *Bra019031* |  |
|  | *BnCOMT1-4* | *BnaA03g11990D* | MF1 | *Bra029041* | MF1 |
|  | *BnCOMT1-5* | *BnaA10g07270D* | LF | *Bra003007* | LF |
|  | *BnCOMT1-6* | *BnaA10g07250D* | LF | *Bra003009* | LF |
|  | *BnCOMT1-7* | *BnaC02g13760D* | MF2 | *Bol037465* | MF2 |
|  | *BnCOMT1-8* | *BnaC03g14720D* | MF1 | *Bol027968* | MF1 |
|  | *BnCOMT1-9* | *BnaC07g40930D* |  | *Bol042352* |  |
|  | *BnCOMT1-10* | *BnaC09g30560D* |  | *Bol038837* |  |
|  | *BnCOMT1-11* | *BnaC09g30540D* |  | *Bol038834* |  |
|  | *BnCOMT1-12* | *BnaC01g20290D* |  | *Bol013098* |  |
| *At1g21100* |  | *BnaA07g11060D* |  | *Bra012270* | MF2 |
| *At1g21110* |  | *BnaA07g11080D* |  | *Bra012268* | MF2 |
| *At1g21120* |  | *BnaA07g11070D* |  | *Bra012269* | MF2 |
| *At1g21130* |  | *BnaA06g14960D* |  | *Bra025876* | LF |
|  |  | *BnaA08g21450D* |  | *Bra016432* | MF1 |
|  |  | *BnaA06g14950D* |  | *Bra025875* | LF |
|  |  | *BnaA06g14930D* |  | *Bra025874* | LF |
|  |  | *BnaA08g21470D* |  | *Bra016433* | MF1 |
|  |  | *BnaC08g19680D* |  | *Bol007029* | MF1 |
|  |  | *BnaC05g16350D* |  | *Bol001904* | LF |
|  |  | *BnaC07g14620D* |  | *Bol025493* | MF2 |
|  |  | *BnaC07g14640D* |  | *Bol025494* | MF2 |
|  |  | *BnaC07g14650D* |  | *Bol025495* | MF2 |
|  |  | *BnaC08g19690D* |  | *Bol007030* | MF1 |
| *At1g33030* |  | *BnaA08g06720D* | MF1 | *Bra035481* | MF1 |
| Name in TAIR | Nomenclature | Name in Genescope | Subgenome | Counterpart name in BRAD | Subgenome |
|  |  | *BnaC08g07320D* | MF1 | *Bol014175* | MF1 |
| *At1g51990* |  | *BnaA08g29990D* |  | *Bra035482* | MF1 |
|  |  | *BnaC08g07380D* |  | *Bol014177* | MF1 |
| *At1g63140* |  | *BnaA09g12650D* | MF1 | *Bra027815* | MF1 |
|  |  | *BnaC09g51230D* |  | *Bol022596* | MF1 |
| *At1g76790* |  | *BnaA07g33060D* | LF | *Bra015719* | LF |
|  |  | *BnaA07g21250D* | MF2 | *Bra003707* | MF2 |
|  |  | *BnaC06g21620D* | MF2 | *Bol039840* | MF2 |
|  |  | *BnaC06g37610D* | LF | *Bol027603* | LF |
| *At1g77520* |  | *BnaA07g20960D* |  | *Bra003682* | MF2 |
|  |  | *BnaA02g18290D* |  | *Bra008316* | MF1 |
|  |  | *BnaA07g20940D* |  | *Bra003680* | MF2 |
|  |  | *BnaA07g33600D* |  | *Bra015664* | LF |
|  |  | *_* |  | *Bol019013* | MF1 |
|  |  | *BnaC06g38210D* |  | *Bol027548* | LF |
|  |  | *BnaC06g20930D* |  | *Bol001491* | MF2 |
|  |  | *BnaA07g20960D* |  | *Bol001492* | MF2 |
| *At1g77530* |  | *BnaA07g20950D* |  | *Bra003681* | MF2 |
| *At3g53140* |  | *BnaA04g04870D* | MF1 | *Bra040689* | MF1 |
|  |  | *BnaC04g27590D* |  | *Bol004412* | MF1 |
| *At5g37170* |  |  |  |  |  |
| *At5g53810* |  |  |  |  |  |
