# Supplementary material for: Cloning and Phylogenetic Analysis of Brassica napus L. Caffeic Acid O-Methyltransferase 1 Gene Family and Its Expression Pattern under Drought Stress
Source: PLoS One. 2016 Nov 10;11(11):e0165975. doi: 10.1371/journal.pone.0165975 (PMC5104432; doi:10.1371/journal.pone.0165975)
Supplement: S3 Table — (DOCX) [file pone.0165975.s005.docx]

**S3 Table.** Main structural features of the *BnCOMT1* genes.

| Gene | DNA length(bp) | Intron | | ORF | |
| --- | --- | --- | --- | --- | --- |
|  |  | Position and length(bp) | (G+C)％ | length(bp) | (G+C)％ |
| *BnCOMT1-1* | 1332 | Ⅰ432-676(245) | 26.12 | 891 | 46.46 |
|  |  | Ⅱ988-1086(99) | 30.30 |  |  |
|  |  | Ⅲ1152-1248(97) | 22.68 |  |  |
| *BnCOMT1-2* | 1864 | Ⅰ399-960(562) | 27.76 | 1086 | 44.57 |
|  |  | Ⅱ1275-1366(92) | 29.35 |  |  |
|  |  | Ⅲ1432-1555(124) | 31.45 |  |  |
| *BnCOMT1-3* | 1379 | Ⅰ426-510(85) | 21.18 | 1104 | 48.46 |
|  |  | Ⅱ822-900(78) | 29.49 |  |  |
|  |  | Ⅲ965-1076(112) | 30.36 |  |  |
| *BnCOMT1-4* | 2126 | Ⅰ417-1246(830) | 30.60 | 1095 | 47.03 |
|  |  | Ⅱ1558-1649(92) | 20.65 |  |  |
|  |  | Ⅲ1715-1823(109) | 28.44 |  |  |
| *BnCOMT1-5* | 3057 | Ⅰ417-2176(1760) | 25.8 | 1095 | 46.67 |
|  |  | Ⅱ2488-2571(84) | 26.19 |  |  |
|  |  | Ⅲ2637-2754(118) | 27.12 |  |  |
| *BnCOMT1-6* | 4017 | Ⅰ411-3122(2712) | 24.41 | 1089 | 46.28 |
|  |  | Ⅱ3434-3540(107) | 26.17 |  |  |
|  |  | Ⅲ3606-3714(109) | 30.28 |  |  |
| *BnCOMT1-7* | 1383 | Ⅰ399-923(525) | 27.80 | 771 | 43.9 |
|  |  | Ⅱ1243-1329(87) | 27.50 |  |  |
| *BnCOMT1-8* | 2412 | Ⅰ417-1532(1116) | 28.32 | 1095 | 47.58 |
|  |  | Ⅱ1844-1935(92) | 19.57 |  |  |
|  |  | Ⅲ2001-2109(109) | 27.52 |  |  |
| *BnCOMT1-9* | 1446 | Ⅰ426-576(151) | 19.21 | 1104 | 48.37 |
|  |  | Ⅱ888-968(81) | 30.86 |  |  |
|  |  | Ⅲ1034-1143(110) | 30.91 |  |  |
| *BnCOMT1-10* | 4037 | Ⅰ417-3158(2742) | 28.23 | 1095 | 47.12 |
|  |  | Ⅱ3470-3555(86) | 27.91 |  |  |
|  |  | Ⅲ3621-3734(114) | 29.82 |  |  |
| *BnCOMT1-11* | 833 | Ⅰ254-361(108) | 25.00 | 621 | 43.32 |
|  |  | Ⅱ427-530(104) | 31.73 |  |  |
| *BnCOMT1-12* | 1447 | Ⅰ391-575(184) | 27.72 | 1059 | 45.99 |
|  |  | Ⅱ876-965(90) | 27.78 |  |  |
|  |  | Ⅲ1031-1144(114) | 25.44 |  |  |
